# Bio-process development for sustainable, large-scale production of LAB-oligosaccharides as a potential anti-colon-cancer metabolites produced by *Lactobacillus acidophilus *20079

**DOI:** 10.1186/s12934-026-03067-x

**Published:** 2026-08-12

**Authors:** Ahmed M. Kenawy, Gaber A. Abo-Zaid, Tarek H. Taha, Amany E. Ragab, Lamiaa A. Al-Madboly, Nehal M. El-Deeb

**Affiliations:** 1 Nucleic Acids Research Department, Genetic Engineering and Biotechnology Research Institute, City of Scientific Research and Technological Applications (SRTA-City), New Borg El-Arab City, Alexandria Egypt; 2 Bioprocess Development Department, Genetic Engineering and Biotechnology Research Institute, City of Scientific Research and Technological Applications (SRTA-City), New Borg El-Arab City, Alexandria Egypt; 3 Department of Biology, College of Science, Imam Mohammad Ibn Saud Islamic University (IMSIU), Riyadh, 11623 Saudi Arabia; 4 Pharmacognosy Department, Faculty of Pharmacy, Tanta University, Tanta, Egypt; 5 Department of Microbiology and Immunology, Faculty of Pharmacy, Tanta University, Tanta, Egypt; 6 Pharmaceutical Bioproducts Research Department, Genetic Engineering and Biotechnology Research Institute, City of Scientific Research and Technological Applications (SRTA-City), New Borg El-Arab City, Alexandria Egypt

## Abstract

Lactic acid bacteria and their metabolites have shown promising biological activities against different types of cancer, esspecially, colon cancer. In our previouse study, we extracted and optimized the production of a mixture of three oligosaccharides from *Lactobacillus acidophilus* DSMZ 20079 at the laboratory scale using statistical experimental designs and the active oligosaccharide HPLC fraction was identified as LA-EPS-20079. Also, the anticancer activity of the extracted LA-EPS-20079 against colon cancer CaCo-2 cell line was confirmed via the downregulation of the gene expression of *Bcl2* and *Survivin* genes (6). In the present study, we produced a mixture of bioactive oligosaccharides from *L. acidophilus* DSMZ 20079 in 10 L bioreactor that maintained the anti-colon cancer against CaCo-2 cell line effects, which is a major transition towards the semi industrial level. To that end, a batch fermentation was carried out in a 10 L bioreactor under uncontrolled pH condition, starting at pH 6.0. During the fermentation process, the bacterial cell mass was increased exponentially with a constant specific growth rate (µ) of 0.07 h^−1^, reaching a maximum biomass of 6.93 g L^−1^ at 44 h. Also, the concentration of the produced total exooligosaccharides gradually increased and reached a maximum concentration of 1461.43 µg mL^−1^ at 40 h post-incubation. Furthermore, the glucose consumption rate was 1.21 g L^−1^ h^−1^, while the LAB-oligosaccharide production rate reached 96.22 µg mL^−1^ h^−1^. Moreover, oligosaccharides chemical composition was studied after scaling up the production process using 1D and 2D proton and carbon NMR, along with monomeric composition analysis. The results indicated that LAB-oligosaccharide is a mixture of oligosaccharides, not a single structure, and the mixture consists of three monomeric sugars; herein, we referred to the oligosaccharide mixture as LAB-oligosaccharide. Additionally, the anticancer activity of LAB- oligosaccharide was tested in samples collected from the bioreactor at 0, 12, 14, 16, 18, 20, 22, 24, 36, 40 and 44 h time points against the CaCo-2 cancer cell line. The results confirmed the in-vitro anticancer activity with maximum inhibition percentages of 72.01% and 81.7% after 22 and 24 h, respectively. On the other hand, to establish an environmentally friendly production process, we evaluated the efficacy of using the bacterial biomass residue after LAB- oligosaccharide extraction as a substrate for bacterial isolates growth to produce industrially important enzymes (Lipases and proteases). In this context, 5 bacterial isolates were found to produce both enzymes. These bacterial isolates were characterized using 16S-rRNA gene sequencing and the relatedness between the isolates was studied using phylogenetic analysis. The results demonstrated that the residual biomass supported the growth of *B. subtillis* PX205316 (2F) for the production of commercially valuable lipase and protease enzymes, hence, the recommended bioprocess was efficient in producing substrate with potential anticancer properties in a process that is environmentally friendly that promotes environmental sustainability.

**Graphical Abstract:**

**Figure:**
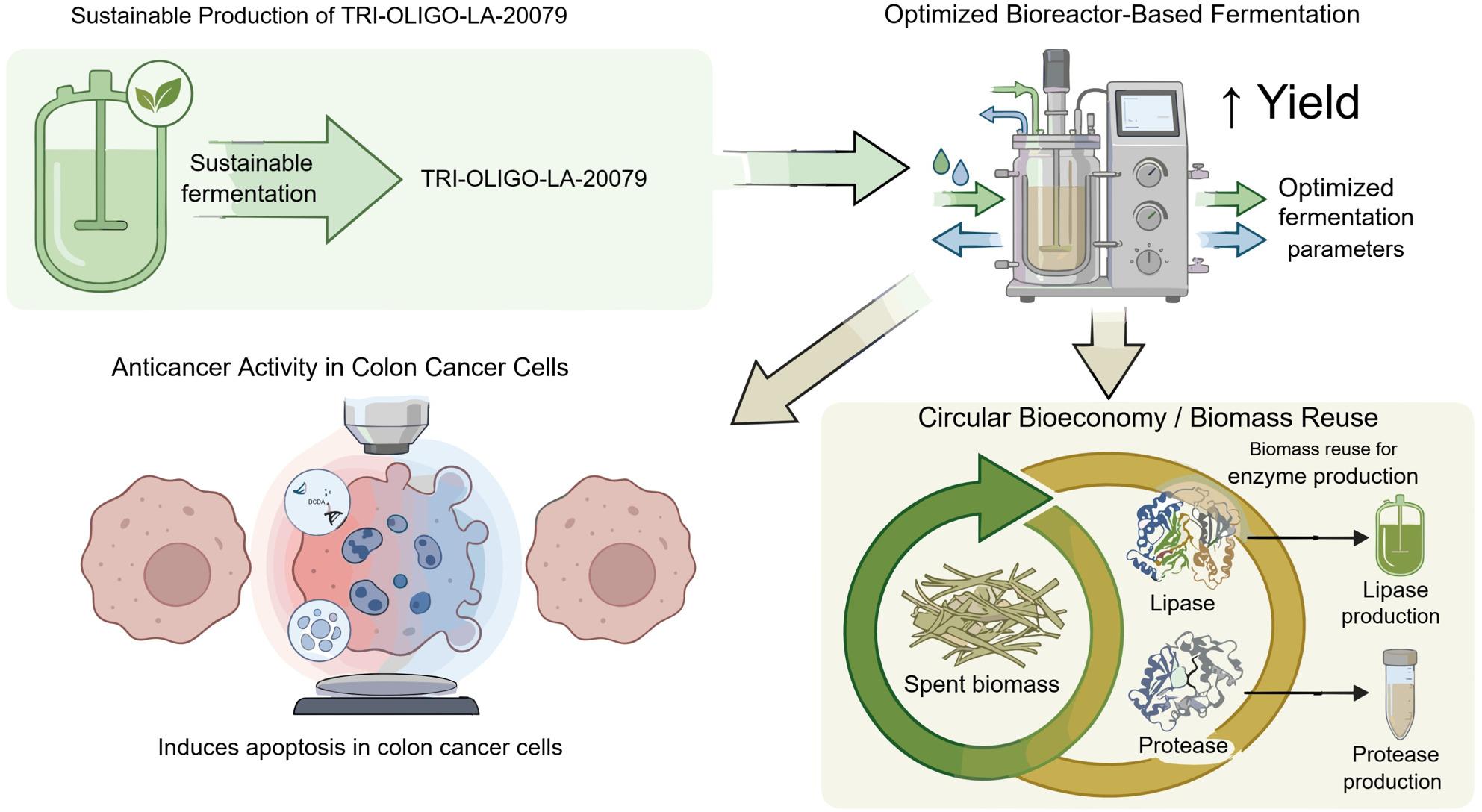

**Supplementary Information:**

The online version contains supplementary material available at 10.1186/s12934-026-03067-x.

## Introduction

Colorectal cancer (CRC) represents a critical oncological burden, being the third most diagnosed cancer disease, and globally, a leading cause of cancer mortality [1]. The limitations of the conventional chemotherapeutic regimens, including severe systemic toxicity, damaging healthy tissues, and the increasing prevalence of multidrug resistance underscore their value. Therfore, the fact that the world needs to develop novel, targeted, and biocompatible antitumor agents is imperative [1]. In recent years, the paradigm has shifted towards exploring the therapeutic potential of bioactives derived from natural sources, particularly those produced by commensal and probiotic microorganisms within the human gut microbiome [2].

**Figure:**
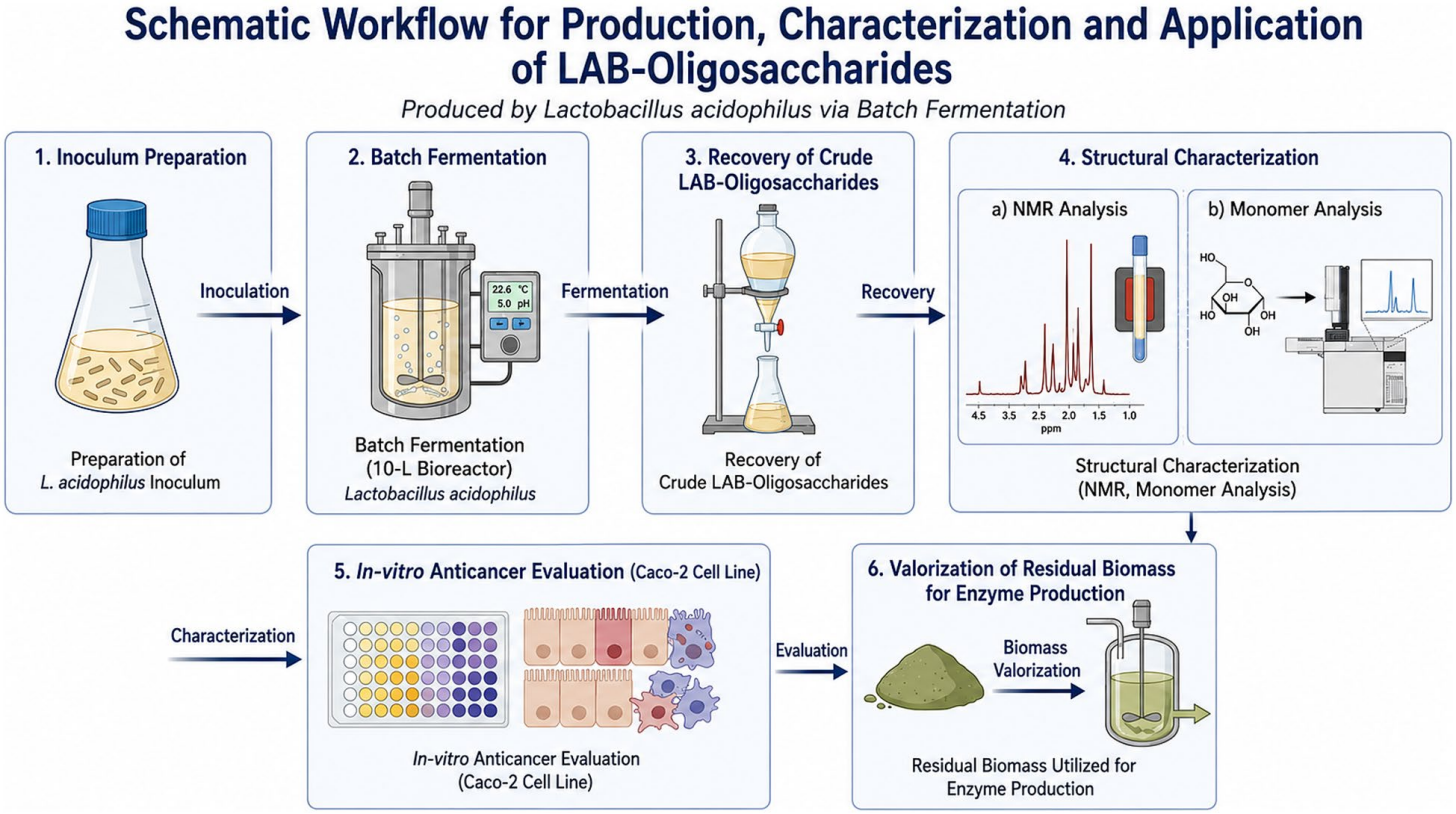

Human gastrointestinal tract hosts a complex ecosystem of microbes, and its dysbiosis has been closely linked to the pathogenesis of CRC, influencing processes such as inflammation, cell proliferation, and apoptosis [3]. This connection has propelled research into probiotic bacteria, notably Lactic Acid Bacteria (LAB), and their secreted metabolites as potential oncotherapeutic agents. Among these metabolites, oligosaccharides (OGS) have emerged as a promising class of low molecular weight bioactive carbohydrates. LAB-derived OGS are generally recognized as safe (GRAS) and exhibit a remarkable array of biological activities, including immunomodulation, antioxidant, antimicrobial, and antitumor effects [4–7].

*Lactobacillus acidophilus*, a cornerstone probiotic species, has demonstrated significant potential in this area. Previous work has established that oligosaccharide fractions from *L. acidophilus* can inhibit the growth of colon cancer cells [6]. A previous study further purified a novel exopolysaccharide, LA-EPS-20079, from *L. acidophilus* 20,079, demonstrating its unique ability to co-regulate pro-apoptotic and anti-inflammatory NF-κB pathways in human colon cancer cells [4]. This dual mechanism positions LA-EPS-20079 as a highly compelling multi-targeted therapeutic candidate; however, a critical translational gap remains. As recently reviewed [8], the vast majority of studies demonstrating anti-cancer effects of microbial polysaccharides and oligosaccharides are preclinical in nature, relying on in vitro cell line assays and in vivo animal models. A fundamental reason for this slow clinical progress is that the bioactivity of these carbohydrates is often indirect and microbiota-dependent: their fermentation by commensal gut bacteria produces active metabolites (e.g., short-chain fatty acids such as butyrate, propionate, and acetate) that modulate apoptosis, inflammation, and immune surveillance [4, 6, 8]. This reliance on an intact and functionally diverse gut microbiota introduces significant translational complexity, as inter-individual variability in microbiome composition, influenced by diet, genetics, age, and disease status, can lead to highly heterogeneous therapeutic responses in human populations. To date, no purified LAB-derived Exopolysaccharide/oligosaccharide (EPS/OGS) with claimed anti-colon cancer health effects has completed full clinical evaluation (i.e., Phase I–III trials) or received regulatory approval (e.g., FDA or EMA) as a standalone therapeutic for colorectal cancer or any other malignancy, and such translational progress remains explicitly limited to preclinical stages.

Statistical experimental designs offer powerful and efficient frameworks for overcoming the low production rate of microbial metabolites, enabling the systematic optimization of cultural and nutritional parameters to dramatically enhance OGS production titers without resorting to genetic modification [9]. Furthermore, the transition towards sustainable bioprocesses is paramount for the economic and environmental sustainability of such microbial technologies. A key principle of this transition is the adoption of circular bioeconomy principles, which aim to valorize waste streams from the industrial processes [10, 11].

In microbial fermentation, the spent biomass generated after metabolite extraction represents a significant bioresidual stream, which is rich in nitrogen and carbon. Recent studies have demonstrated the potential of such microbial biomass residues to be repurposed as effective nutrient (carbon and nitrogen) sources for secondary fermentation processes,, enabling the production of high-value microbial enzymes [10]. This strategy of microbial biorefinery not only minimizes environmental impact by reducing waste, but also enhances the overall sustainability and cost-effectiveness of bioprocess downstream practices.

Therefore, the aim of the present study is to systematically optimize the production of the potential anti-colon cancer LAB-oligosaccharides, from *Lactobacillus acidophilus* 20079 using a rigorous statistical experimental design approach to maximize yield. The structure of the produced LAB-oligosaccharides will be studied using NMR and mass-spectra analyses, and the anticolon cancer properties of the product will be in-vitro validated against the CaCo-2 human colon cancer cell line. Finally, down stream process will be carried out to valorize the process waste by converting the post-extraction biomass into a nutrient source for bacterial strain growth for important enzymes production. This integrated work bridges critical gaps between bioprocess development and potential oncotherapeutic discovery, advancing a promising microbial bioactive OGS as a strong candidate for preclinical and clinical studies towards future development of a functional food adjunct or a novel therapeutic agent. Simultaneously, the current study presenting a sustainable and economically viable biomanufacturing nuclus for piolt scal studies for industrial enzymes production.

## Materials and methods

### Batch fermentation process

The batch fermentation process, presented in the current work, was carried out in a 10-L bench-top bioreactor (Cleaver, Saratoga, USA) equipped with three 6-bladed disc-turbine impellers and four side baffles. The biprocess was controlled by a digital control unit. At the beginning, the working volume of the batch was 4 L. The batch fermentation process was performed at an established temperature of 30 °C and uncontrolled pH, which initially started at pH 6.0. Air was pumped at a rate of 1 VVM (volume of air/volume of broth/one minute) after passing through a sterile 0.2 µm Sartorius Filter Midisart 2000 PTFE (Sartorius, Germany). Agitation was controlled at 200 rpm, and the dissolved oxygen level and pH values were recorded online using Mettler Toledo electrodes.

For preparation of the bacterial inoculum, a single colony of 2 days-old culture plate of *L. acidophilus* DSMZ 20079 was inoculated into a 250 mL Erlenmeyer flask containing 50 mL of autoclaved MRS medium (HIMEDIA, India). The culture was incubated overnight at 30 °C with shaking at 200 rpm until reaching 2 × 10^9^ cfu. mL^−1^ in a New Brunswick Innova 42R Refrigerated Incubator Shaker (New Brunswick, Germany). The batch fermentation process was completed in a Cleaver Saratoga bioreactor (Cleaver Saratoga, NY, USA) using our group’s previously optimized production medium as follows. The medium components were purchased from Fisher company (Fisher, USA), except agar–agar that was purchased from BioLabs (BioLabs, USA). Each component was weighted in grams/liter (g L^−1^) as follows: 30.0 g glucose; 1.0 g peptone; 10.0 g beef extract; 5.0 g yeast extract; 0.2 g K_2_HPO_4_; 0.2 g triammonium citrate; 10.0 g sodium acetate trihydrate; and 2.0 g MgSO_4_.7H_2_O) [6]. Several 20 mL samples were taken from the bioreactor at specific time points (0, 12, 14, 16, 18, 20, 22, 24, 36, 40 and 44 h) during the fermentation period. The samples were centrifuged at 5000 rpm for 15 min and the supernatant were separated from the pellets in a separate tubes. Both tubes were kept in − 20 °C Toshiba freezer (Toshiba, Japan) until they were analyzed. Biomass weight, oligosaccharides concentration, and glucose concentration were determined as described in the following analytical procedures section.

### Analytical procedures

#### Biomass dry weight determination

Dry cell weight was determined in the 20 mL samples that were drawn from the bioreactor. The culture was centrifuged in an Eppendrof centrefuge (Eppendrof, Germany) at 5000 × g for 10 min. The pellet was then resuspended, washed, and centrifuged again using the same centrifugation parameters. Finally, the pellet was dried out overnight in a Sartorius dry oven at 60 °C until reaching a conistent weight and the weight was recorded for each sample [12].

#### Extraction and quantitative assay for LAB-oligosaccharides

At the end of the incubation period, oligosaccharides were extracted from the culture supernatant using the following procedure. Bacterial cells were pelleted by centrifugation at 10,000 rpm for 30 min at 4 °C, and the resulting culture clear supernatant was separated and filtered via a 0.22 µm membrane. To precipitate proteins, the extracellular mixture from the bacterial culture was initially treated with 10% trichloroacetic acid (1:1 v/v), after which the suspension was centrifuged at 10,000 rpm for 30 min at 4 °C to discard the pellet. The resulting clear supernatant underwent three successive cold ethanol precipitation steps (3:1 v/v (Ethanol: supernatant)). The oligosaccharides were then recovered by centrifugation at 10,000 rpm for 30 min at 4 °C, and the ethanol-soluble fraction was concentrated under reduced pressure at 40 °C via a rotary evaporator. The resulting purified LA-oligosaccharides fraction was kept at 4 °C until further analysis [7].

Quantitative determination of LAB-oligosaccharide concentration was carried out using the phenol–sulfuric acid colorimetric method for total carbohydrates quantification using glucose (Fisher Scientific, USA) as a standard [13]. 200 µL of 5% phenol was added to the 200 µL of soluble samples of taget oligosaccharides, followed by the addition of 1 mL of concentrated H_2_SO_4_. The reaction was boiled at 95 °C for 5.0 min, then allowed to cool down at room temperature. The absorbance of the resulted solution was measured spectrophotometrically at 490 nm against a blank made of all reagents without the soluble oligosaccharide sample added. The experiment was carried out in triplicate and the results were analyzed using ANOVA analysis using Graph Pad Prism 10 software.

#### Glucose determination

An enzymatic colorimetric kit (Diamond Diagnostics, Egypt) was used for measuring glucose concentration in the growth medium according to the manufacturer's instructions.

#### Insights into the structure of LAB-oligosaccharides after optimization.

The structure of the oligosaccharide mixture LAB-oligosaccharides was investigated to check for any sturactural changes, which may occur after scaling up the production, using NMR and mass-spectra analyses as follows:

##### NMR and mass analysis of oligosaccharide

1D and 2D ^1^H and ^13^C NMR spectra were acquired on a Bruker Avance 400 MHz NMR spectrometer (Karlsruhe, Germany) using D_2_O as the solvent. ^1^H and ^13^C resonance frequencies were 400 MHz and ~ 100 MHz, respectively.

##### Hydrolysis of LAB-oligosaccharides oligosaccharides for studying monomeric composition

The hydrolysis process was carried out using the following procedure [14]. A 200 mg portion of the investigated oligosaccharides was dissolved in 1N Fisher H_2_SO_4_ (10 mL) and heated in a boiling water bath (Memmert, USA) for 24 h under reflux. At the end of the hydrolysis time (24 h), the solution was cooled down to room temperature and neutralized using solid barium carbonate (Fisher Scientific, USA), then filtered using Whatman No. 1 filter paper (GE Heather, UK). The precipitate was washed twice with distilled water (5 mL), and the combined filtrate was concentrated to a 2 mL volume in a Sartorius oven (Sartorius, Germany) at 40 °C. The monomeric composition of the hydrolysate was determined by running paper chromatography for the uronic and non-uronic acid monomers alongside authentic monosaccharides (β-D-glucose, β-D-galactose, α-L-rhamnose, α-L-arabinose, D-xylose, D-galacturonic acid, and D-glucuronic acid) using the solvent system *n*-butanol/acetic acid/water (4:1:5) and visualized as brown spots by aniline/acid phthalate spray after heating it at 110 °C. The authentic sugars were obtained from Sigma Aldrich, except D-glucuronic acid, which was prepared from an authentic D-glucuronic acid lactone by dissolving 5 mg in 1N NaOH, and spontaneous hydrolysis of the ester linkage occurs to give D-glucuronic acid.

### Anticancer activity of LAB-oligosaccharides

The anticancer activities of LAB-oligosaccharides, extracted from bacterial culture samples drawn from the bioreactor over various time points during the 44 h fermentation period, were evaluated on CaCo-2 cell line via the MTS assay protocol. CaCo-2 cells were suspended in DMEM mamalian tissue culture medium (Lonza, Germany) to prepare a 6 × 10^4^ cells/mL cell suspended. A 100 µL of the prepared cell suspention was seeded into 96-well plates (100 µL per each well) and the plate was incubated at 37 °C in a humidified 5% CO₂ atmosphere for 24 h in an Eppendorf CO_2_ incubator (Eppendrof, Germany). Upon reaching semi-confluency, the exhausted DMEM medium was discarded and replaced with 100 µL of LAB-oligosaccharides samples (prepared in DMEM at a concentration of 10 mg/mL). For the negative control, 100 µL of culture medium alone was added. The plates were then incubated under the same conditions for an additional 24 h. Then, cell viability was assessed using the MTS assay kit (Promega, USA) according to the manufacturer's instructions. All experiments were repeated three times as biological replicates and each sample was repeated five times as technical replicates.

Cell morphology was evaluated after treatment using phase-contrast microscopy. At the end of the incubation period, treated cells were examined and compared with untreated controls to assess cell death and apoptosis using an EVOS M5000 inverted microscope (Thermo Fisher Scientific, USA).

### Quantitative real time PCR (qRT-PCR) gene expression analysis of apoptotosis-related genes

The molecular apoptotic mode of action of the LAB-oligosaccharides treatment occurred in CaCo-2 colon cancer cell line was investigated using qRT-PCR gene expression profile analysis of two apoptosis related genes (TGF and Survivin) and the house keeping gene GDPH. CaCo-2 cells were treated with 100 µL of of 10 mg mL^−1^ of LAB-oligosaccharide for 24 h. Both treated and nontreated control samples were scrabed from the plates and cells were collected by centrifugation using eppendrof centrifuge at 5000 rpm for 15 min and 4 C. The cell pellets were collected separately and RNA extraction was carried out using RNA extraction kit (Qiagen, Germany). Then, cDNA was synthesized using cDNA synthesis kit (Promega Corp., Madison, WI, USA) using Oligo dT_18_ primer according to the instructions of the manufacturer. Finally, qRT-PCR was carried out using TGF and survivin gene specific primers and GDPH as a house keeping gene for normlization, sequence of the primers are presented in Table 1. qRT-PCR reaction was completed using SYBR Green qRT-PCR Master Mix (Thermofisher, USA) and Light Cycler fluorimeter (Bio-Rad S1000 Tm thermal cycler). The PCR cycling program was as follows: 95 °C for 10 min, followed by 40 cycles of 95 °C for 30s, 58 °C for 30 s, and 72 °C for 45s, and finally 72 °C for 5 min. and gene expression analysis was done using CFX software (BioRad, USA).

**Table 1:** Gene specific primers for qRT-PCR reaction

Primer name	Sequence	Annelaing tem
Surv-F	5′-TGCCCCGACGTTGCC-3′	58 °C
Surv-R	5′-CAGTTCTTGAATGTAGAGATGCGGT-3′	58 °C
TGF-F	5′CAAGGGCTACCATGCCAACT-3′	58 °C
TGF-R	5′AGGGCCAGGACCTTGCTG-3’	58 °C
GAPDH-F:	5’-TTGGCTACAGCAACAGGGTG-3’	58 °C
GAPDH-R:	5’-GGGGAGATTCAGTGTGGTGG-3’	58 °C
Oligo dT_(18)_	5’-TTTTTTTTTTTTTTTTTT-3’	42 °C

### Evaluation of the efficacy of *L. acidophilus* pellets as a growth substrate for economically important enzyme-producing microorganisms

The potential use of *Lactobacillus acidophilus* pellets, the by-product remains after LAB-oligosaccharides extraction, as a substrate for cultivating economically valuable enzyme-producing bacteria was evaluated. Initially, several bacterial isolates were isolated from soil samples and screened for their ability to produce lipase and protease enzymes using phenol red-enriched and gelatin agar media, respectively, as described below.

#### Sample collection and preparation

Soil samples were collected from the shore of the eastern basin of Lake Marioutis (near the King Marioutis area), Alexandria, Egypt, a brackish lake in northern Egypt near the city of Alexandria, as a rich microbial source for isolating protease- and lipase-producing bacterial strains. The soil samples were subjected to serial dilution (10^–1^–10^–5^) to isolate enzyme-producing bacteria. All the dilutions were prepared in sterile Falcon tubes using sterile Phosphate Saline buffer (PPS) pH 7.0.

#### Preparation of the isolation media

##### Lipase detection medium

The medium used for detecting lipase production contained olive oil as the substrate and phenol red as the pH indicator[15]. The medium composition was; commercial olive oil (0.1% v/v), CaCl₂ (0.1% v/v), phenol red (0.01% w/v), and bacteriological agar (2% w/v). The medium was autoclaved at 121 °C and 15 psi for 15 min., then poured into sterile disposable Petri dishes and stored at 4 °C until usage. All chemicals were purchased from Fisher scientific company (Fisher, USA) except for bacteriological agar that was purchased from BioLabs (Biolabs, USA).

##### Protease detection medium

Gelatin agar medium was prepared for the isolation of protease-producing microorganisms; The medium was formulated by adding 8% gelatin to 100 mL of nutrient agar medium[16]. After thorough mixing, the medium was sterilized by autoclaving at 121 °C and 15 psi for 15 min., then the medium was cooled down to 45 °C, poured into sterile Petri dishes, and stored at 4 °C.

#### Isolation of lipase and protease-producing bacteria

To isolate lipase-producing bacterial strains, 50 µL of serially diluted soil sample suspension was aseptically spread out onto olive oil phenol red agar plates using a sterile glass spreader. After the inoculum had fully dried, the plates were incubated in an inverted position at 30 °C for 48 h. Colonies that caused a colour change to the medium (from red to yellow) were considered presumptive lipase-producers. These colonies were then aseptically transferred to fresh phenol red agar plates to confirm their purity.

For protease-producing bacteria isolates isolation, the serially diluted soil sample suspension was aseptically spread out onto the gelatin agar plates using a sterile glass spreader; after dryness, the plates were incubated at 30 °C for 48 h. The plates were then checked for colonies able to produce clear zones around their borders; such bacterial isolates were aseptically transferred to new gelatin agar plates as presumptive protease-producing isolates.

#### Evaluation of the ability of isolated bacteria to utilize lactic acid bacterial biomass

Lipase and protease detection media were prepared without olive oil and gelatin, respectively, and instead, the media were supplemented with 40 °C-dried lactic acid bacterial biomass at equivalent concentrations. The prepared media were inoculated with either protease- or lipase-producing bacterial isolates and incubated at 30 °C for 48 h. After incubation, bacterial growth on the lactic acid bacteria–modified media was assessed to evaluate their ability to utilize the lactic acid bacterial biomass as an alternative substrate (carbon or nitrogen sources). As the protease activity was qualitatively investigated, the lipase activity was investigated quantitatively in a triplicate experiment to evaluate the ability of the selected bacterial isolate to produce lipase enzyme using the lactic acid bacterial biomass as a source of nutrients. Bacterial culture inoculum (1%) of an overnight culture (OD _600nm_ 0.8–1.0) of the selected lipase-producing bacteria was inoculated into 300 mL of the lactic acid bacterial biomass suspension after sterilization. For comparison, in another conical flask, the same amount of the lactic acid bacterial biomass suspension was inoculated with the same bacterial inoculum size after amendment with 1% olive oil. The lipase production of both samples was determined using the spectrophotometry method after 3 days of incubation compared with the uninoculated sample of the fermentation pellet suspension.

#### Quantitative determination of lipase activity

The lipase activity at each mentioned optimization factor was determined via the quantification of the amount of the released fatty acids compared with the oleic acid standard curve according to [17] with a few modifications. After the inoculation of the selected lipase-producing bacteria to the standard production media (0.75% peptone, 1% olive oil, 0.3% yeast extract, 0.5% glucose, 0.05% CaCl_2_.2H_2_O, and 0.1% FeCl_3_.6H2O) followed by optimization according to the required study factor, 1 mL of the crude enzyme of each treatment was mixed with 2.5 mL olive oil emulsion (1:1 of olive oil: phosphate buffer (50 mM K_2_HPO_4_, 50 mM KH_2_PO_4_, at pH 7.0 and 0.02 mL of 20 mM CaCl_2_.2H_2_O)) followed by incubation at 30 °C and 150 rpm for 30 min. then, 1 mL of 6N HCl was added to each treatment in order to terminate the reaction. The free fatty acids were then extracted by vigorous mixing with 5 mL isooctane for 30 s. A total volume of 4 mL of the isooctane layer (the upper layer), which is supposed to have the free fatty acids, was transferred to a new test tube containing 1 mL of the copper reagent (5% copper (II) acetate-1-hydrate adjusted to pH 6.0 using pyridine solution). Both components were vortexed again for 30 s. The amount of the liberated fatty acids in the isooctane layer was determined using a spectrophotometer at 715 nm. One unit of lipase activity was defined as the release of one micromole of the free fatty acid in one minute.

#### Bacterial identification

The most potent bacterial strain/s that showed significant ability to use the lactic acid bacterial biomass by-product to produce valuable enzymes were selected and molecularly identified based on their 16S-rRNA gene sequencing. Clustal W online software was used for sequence alignment. Then, a Maximum Parsimony Phylogenetic tree was constructed to assess the relatedness between the bacterial strains using Mega 11 Evolutionary Analysis software (19). A bootstarp value of 1000 was used to ensure the reproducibility and confedence of the generated phylogenetic tree.

### Statistical analysis

All the experiments were carried out using three technical replicates except for anti cancer experiments where three biological replicates and five technical replicates were used. The standard deviation from the mean of the replicates was calculated and plotted on the curves where it is applicable. Two soft wares were used: Graph Pad Prism 10 and Sigma plot 9 statistical analysis softwares. For Gene sequence analysis, phylogenetic relatedness, phylogenetic tree calculation, and bootstrap value calculations; Mega 11 software was used.

## Results

### Production of LAB-oligosaccharides using batch fermentation

**Fig. 1:**
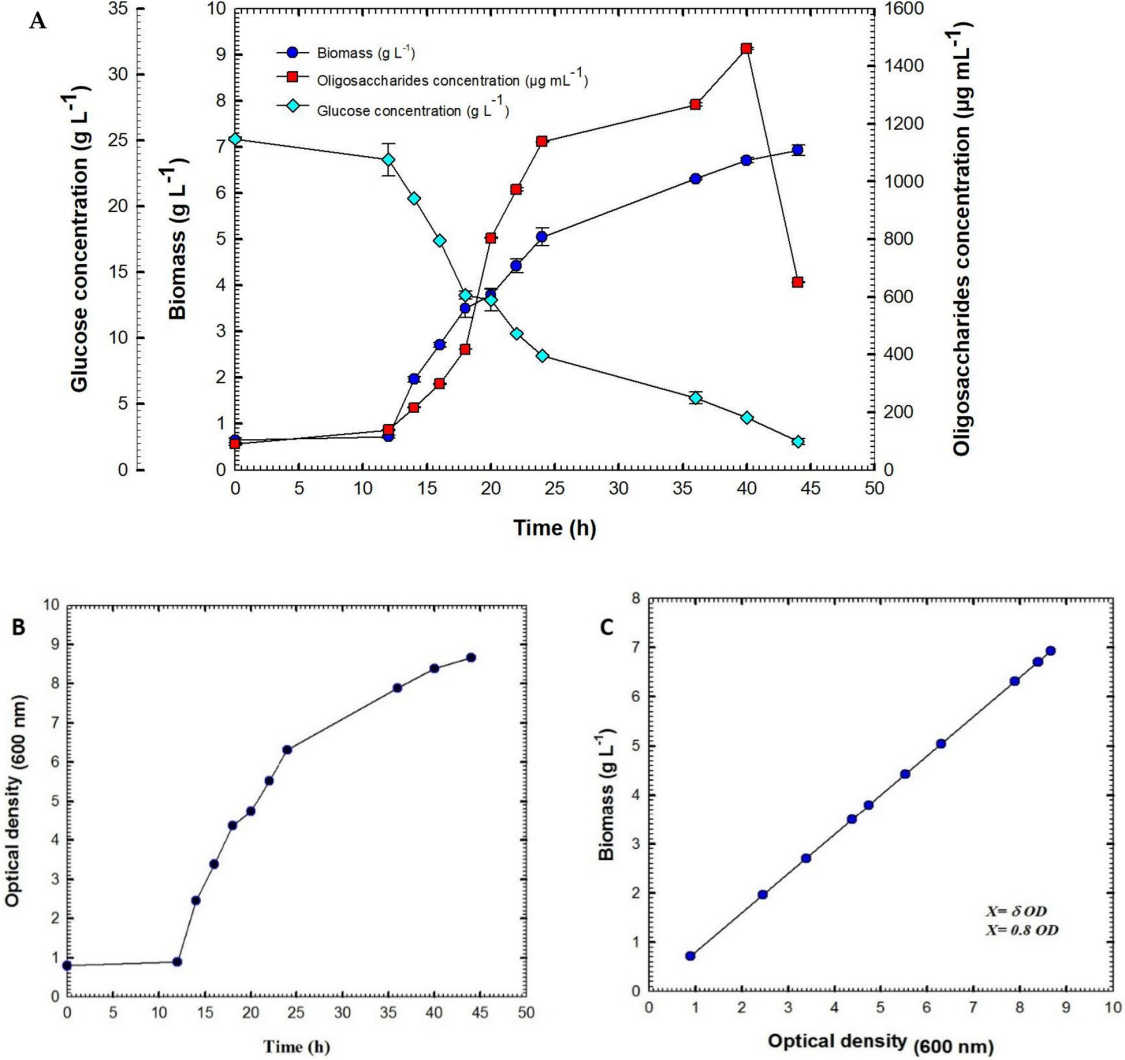
Depicts **A** Biomass, LAB-oligosaccharides concentration (ug mL^−1^), and glucose concentration (g L^−1^) in the culture broth as a function of time, **B** bacterial growth as optical density at 600 nm (OD_600nm_), and **C** correlation coeffecient shows the relatioship between OD_600nm_ and biomass concentration (g L^−1^) during batch fermentation of the *Lactobacillus acidophilus* bacterial strain 20,079. Error bars represents the standard deviation from the mean of three replicates grapg was generated using Sigma plot 9 statistical analysis software

Batch fermentation of *L*. *acidophilus* was started in a stirred tank bioreactor for the production of LAB-oligosaccharides with an optical density (O.D_600nm_) of 0.8 inoculum. Biomass and LAB-oligosaccharides, and glucose (the carbon source in the batch fermentation) concentrations were plotted against time (Fig. 1A). The maximum biomass recorded was 6.93 g L^−1^ at 44 h of fermentation time (Fig. 1A) with a maximum optical density (OD_600nm_) of 8.66 (Fig. 1B). Additionally, the correlation coefficient, which shows the relationship between optical density and biomass, was calculated and found to be equal to 0.8 (Fig. 1C).

**Fig. 2:**
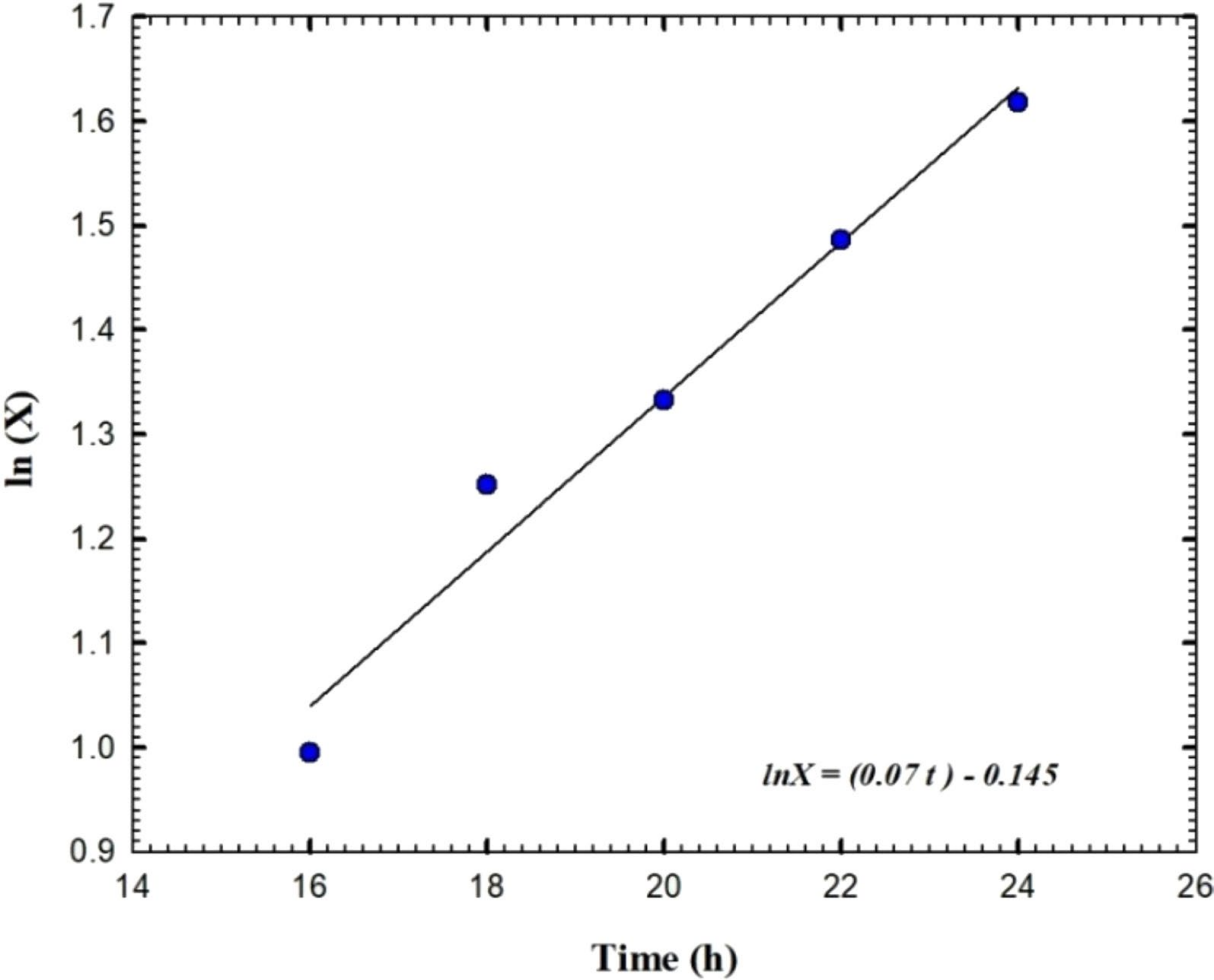
Natural log of the biomass concentration (X) as a function of time (h) during the exponential growth phase of the batch fermentation of *Lactobacillus acidophilus* 20,079 for the production of LAB-oligosaccharides

The result showed that the bacterial cell biomass increased exponentially over time with a constant specific growth rate (µ) of 0.07 h^−1^ that was calculated within the exponential phase of the culture growth using the logarithmic relationship presented in Fig. 2. In this figure, the natural logarithm (ln) of biomass increase was presented by a straight line on a semi-logarithm plot alongside time. The data indicated that the LAB-oligosaccharides concentration increased over time gradually and reached the maximum value of 1461.43 µg mL^−1^ at 40 h of the process. Meanwhile, glucose concentration decreased rapidly, reaching 10.34 g L^−1^ at 22 h, and reached 2.15 g L^−1^ at 44 h (Fig. 1A).

**Fig. 3:**
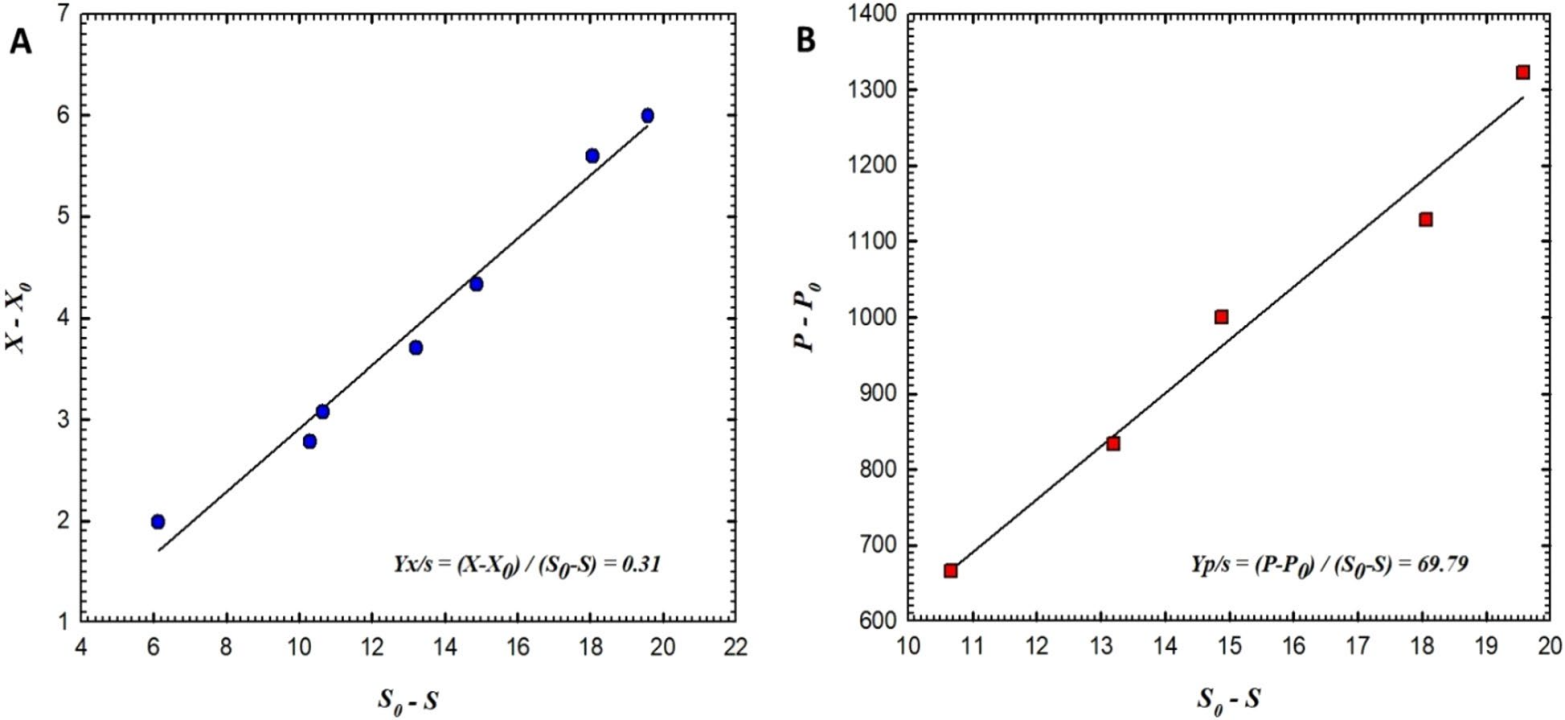
Yield coefficient of **A**: culture biomass (X) and **B**: LAB-oligosaccharides production (P) for culture growth of *Lactobacillus acidophilus* 20,079 on glucose. X_0_ and P_0_ represent the cell mass (g L^−1^) and LAB-oligosaccharides concentration (µg mL^−1^) in the broth at initial time (t_0_), respectively. While, X and P are the cell biomass concentration and LAB-oligosaccharides concentration in the broth culture at time t, respectively; S_0_, glucose concentration in the broth at initial time (t_0_) in g L^−1^ and S is the glucose concentration in the broth culture at time (t) in g L^−1^

**Fig. 4:**
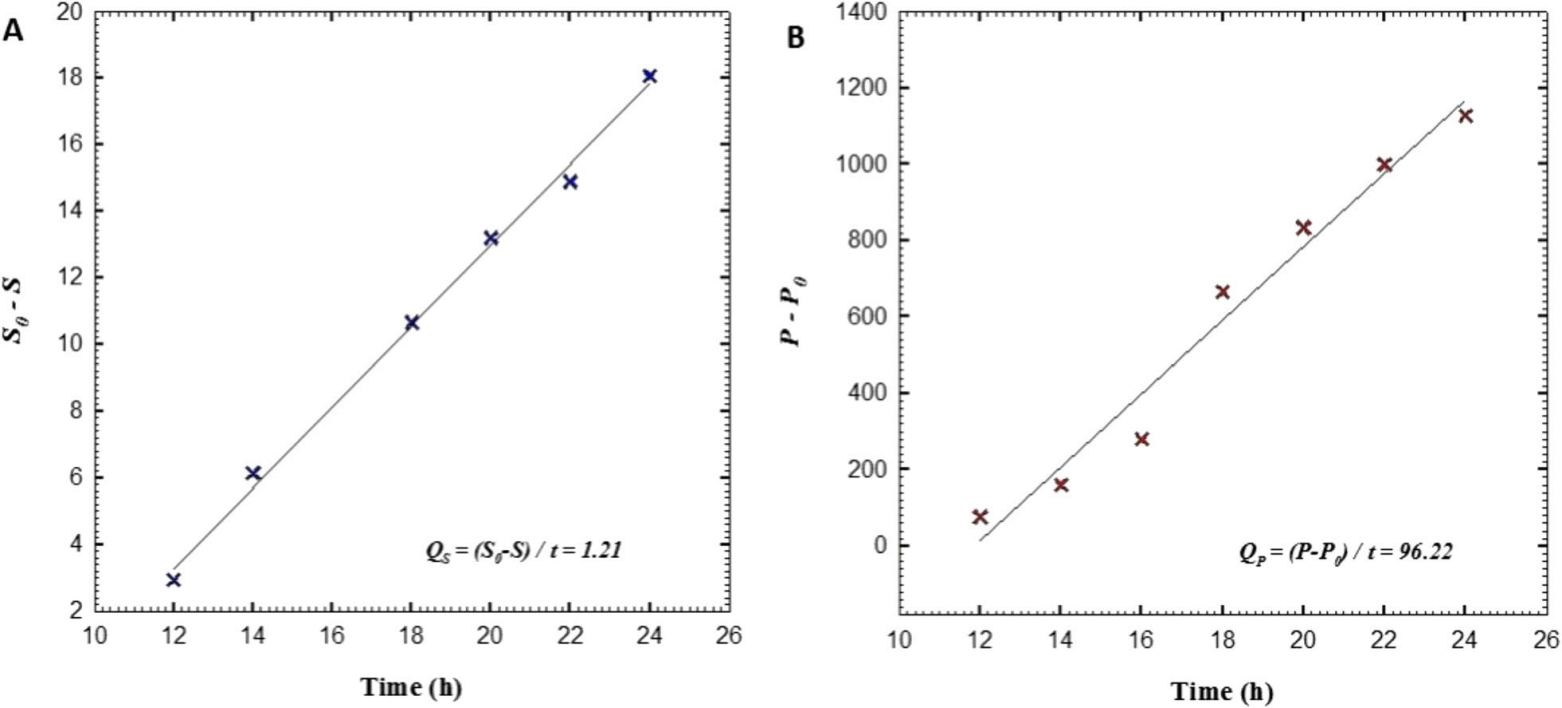
**A** is the Consumption rate of glucose as a carbon source and **B** is the production rate of LAB-oligosaccharides during the exponential growth phase of the culture of batch fermentation of *Lactobacillus acidophilus 20,079*. Where S_0_ is the glucose concentration at intial time (t_0_) and S is the glucose concentration at specific time point in g L^−1^. Meanwhile, P_0_ is the LAB-oligosaccharides concentration at the intial time (t_0_) in g L^−1^ and P is the concentration of LAB-oligosaccharides at a specific time point during the batch fermentation

One of the important factors estimated in the exponential phase of the bacterial cell growth is the yield coefficient (*Y*_*X/S*_), which represents the amount of biomass or LAB-oligosaccharides concentration against the amount of the consumed carbon source (glucose). In the current batch fermentation process, the yield coefficient of biomass (*Y*_*X/S*_) was estimated to be 0.31 g cells g^−1^ glucose (Fig. 3A), while the yield coefficient of LAB-oligosaccharides concentration (*Yp/S*) was 69.79 µg mL^−1^ g^−1^ glucose (Fig. 3B). Additional factors were calculated during the exponential phase to estimate the efficiency of bacterial growth and the production of LAB-oligosaccharides through the batch fermentation process in the growth medium, including the consumption rate of the carbon source (glucose) (*Q*_*S*_) and the production rate of LAB-oligosaccharides (*Qp*). The consumption rate of glucose was 1.21 g L^−1^ h^−1^ with specific substrate consumption rate (*q*_*S*_) of 2.8 g glucose/g biomass/h (Fig. 4A), whereas the production rate of LAB-oligosaccharides reached 96.22 µg mL^−1^ h^−1^ with specific product formation rate (*q*_*p*_) of 174.98 µg mL^−1^ g^−1^ biomass h^−1^ (Fig. 4B).

**Fig. 5:**
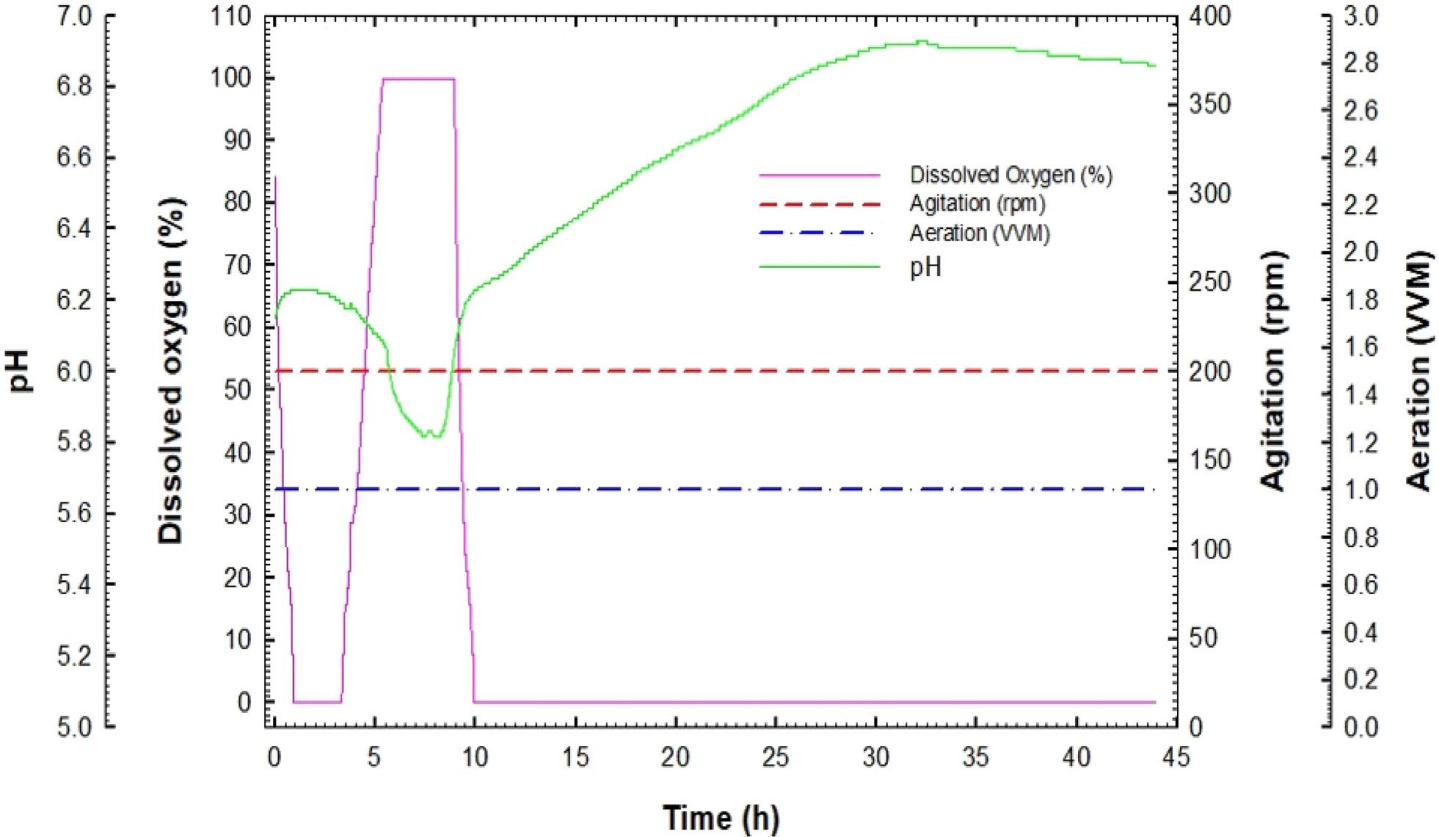
Bioreactor Online Data showing the values of dissolved oxygen (%), agitation speed (rpm), aeration rate Volume air/ Volume medium/ Minute (VVM), and pH plotted against time during the uncontrolled batch fermentation process of *Lactobacillus acidophilus* strain 20,079 using the optimized medium. The figure was generated using onlinedata of the bioreactor

Dissolved oxygen is another important factor in bacterial growth and the production of LAB-oligosaccharides from *L*. *acidophilus* in the bioreactor, which can be controlled by agitation speed and aeration rate. During the current study, batch fermentation of *L*. *acidophilus* started with an agitation speed of 200 rpm and an aeration rate of 1 VVM, resulting in a higher percentage of dissolved oxygen equal to 84.2%. The dissolved oxygen percentage decreased dramatically during the first hour of the batch fermentation process to reach 0%, followed by increase starting from 3 h post-inoculation to reach 100% after 5.5 h. The dissolved oxygen percentage continued at 0% until the end of the batch fermentation process. A low reduction in pH (5.82) was detected from the beginning of the fermentation process until 8 h post-inoculation, followed by a gradual increase in pH to reach the maximum value of 6.93 at 32 h (Fig. 5).

### Insights into the identification of the produced LAB-oligosaccharides

#### Monomeric composition

Paper chromatography of the complete hydrolysis mixture alongside the authentic sugars revealed that the monomeric sugars are β-D-galactose, α-L-rhamnose and D-glucuronic acid.

#### NMR investigation

**Fig. 6:**
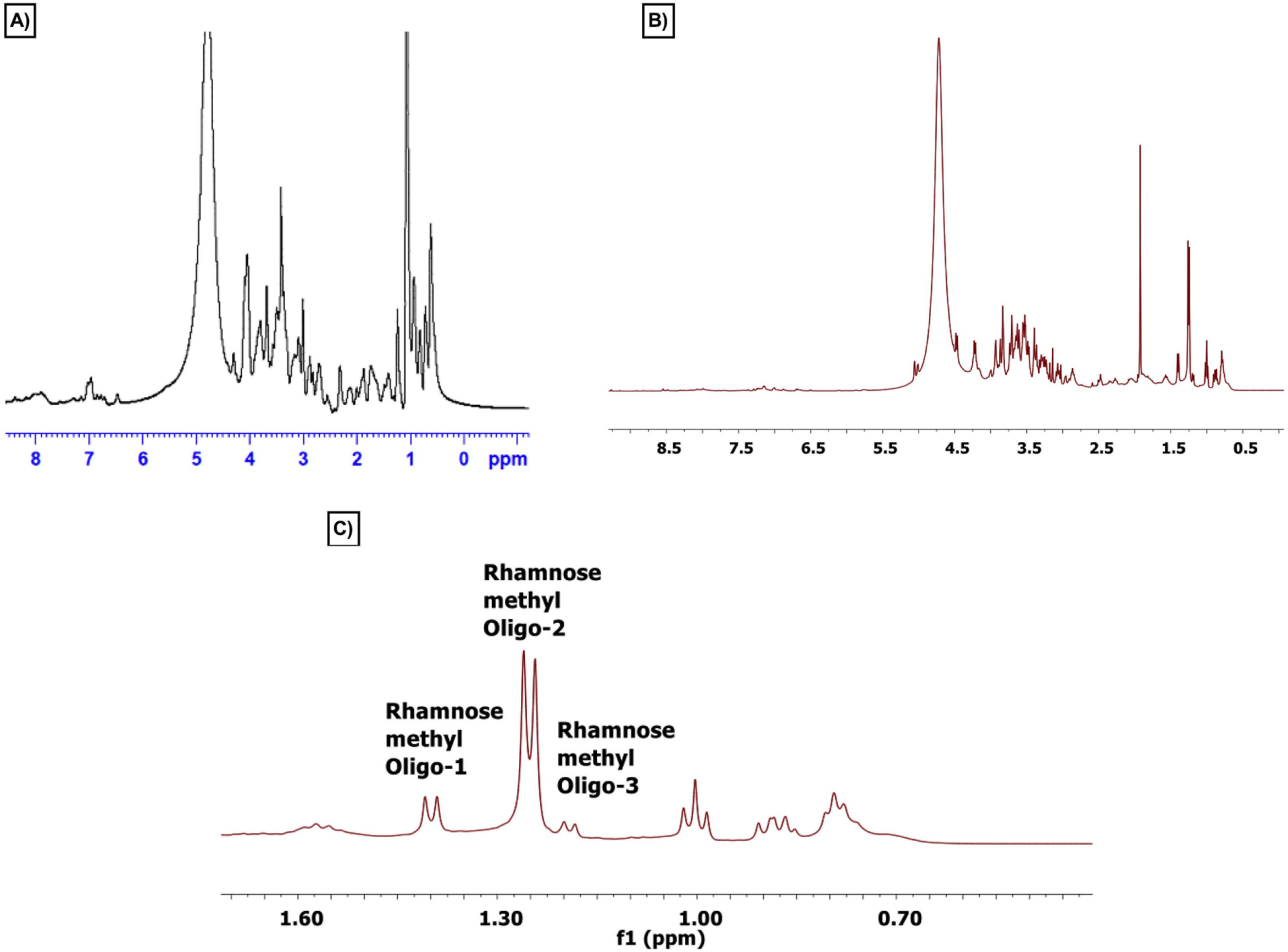
^1^H NMR spectrum of LAB-oligosaccharides product in D_2_O. **A** with impurities before extraction; **B** LAB-oligosaccharides after extraction with ethyl acetate; **C** zoom screen of the ^1^H NMR spectrum between 0.5 and 1.5 ppm

The ^1^H NMR spectrum of the oligosaccharide product showed impurities (Fig. 6A, Additional file 1: Figure S1). Thus, the oligosaccharide product was redissolved in distilled water and extracted with ethyl acetate to reduce the accompanied impurities. The aqueous part was lyophilized and re-analyzed by ^1^H NMR.

The ^1^H NMR spectrum (Fig. 6B) indicated the presence of 3 oligosaccharide structures (Oligo-1, Oligo-2, and Oligo-3) indicated by the presence of three doublet signals for rhamnose sugars at 1.4, 1.25 and 1.2 ppm (Fig. 6C, Additional file 7: Tables S1 and S2), for which the integration indicated that the percentages of these oligosaccharides are 15.38%, 76.93%, and 7.69%, respectively.

**Fig. 7:**
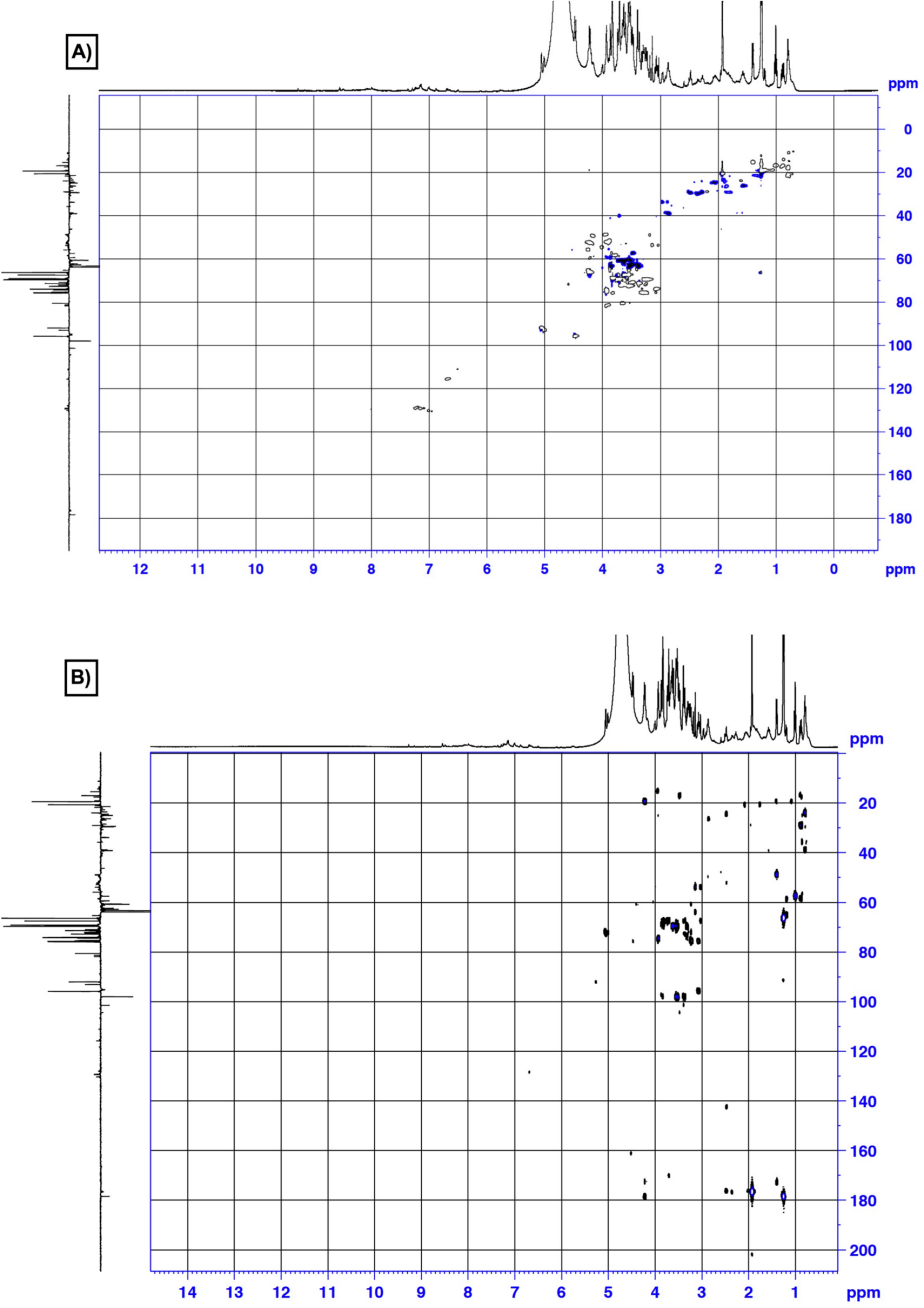
**A** HSQC spectrum and **B** HMBC spectrum of the extracted LAB-oligosaccharides in a batch fermentation in optimized medium

The HSQC spectrum (Fig. 7A, Additional file 7: Table S2, Additional file 3: Figures S3 and Additional file 4: Figure S4) indicated the presence of quaternary carbons at 178.4, 176.5, and 176.1 ppm, which could be attributed to the acid carbonyls in the glucuronic sugars. The signals at 176.5 and 176.1 ppm showed correlations to a singlet signal at 1.92 ppm in the HMBC spectrum (Fig. 7B, Additional file 5: Figure S5, Additional file 6: Figure S6), which revealed the presence of glucuronic acid as two methyl esters and one free acid in the 3 oligo structures. As the integration of the singlet signal at 1.92 ppm compared to the methyl group of the rhamnose was 1 to 3, the minor oligo-1 and oligo-3 were considered the ester forms, while the major oligo-1 was considered the free form of glucuronic acid. The carbon signals at 93.2 and 91.9 ppm for C-H carbons were attributed to the anomeric carbon and C-2 position of the glucuronic acid parts, indicating that glucuronic acid sugars could be involved in the glycosidic linkage only by the C-2 position. The carbon signals at 98.1 and 101.5 ppm for the anomeric carbon of the galactose sugars were found to be quaternary, which implied that the C-1 carbon of the galactose part is involved in a C–C bond, suggesting a C-glycoside linkage. Additionally, the carbon signals at 60.6, 63.4, and 63.8 ppm were assigned for the C-6 of three galactose sugars, while the signal at 69.4 ppm was attributed to a deshielded C-4 of a galactose sugar, which indicates that, in the two oligo structures, C-6 was involved in a glycosidic linkage, while in the third oligo, C-4 was the linkage site. In addition, the carbon signal at 95.8 ppm, assigned for the anomeric carbon of rhamnose monomer, implied that C-1 of rhamnose was not involved in the glycosidic linkage, while the carbon signal at 80.6 ppm indicates that C-3 of rhamnose was the glycosidic linkage site. These data suggest that the order of the sugars in the three oligos is glucuronic acid/ester (C-2) is linked to galactose through its C-6 or C-4, and the C-1 of galactose sugar is linked to C-3 of rhamnose sugar through a C–C bond.

### Anticancer activities of the collected bioreactor fractions

**Fig. 8:**
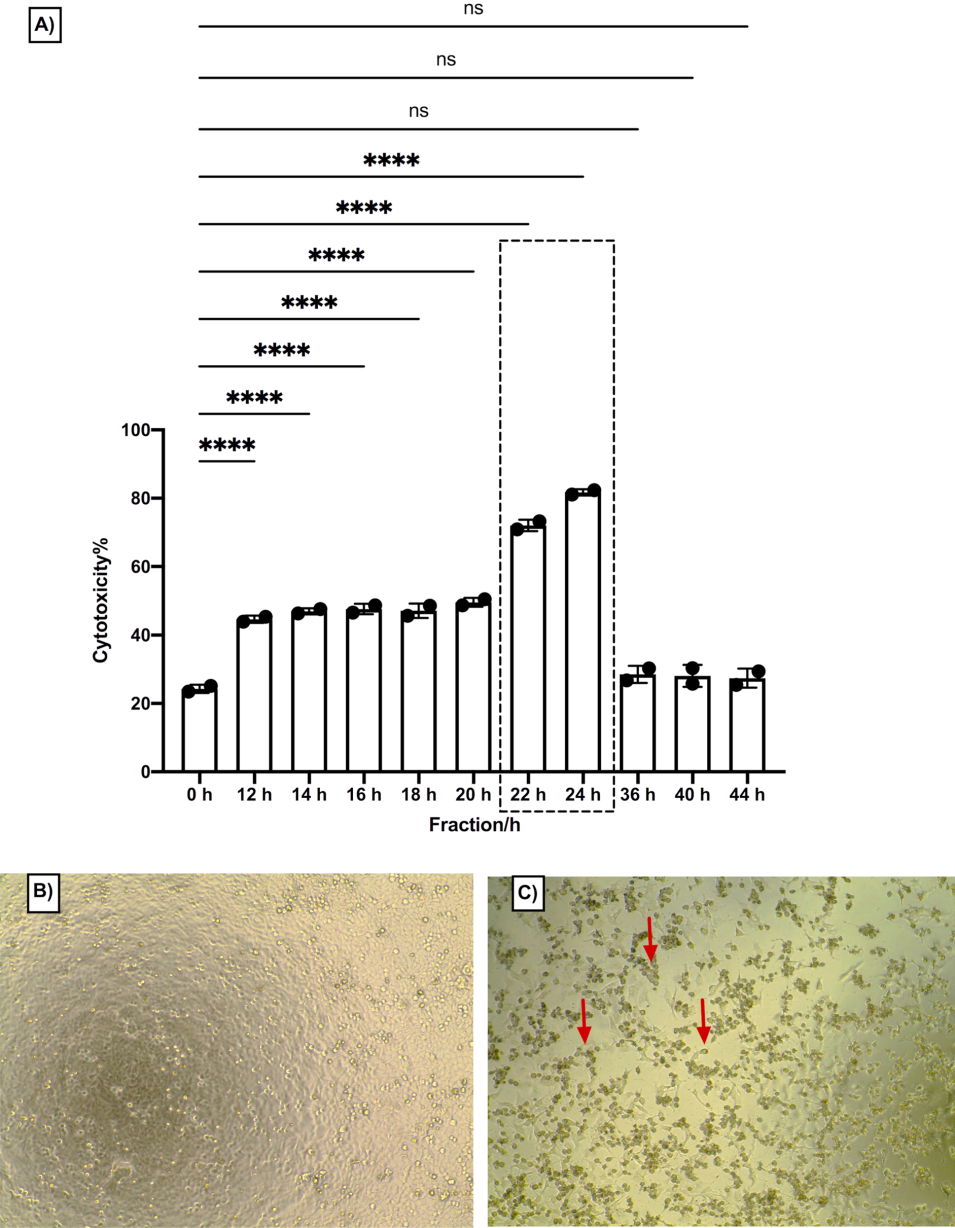
Anticancer activities of the collected bioreactor fractions on CaCo-2 cell line. The anticancer effects of the collected fractions were evaluated on CaCo-2 cell line using MTS assay protocol after 24 h (8A), figure was generated using GraphPad Prism 10 software. Phase-contrast microscopy revealed that the most promising effect was obtained from the bioreactor fraction collected 24 h post inoculation, where 8B: untreated control cells (confluent cells) and (8C): LAB-oligosaccharides-treated cells, which induced apoptotic cell formation (rounded cells, red arrows) after 24 h of treatment and inhibited cellular growth comparing to the control non-treated cells

The anticancer activities of the collected fractions from the bioreactor, which were collected at the following specific intervals (0, 12, 14, 16, 18, 20, 22, 24, 36, 40 and 44 h) were quantified using MTS assay protocol. The data obtained (Fig. 8A) indicated that the maximum anticancer activities were observed after 22 and 24 h with inhibition percentages of 72.0 and 81.7, respectively. The results showed the induction of apoptotic cells after 24 h of treatment (Fig. 8B): untreated control and (8C): LAB-oligosaccharides treated cells).

### Gene expression profile of TGF and survivine genes

**Fig. 9:**
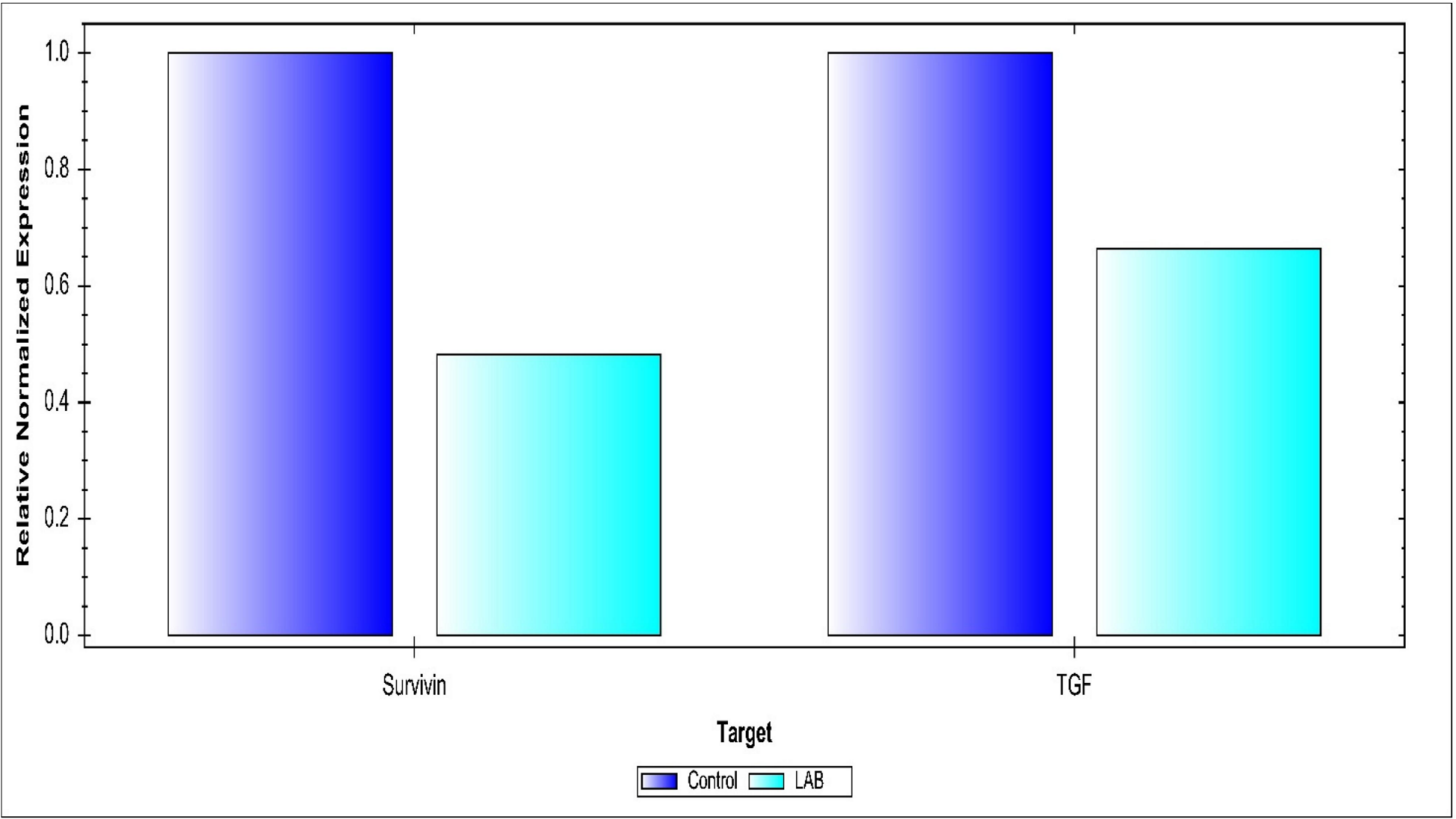
Relative gene expression of Survivin and TGF genes (green treated cell line) normalized using GDPH as a house gene (blue control columns). Relative gene expression analysis was performed usin CFX software (BioRad, USA)

To investigate the potential mode of action of the produced LAB-oligosaccharides as a potential anticancer agent, we studied the gene expression profile of two apoptotic-related genes in CaCo-2 cell line as follows: Treatment: cell line treated with 100 µl of the soluble LAB-oligosaccharides Control: untreated cell line. Gene expression level was normalized and relative gen expression was calculated and presented in Fig. 9. The results indicated that both TGF and survivin genes were down regulated in the treated cell line compared to the untreated control.

### Evaluation the the efficacy of *L. acidophilus* pellets as a substrate for the production of economic enzymes using enzymes-producing bacterial isolates

#### Isolation of lipase-producing bacteria

**Fig. 10:**
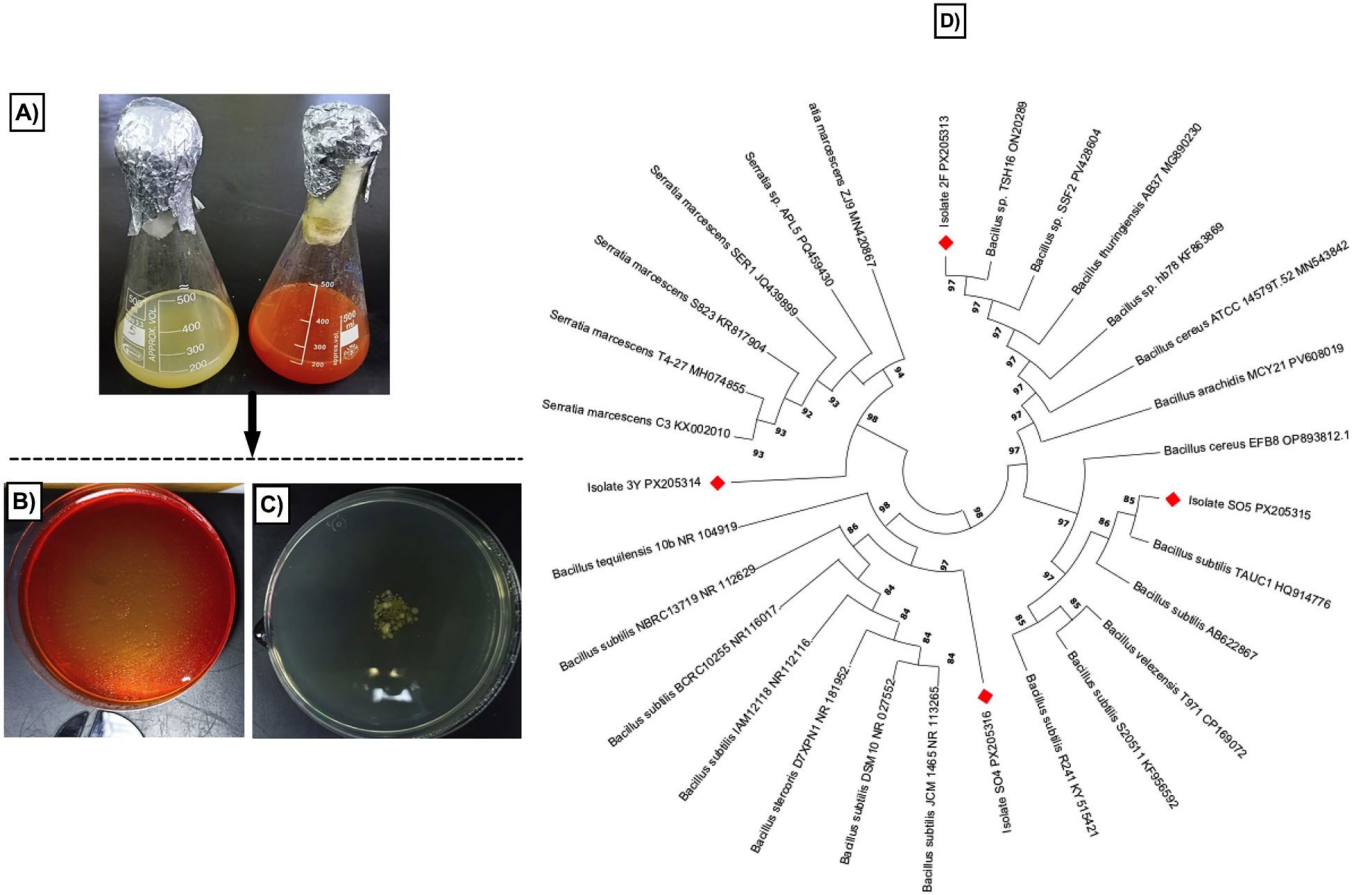
represents the isolation steps of lipase- and protease-producing bacterial isolates. **A** Phenol-red agar medium was prepared for the isolation of lipase-producing bacteria (red conical flask) and gelatin agar medium for the protease-producing bacteria (colorless conical flask). The serially diluted soil samples were placed on the phenol-red agar plates and gelatin agar plates. Six bacterial isolates were purified; four out of the six isolates were positive for lipase (**B**) and protease (**C**). Finally, Phylogenetic analysis of the 16S rRNA gene sequences was completed using Mega11 Evolutionary-analysis software [18] to generate a phylogenetic tree using the Maximum Parsimony method (**D**). The four bacterial isolates characterized in this study are labeled in red, and the remaining sequences were collected from the GenBank database. Four phylogenetic clades were generated, where each clade combined one of the isolated bacterial isolates along with the most similar reference 16S rRNA gene sequences. The numbers shown on each branch show the bootstrap value as a percentage of the generated tree, 1000 bootstrap test were performed to ensure branches accuracy

After spreading the serially diluted soil samples on phenol red agar plates, colonies that produced a yellow halo around the colonies in the medium were considered potential lipase producers. A total of five lipase-producing bacterial isolates were detected, purified, and re-inoculated onto fresh phenol red agar plates to confirm their ability to induce the colour change (Fig. 10A).

#### Isolation of protease-producing bacteria

The serially diluted soil samples were spread onto gelatin agar plates and incubated to isolate protease-producing bacteria. Six bacterial isolates formed clear zones around their colonies (five of the isolates were also positive for lipase production (Fig. 10B), as previously described in Sect. "Isolation of lipase-producing bacteria".), indicating proteolytic activity resulting from the complete degradation of gelatin (Fig. 10C) [19]. These positive isolates were aseptically transferred onto fresh gelatin agar plates to confirm their protease production capability (Fig. 10B and C). The ability of the 4 bacterial isolates to grow and utilize *L. acidophilus* biomass as a substrate for the production of lipase and protease enzymes was tested. Three out of six bacterial strains showed the ability to grow on media supplemented with LAB biomass and produced both lipase and protease enzymes. The selected bacterial isolates were identified molecularly based on their 16S rRNA gene sequence. 16S rRNA gene sequences were analyzed and submitted to the GenBank database, where accession numbers were assigned. Phylogenetic analysis of the 16S rRNA gene sequences revealed that the isolates were classified into 4 different phylogenetic clades, which are distinctly separated from each other (Fig. 10D). Isolate 3Y (accession number PX205314) was grouped with *S. marcescense* strains in a monophyletic clade. Meanwhile, 2F isolate (accession number PX205313) was grouped in a separate clade with strains belonging to different species of the *genus Bacillus*. Both isolates SO4 ( accession number PX205316) and SO5 (accession number PX205316) showed close similarity to *B. subtillus,* and each isolate was separated in an independent phylogenetic clade (Fig. 10D).

#### Determination of the lipase activity in lactic acid bacterial biomass suspensions

**Fig. 11:**
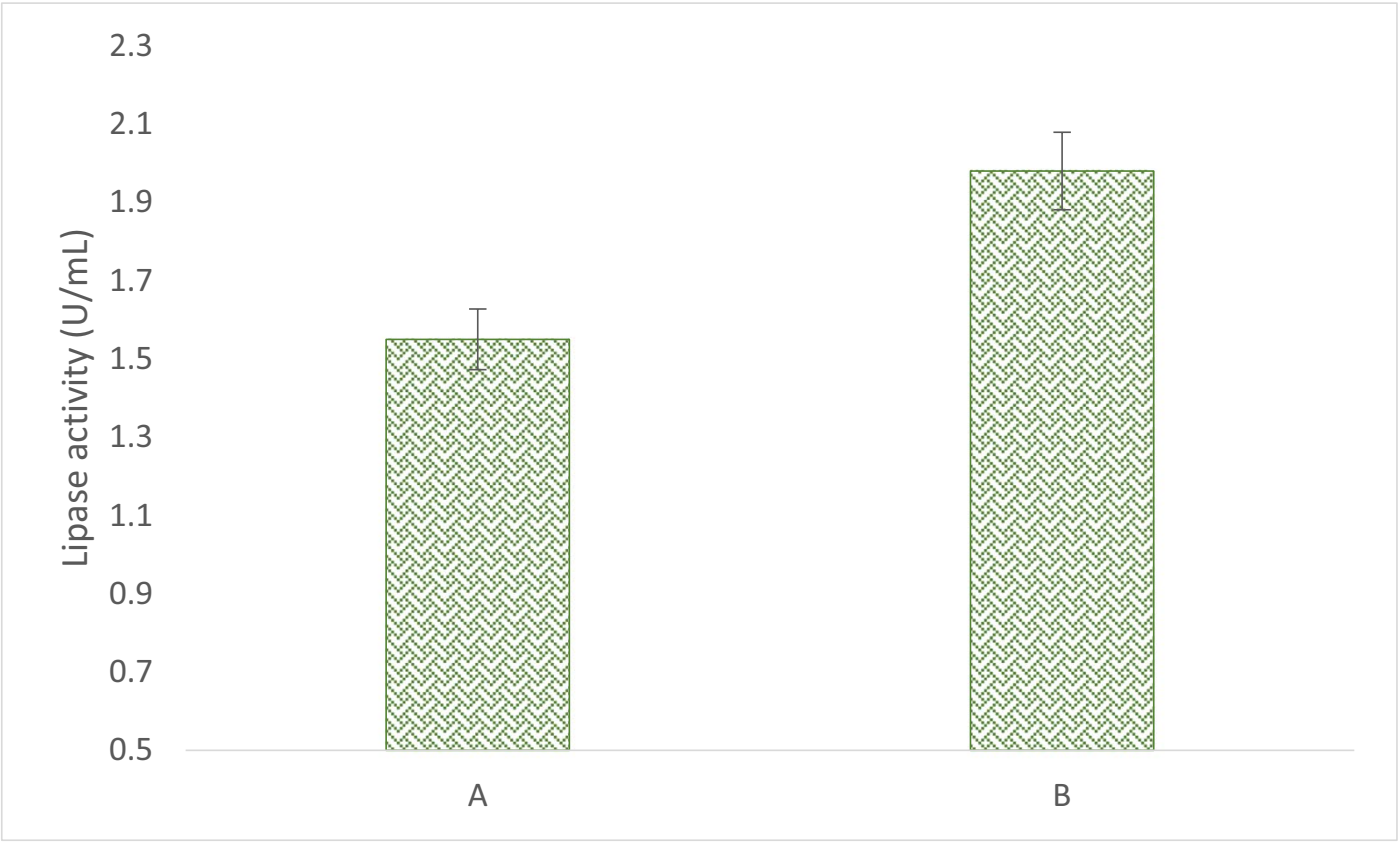
Investigation of the ability of *B. subtilis* PX205316 bacterial strain to produce lipase enzymes in the lactic acid bacterial biomass suspension. **A** Lactic acid bacterial biomass suspension inoculated with the *B. subtilis* PX205316 strain, and **B** Lactic acid bacterial biomass suspension inoculated with the *B. subtilis* PX205316 strain and amended with 1% olive oil. Error bars represent the standard deviation of the three biological samples

The ability of *B. subtilis* PX205316 bacterial strain to produce lipase enzyme using the lactic acid bacterial biomass suspension as the production media was investigated in the presence and absence of 1% olive oil. As shown in Fig. 11**,** both of the lactic acid bacterial biomass suspensions (amended with and without olive oil) can be used as a production medium for bacterial lipase. However, the presence of olive oil increased the enzyme production. The obtained data showed that the lipase activity was recorded as 1.5 U mL^−1^ in the absence of olive oil, which increased to 1.98 U mL^−1^ when olive oil was added to the fermentation suspension. These results conclude the successful use of the fermentation pellet suspension alone for the production of bacterial lipase enzyme with the ability to enhance the enzyme production through the addition of oil substrate to this suspension media.

## Discussion

Exo-poly-/oligo-saccharides derived from bacterial sources have been used in therapeutic and industrial applications without any recorded adverse effects (4, 5). In our previous study, we introduced LA-EPS-20079, oligosaccharides derived from *Lactobacillus acidophilus* DSMZ 20079, as a potential anti-colon cancer agent with the ability to inhibit the in vitro growth of the CaCo-2 cell line and downregulate the expression of some oncolytic genes [6]. Despite the promising activity of this exooligosaccharide against colon cancer cell lines, its low production efficiency and the high cost of the lactic acid specific medium that is used for the growth of lactic acid bacteria remains major barriers to large-scale production of these important metabolites. Optimizing the production medium is a critical step in the fermentation biotechnology area [20]. In our previous work, we successfully optimized the production of LA-EPS-20079 using a cost-effective new medium [6], which was employed in the current study to scale-up the production of the same bacterial strain on large scale. Optimization of the medium composition, growth conditions, cultivation mode, and bioreactor design are essential factors to obtain high yield with the desired purity and physicochemical characteristics (19). During the scale-up process, most laboratory and industrial scale fermentations are carried out in stirred-tank reactors (STRs) [21]. When STRs are compared to a shake flask, a stirred tank bioreactor offers the ideal conditions for bacterial growth, including temperature, pH, agitation, and aeration with consistency, which allow the cells to produce more product and other secondary metabolites in homogenous conditions [22]. Therefore, in the present study, a ten-liter bench-top stirred tank bioreactor, which is operated in batch fermentation mode, was used for the cultivation of *L. acidophilus* to produce LAB-oligosaccharides mixture, employing our previously optimized protocols for the biomass and oligosaccharides production using statistical design methodologies [6]. The result showed that total biomass of *L. acidophilus* reached a maximum of 6.93 g L^−1^ after 44 h of growth with a constant specific growth rate of 0.07 h^−1^. In a related study, a maximal biomass of 5.13 g L^−1^ was achieved using *L. plantarum* ATCC 8014 for the optimization of EPS production system (25).

In the current batch fermentation process, we used 30 g L^−1^ of glucose (as an optimum carbon source) to initiate *L. acidophilus* growth, which promptly declined through the exponential growth phase of the bacterial growth, resulting in a consumption rate of 1.21 g L^−1^ h^−1^. While, the oligosaccharides concentration increased gradually and reached the maximum value at 40 h with a production rate of 96.22 µg mL^−1^ h^−1^. This finding confirmed the importance of glucose as an optimum carbon source for oligosaccharides production from *L. acidophilus*, which was proved in another study using mesophilic *Lactobacillus acidophilus* [23].

The availability of nutrients and dissolved oxygen are two critical factors influencing polysaccharides production, and both are strongly affected by the mixing speed and aeration rate. In some microorganisms, vigorous agitation and/or high aeration rates can enhance polysaccharide synthesis by improving oxygen transfer and nutrient distribution [21]. However, excessive agitation or aeration can damage cells or alter the physicochemical characteristics of the produced polysaccharides [21]. Conversely, low agitation results in poor mixing and insufficient mass transfer of oxygen and nutrients. Therefore, an optimal balance between agitation and aeration is essential to maximize polysaccharides yield while maintaining its structural and functional integrity [21]. In the present study, a mixing speed of 200 rpm and an aeration rate of 1 VVM were used during the batch fermentation of *L. acidophilus*. A rapid decline in dissolved oxygen (DO) was observed after 9 h of cultivation, reaching 0% at approximately 10 h. Interestingly, the production of oligosaccharides began concurrently with the drop in DO, and the dissolved oxygen level remained at 0% for the remainder of the fermentation process. This pattern suggests that oxygen limitation may play a role in triggering polysaccaride synthesis. Consistent with our findings, Freitas et al. [21] found that low levels of DO_2_ promote exopolysaccharides production. Another important variables for the production of bacterial exopolysaccharides is the pH of the fermentation medium [24, 25].

The avilable studies for the production of oligosaccharides from lactic acid bacteria are limited but a few studies explained that oligosaccharides yields are varied among lactic acid bacterial strains, ranging from approximately 174.9–295.4 mg L⁻^1^ for most strains reported in Khalil et al. [5]. The study indicated that, *Streptococcus thermophilus* DSM 24731 produced 240.0 ± 3.3 mg L⁻^1^, while *Lactobacillus lactis* ssp. *cremoris* DSM 20069 T produced a lower yield of 189.72 ± 3.4 mg L⁻^1^. Higher production was also observed in *Lactobacillus casei* DSM 27537 with 245.6 ± 3.2 mg L⁻^1^. Furthermore, the same study explained that *Lactobacillus delbrueckii* subspecies, DSM 20080 produced 222.4 ± 7.1 mg L⁻^1^, whereas DSM 20081 T showed a higher yield of 295.4 ± 9.7 mg L⁻^1^, representing one of the highest values in this dataset. *Lactobacillus fermentum* DSM 20049 also demonstrated relatively high production at 255.3 ± 4.3 mg L⁻^1^. In a related study[6], *Lactobacillus acidophilus* DSM 20079 T and *Lactobacillus rhamnosus* DSM 20021 produced moderate yields with values of 213.4 ± 4.2 mg L⁻^1^ and 214.5 ± 4.1 mg L⁻^1^, respectively. *Lactobacillus plantarum* ssp. *plantarum* DSM 20174 showed a lower yield of 174.9 ± 5.2 mg L⁻^1^. Also, a higher production was also recorded for *Bifidobacterium longum* ssp. *longum* DSM 200707, reaching 260.3 ± 7.1 mg L⁻^1^. In optimized conditions, *Lactobacillus acidophilus* DSM 20079 exhibited a markedly enhanced yield of 795 mg L⁻^1^ [6], while Sheng et al. [26], indicated that *Lactobacillus pantheris* TCP102 produced about 525 mg L⁻^1^.

At the beginning of fermentation, the reduction in pH (from 6.2 to 5.82) is typically associated with the production of organic acids (such as lactic, acetic, or other short-chain acids) resulting from carbohydrate metabolism during the exponential growth phase. After several hours of cultivation, when readily metabolizable carbon sources become limited, many bacteria shift their metabolism toward the utilization of nitrogenous and proteinaceous compounds. This metabolic shift leads to the deamination of amino acids and the release of ammonia (NH₃/NH₄⁺) and other alkaline metabolites into the medium, which explains the gradual increase in pH observed after the 8 h. This behavior has been widely reported in fermentation studies and microbial physiology literature [18]. The accumulation of these basic compounds cause a slight increase in the culture pH. Similar observations were reported by [27], who documented a decline in the culture broth pH of *L. plantarum* ATCC 8014 from 6.0 to 4.2. Consistent results were also described by Goktas et al. [28], who observed a comparable reduction in pH during the cultivation of *L. plantarum* ATCC 8014. In another study, a structurally uniform pentasaccharide from *L. acidophilus* 20,079 was explained, which is composed of α-linked D-glucuronic acid, D-glucose, and L-fucose, the three structurally distinct oligosaccharides identified in our study (Oligo-1, Oligo-2, Oligo-3) reveal a markedly different biosynthetic outcome [4]. The presence of β-D-galactose, α-L-rhamnose, variable glucuronic acid esterification, and both C-glycosidic and O-glycosidic linkages suggests that the fermentation environment used in our work characterized by oxygen limitation, dynamic pH shifts, and defined agitation/aeration may have redirected sugar precursor availability and glycosyltransferase activity. Such media-driven structural modifications are consistent with previous reports demonstrating that environmental factors, particularly oxygen tension and pH, can alter exopolysaccharides monomer composition and linkage patterns in lactic acid bacteria and other microorganisms [29, 30]. Therefore, the emergence of three oligosaccharide variants in LA-oligosaccharides further supports the concept that media optimization not only influences exopolysaccharides yield but can fundamentally reshape its structural architecture.

Concerning the anticancer activities of the produced LAB-oligosaccharides, the temporal analysis of in-vitro anticancer activity revealed a critical dependency on fermentation time, with a definitive peak in efficacy observed between 22 and 24 h. The maximum inhibition of colon cancer cell line viability was recorded at these timepoints, with inhibatory percentage values rising to 72.01% and 81.7%, respectively. This pattern aligns with several established literature, where the production of bioactive metabolites, particularly exopolysaccharides from lactobacilli, is often growth-phase dependent, reaching a maximum effect during the late-exponential or stationary phase [31, 32]. The significant increase in activity, closing at 24 h, strongly suggests that the synthesis and secretion of LAB-oligosaccharides reach an optimal concentration window during this critical phase of *L. acidophilus* growth. Furthermore, the mode of this potent anticancer activity was explicitly identified as the induction of apoptosis. This is a crucial finding, as it is a recognized and desirable mechanism for chemopreventive agents derived from microbial sources, avoiding the inflammatory responses associated with necrotic cell death [33]. The efficacy of our compound (81.7% inhibition) compares favourably with other reported microbial bioactive compounds; for instance, EPS from traditional fermented foods have demonstrated a potent, time-dependent antiproliferative effect on cancer cells [34]. As a research study indicated that the treatment of HT-29 colon cancer cells with EPS (40 mg mL^−1^) from *L. paracasei* and *L. brevis* significantly increased cell death, with efficacy rising from 36 to 80% and from 40 to 90% between 24 and 72 h, respectively [35]. Collectively, these findings establish the critical time point for harvesting in the pilot-scale process, which is essential for achieving the highest yield of a bioactive metabolite with significant anti-colon cancer activity mediated through the apoptosis pathway.

In a comparison with the previously reported in-vitro anticancer profile of LA-EPS-20079 against HepG-2 and MCF-7 cell lines [4], which observed notable cytotoxicity of LA-EPS-20079 against the two cell lines, our LAB-oligosaccharides exhibited a similarly potent yet temporally defined activity, peaking between 22 and 24 h with up to 81.7% inhibition of colon cancer cell viability. This heightened efficacy coincides with the structural heterogeneity generated under our optimized fermentation conditions, suggesting that variations in esterification and linkage patterns may enhance apoptotic induction relative to the uniform pentasaccharide previously described. Unlike the broader spectrum cytotoxicity reported, the present work demonstrates a pronounced and mechanistically confirmed pro-apoptotic effect specifically against colon cancer cells. qRT-PCR gene expression analysis showed a downregulation effect of the *TGF* and *Survivin* genes. The two genes are apoptotic-related genes and was used to confirm te apoptotic effect of different substrates on different types of cancer cell lines [36]. Abo Zaid et al. (2023) showed that the effect of LA-EPS-20079 oligosaccharide of *L. acidophilus* was down regulation of *Bcl2* and *Survivin* gene expression levels and confirmed the apopotic effect upon treating CaCo-2 cell lines with the extracted oligosaccharides (6). Also, Taguar et al. [36] confirmed the apoptotic induction of compound 14a against CaCo-2 cell line using four apoptotic genes (*Bcl2, Bcl-xl, TGF,* and *Survivin*). They revealed that the exerted apoptosis occurred through a significant reduction in Bcl2, Survivin, and TGF gene expression levels [36]. Thus, the distinct structural and temporal characteristics of LAB-oligosaccharides appear to confer a refined and potentially more targeted anticancer activity compared with the earlier preparation.

After extracting the bioactive oligosaccharides from the fermentation medium, we test the efficacy of the remaining lactic acid bacterial biomass as a nutrient-rich substrate for other microorganisms to produce valuable enzymes. This process reflects a natural ecological phenomenon of resource recycling, often compared to microbial cannibalism, and is increasingly recognized as an important strategy in biotechnology and circular bioeconomy systems [37, 38]. The predatory behaviour is not commonly observed in microorganisms. However, the microbial cannibalistic tendencies have been observed in microorganisms under stress; for example, in mixed cultures and under nutritional limitation, *B. subtilis* produces antimicrobial peptides to kill a fraction of its own population (or closely related microbe). The surviving cells then use the lysed cells as a source of nutrients [39]. In the current study, the isolated and identified strain *Bacillus subtilis* 2F (PX205316) this strain showed a close relatedness to other Bacillus species and strains, including *B. subtilis* and *B. cereus,* and the closest phylogenetic cluster was a phylogenetic group that combines several seratia strains. The isolated strain demonstrated a strong ability to utilize *L. acidophilus* biomass as a nutrient-rich substrate under nutrient-depleted culture conditions [38]. By degrading the biomass of the dead *L. acidophilus* cells, the released nutrients were utilized via *B. subtilis* 2F, which were subsequently assimilated to support its growth and drive the efficient production of commercially valuable enzymes, including lipase and protease. This microbial recycling approach shall provide a sustainable, zero-waste strategy for producing valuable enzymes post LAB-oligosaccharides production in batch fermentation. The ability of both *Bacillus* and *Serratia* genera to produce a plethora of enzymes, such as lipases and proteases, which were produced and used in several industrial applications for decades, was previously confirmed [40, 41]. For example, *S. marcescens*-VT1 was found to produce extracellular protease and cold-active (psychrophilic) and stable lipase [40], which strongly supports our valuable findings in this study. This integrated study bridges critical gaps between bioprocess engineering and oncotherapeutic discovery via establishing a sustainable and economically viable biomanufacturing platform.

In the current study, we demonstrates the effective integration between waste valorization and lactic acid bacteria (LAB) bioprocessing fields. The fermentation residues of *Lactobacillus acidophilus* were successfully used as raw material for the production of lipase and protease enzymes. This approach strongly aligns with the circular bioeconomy principles that promotes the converting of fermentation wastes into value-added products instead of their disposal. This idea is supported by a recent work on food-waste fermentation confirmed that LAB-based processes can be integrated into resource recovery systems, improving substrate utilization and sustainability [42]. Our isolate *Bacillus subtilis* PX205316 showed successful production of lipase enzyme at 1.5 U/mL (without oil) and 1.98 U/mL (with oil) using LAB biomass alone. These values are comparable with non-optimized waste-based systems but considered low when compared with optimized media. For instance, optimized condition resulted in the production of lipase activities up to 19 U/mL using *Lacticaseibacillus paracasei*, confirming that both of supplementation and process optimization can significantly enhance the enzyme yield. However, our study confirmed that LAB waste alone can support the enzyme biosynthesis without any more additives. It is noteworthy that the current study strongly supports the zero-waste principle, where LAB biomass (process byproduct) that considered an environmental burden has been successfully recycled into value-added products (enzymes) as a productive input stream. In a recent study, similar circular approach has been emphasized through the conversion of agriculture and food waste into lactic acid and other products, confirming the vital role of microbes in waste minimization [43]. In addition, enzymatic and microbial recycling of organic waste has been shown to enhance nutrient recovery and overall process efficiency in integrated biorefineries [43]. We are planning to complet an optimization bioprocess for enzymes production using the biomass as a potential carbone and nitrogen substrate to maximize the amount of enzymes produced through out the process. Also, we are planning a head for the quantitative determination of proteases, which is missing in the current study, during the anticipated optimzation process.

Overall, although the enzyme yields reported here are moderate compared with fully optimized systems, the study demonstrates a highly sustainable and economically favorable strategy. The conversion of LAB fermentation waste into lipase and protease not only reduces environmental impact, but also adds a new value to the production chain, representing a practical implementation of zero-waste bioprocessing and circular biotechnology.

## Conclusion

This study demonstrates the successful pilot-scale production of the bioactive tri-oligosaccharide TRI-OLIGO-LA-20079 from Lactobacillus acidophilus DSMZ 20079 in a 10-L bioreactor with high production efficiency and preserved biological activity. Structural analyses confirmed the identity of the recovered oligosaccharide, while in vitro assays verified its potent anticancer activity against CaCo-2 cells. Furthermore, the residual bacterial biomass was successfully reutilized as a substrate for the production of commercially valuable enzymes, supporting a sustainable, zero-waste bioprocess. These findings provide a strong foundation for the industrial production of TRI-OLIGO-LA-20079 as a promising bioactive ingredient for nutraceutical and functional food applications.

## Supplementary Information

Supplementary Material 1.

Supplementary Material 2.

Supplementary Material 3.

Supplementary Material 4.

Supplementary Material 5.

Supplementary Material 6.

Supplementary Material 7.

## Data Availability

No datasets were generated or analysed during the current study.
