# Supplementary material for: Bio-process development for sustainable, large-scale production of LAB-oligosaccharides as a potential anti-colon-cancer metabolites produced by Lactobacillus acidophilus 20079
Source: Microb Cell Fact. 2026 Aug 12;25:168. doi: 10.1186/s12934-026-03067-x (PMC13474794; doi:10.1186/s12934-026-03067-x)

Lamiaa Youssef PR 4 -M deptq

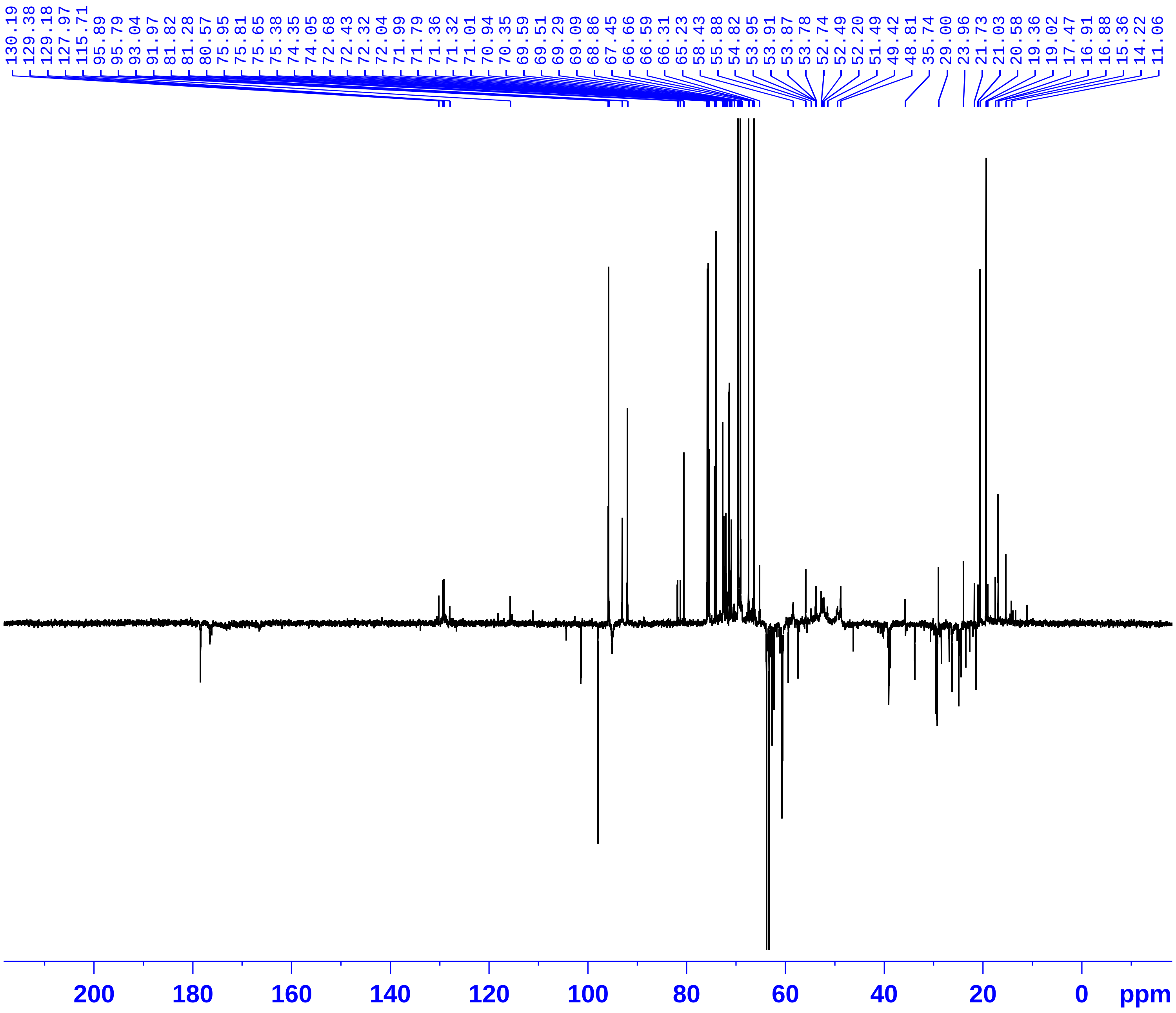

Faculty of Pharmacy

```
Current Data Parameters
NAME       Lamiaa Youssef PR 4 -M deptq
EXPNO                  10
PROCNO                  1

F2 - Acquisition Parameters
Date_             20240606
Time                  9.50 h
INSTRUM              spect
PROBHD   Z108618_0945 (
PULPROG          deptqgpsp
TD                   65536
SOLVENT                D2O
NS                    2000
DS                       8
SWH              23809.523 Hz
FIDRES            0.726609 Hz
AQ               1.3762560 sec
RG                  197.77
DW                  21.000 usec
DE                    6.50 usec
TE                   296.0 K
CNST2          145.0000000
D1              2.00000000 sec
D2              0.00344828 sec
D12             0.00002000 sec
D16             0.00020000 sec
TD0                      1
SFO1           100.6404331 MHz
NUC1                   13C
P1                   10.00 usec
P13                2000.00 usec
PLW0     0 W
PLW1           47.00000000 W
SPNAM[5]    Crp60comp.4
SPOAL5               0.500
SPOFFS5  0 Hz
SPW5            7.18109989 W
SFO2           400.2016008 MHz
NUC2                    1H
CNST12           1.5000000
CPDPRG[2           waltz65
P0                   20.25 usec
P3                   13.50 usec
P4                   27.00 usec
PCPD2                90.00 usec
PLW2           13.00000000 W
PLW12           0.29249999 W
GPNAM[1]       SMSQ10.100
GPZ1                 31.00 %
GPNAM[2]       SMSQ10.100
GPZ2                 31.00 %
GPNAM[3]       SMSQ10.100
GPZ3                 31.00 %
P16                1000.00 usec

F2 - Processing parameters
SI                   32768
SF             100.6303700 MHz
WDW                     EM
SSB      0
LB                    1.00 Hz
GB       0
PC                    1.40
```

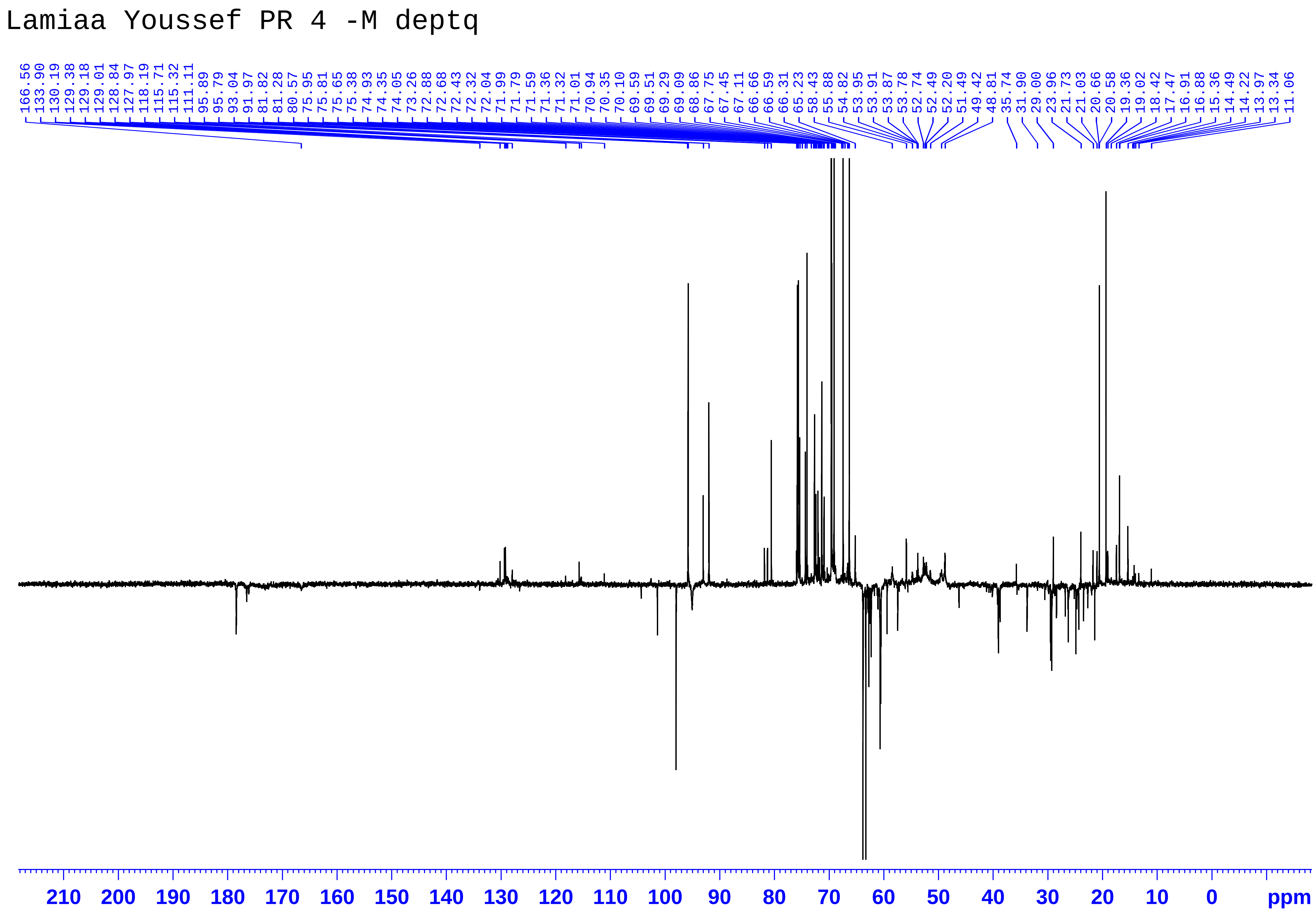

Lamiaa Youssef PR 4 -M deptq
166.56
133.90
130.19
129.38
129.18
129.01
128.84
127.97
118.19
115.71
115.32
111.11
95.89
95.79
93.04
91.97
81.82
81.28
80.57
75.95
75.81
75.65
75.38
74.93
74.35
74.05
73.26
72.88
72.68
72.43
72.32
72.04
71.99
71.79
71.59
71.36
71.32
71.01
70.94
70.35
70.10
69.59
69.51
69.29
69.09
68.86
67.75
67.45
67.11
66.66
66.59
66.31
65.23
58.43
55.88
54.82
53.95
53.91
53.87
53.78
52.74
52.49
52.20
51.49
49.42
48.81
35.74
31.90
29.00
23.96
21.73
21.03
20.66
20.58
19.36
19.02
18.42
17.47
16.91
16.88
15.36
14.49
14.22
13.97
13.34
11.06
210
200
190
180
170
160
150
140
130
120
110
100
90
80
70
60
50
40
30
20
10
0
ppm

Lamiaa Youssef PR 4 -M deptq

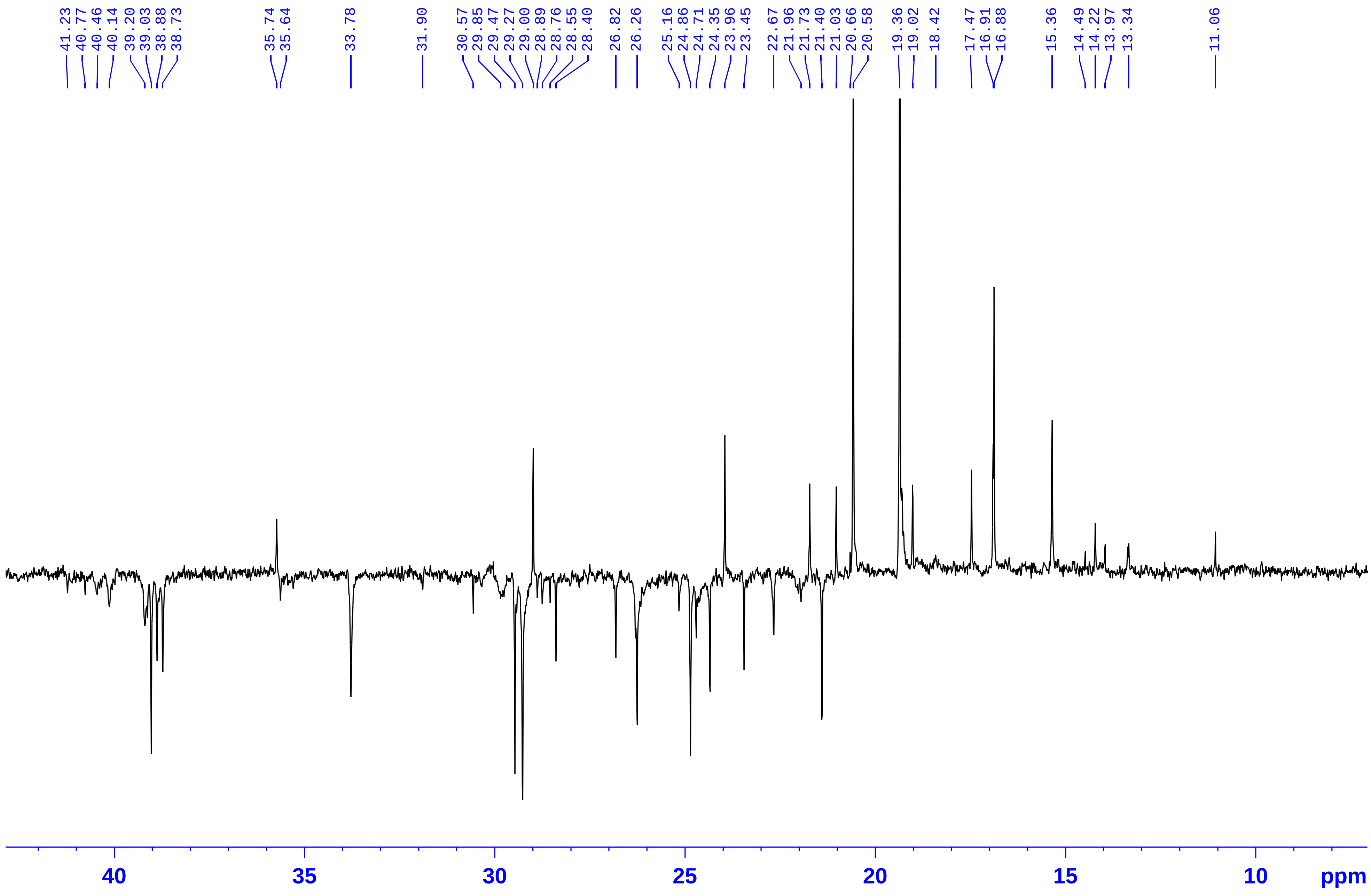

Lamiaa Youssef PR 4 -M deptq

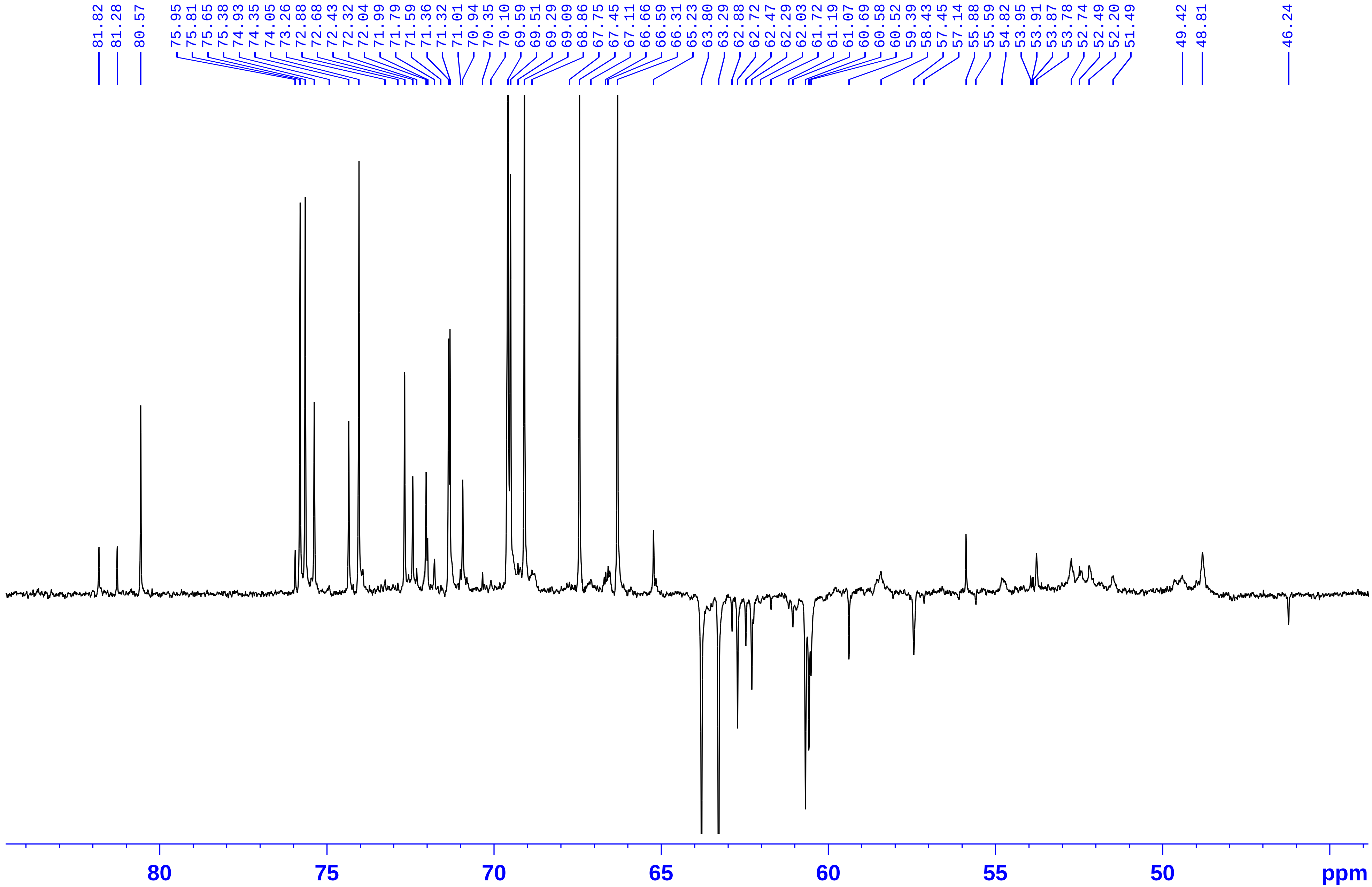

Lamiaa Youssef PR 4 -M deptq

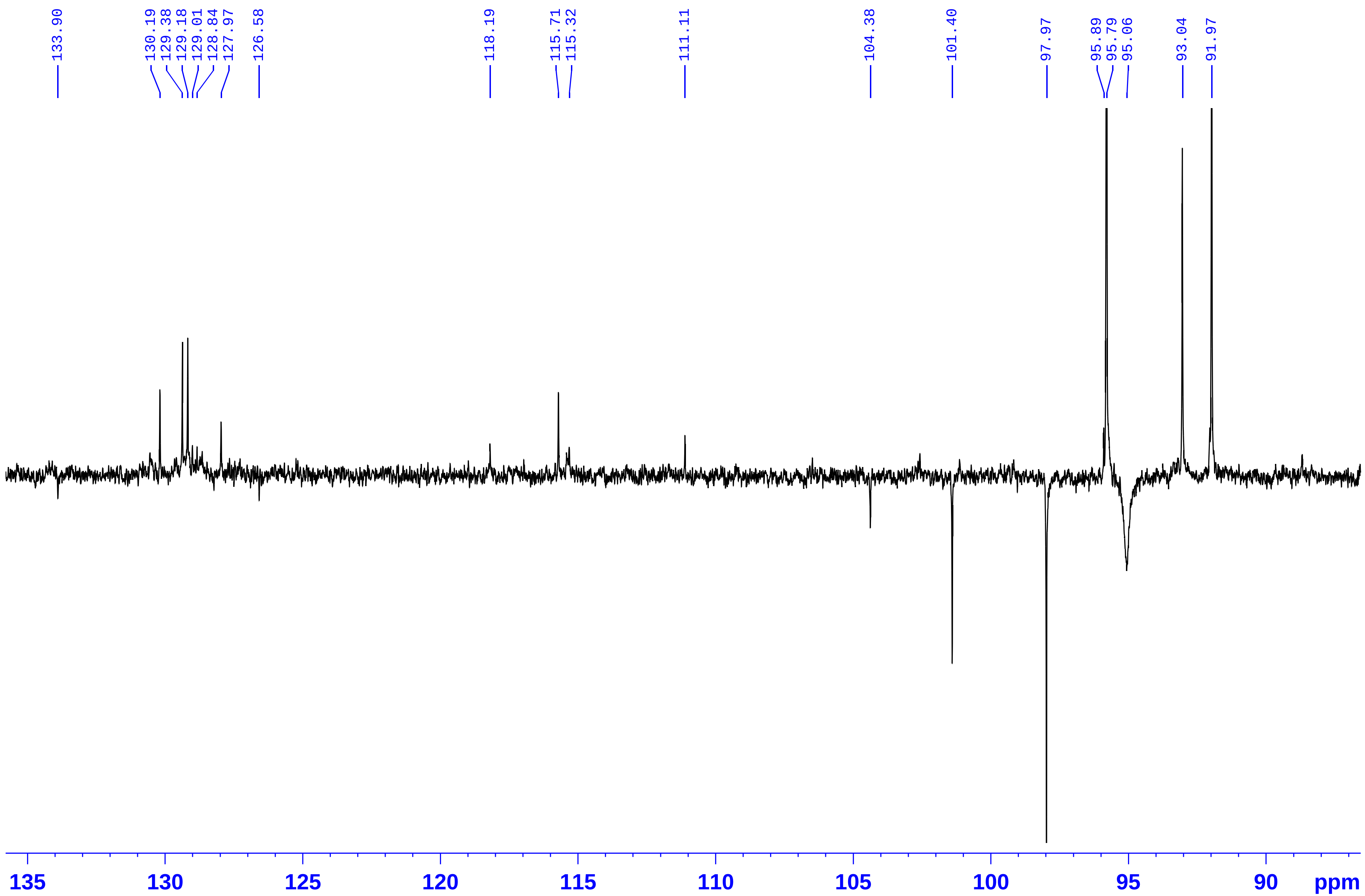

Lamiaa Youssef PR 4 -M deptq

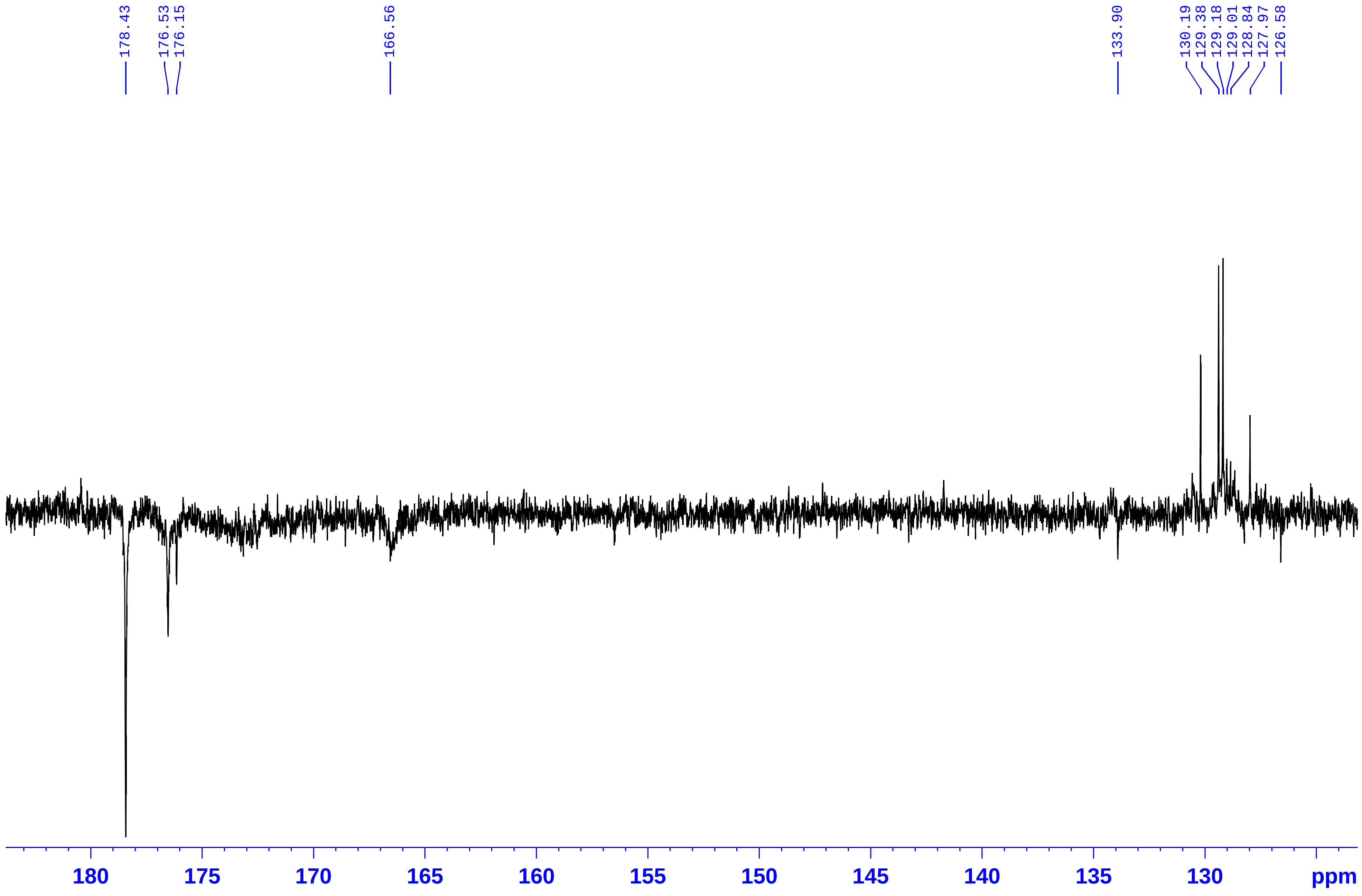

Supplement: Supplementary file 1 [file 12934_2026_3067_MOESM1_ESM.pdf]
