# Supplementary material for: Bio-process development for sustainable, large-scale production of LAB-oligosaccharides as a potential anti-colon-cancer metabolites produced by Lactobacillus acidophilus 20079
Source: Microb Cell Fact. 2026 Aug 12;25:168. doi: 10.1186/s12934-026-03067-x (PMC13474794; doi:10.1186/s12934-026-03067-x)

Lamiaa Abd Allah-PR4-proton-D2O-AS

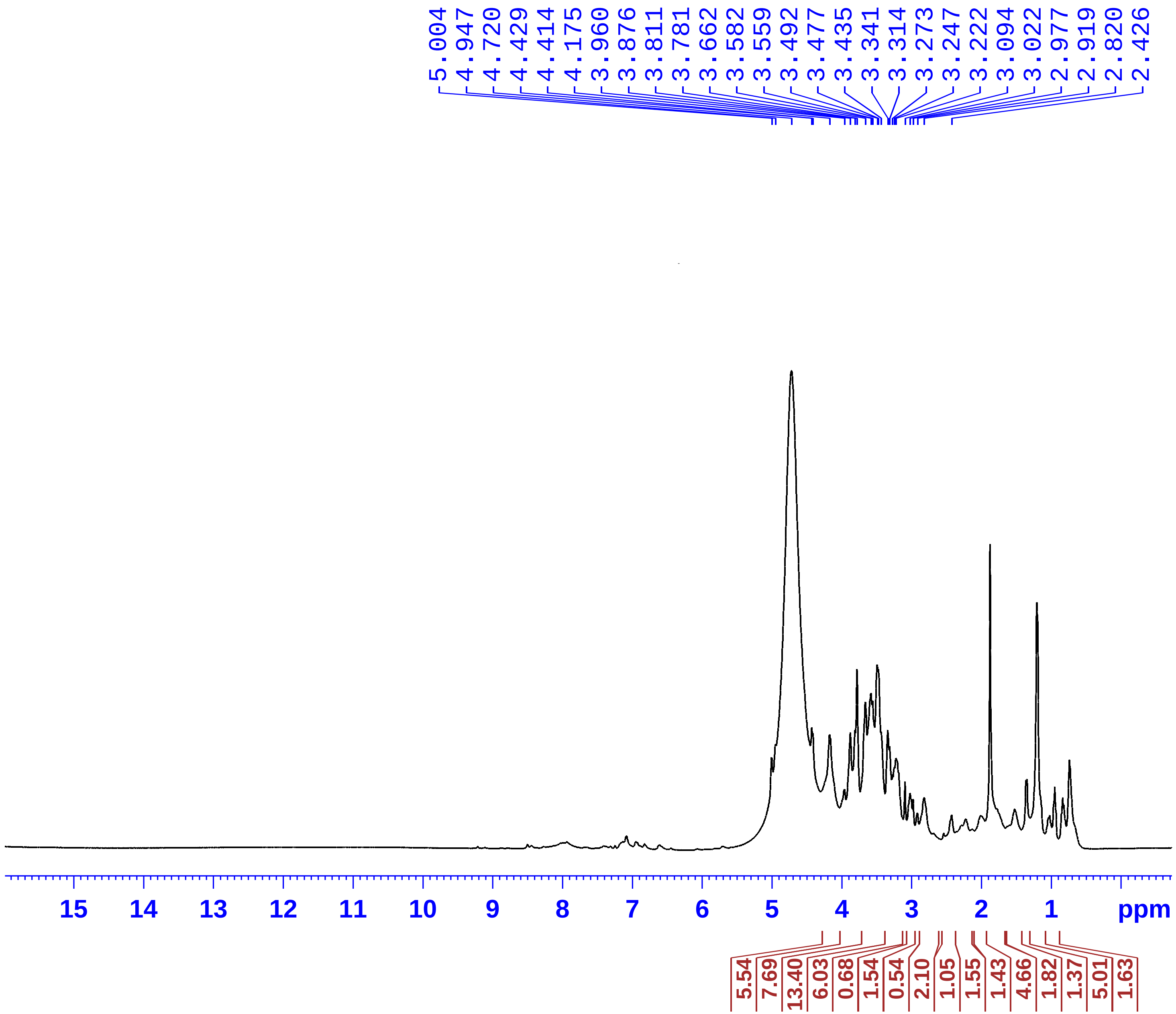

BRUKER

Faculty of Pharmacy

```
Current Data Parameters
NAME      Lamiaa Abd Allah-PR4-proton-D2O-AS
EXPNO                     10
PROCNO                     1

F2 - Acquisition Parameters
Date_               20240602
Time                   12.12 h
INSTRUM                spect
PROBHD    Z108618_0945 (
PULPROG                 zg30
TD                     65536
SOLVENT                  D2O
NS                        16
DS                         2
SWH                 8012.820 Hz
FIDRES              0.244532 Hz
AQ                 4.0894465 sec
RG                     17.41
DW                    62.400 usec
DE                      6.50 usec
TE                     295.5 K
D1                1.00000000 sec
TD0                        1
SFO1             400.2024712 MHz
NUC1                      1H
P1                     13.50 usec
PLW1             13.00000000 W

F2 - Processing parameters
SI                     65536
SF               400.2000000 MHz
WDW                       EM
SSB       0
LB                      0.30 Hz
GB        0
PC                      1.00
```

Lamiaa Abd Allah-PR4-proton-D2O-AS

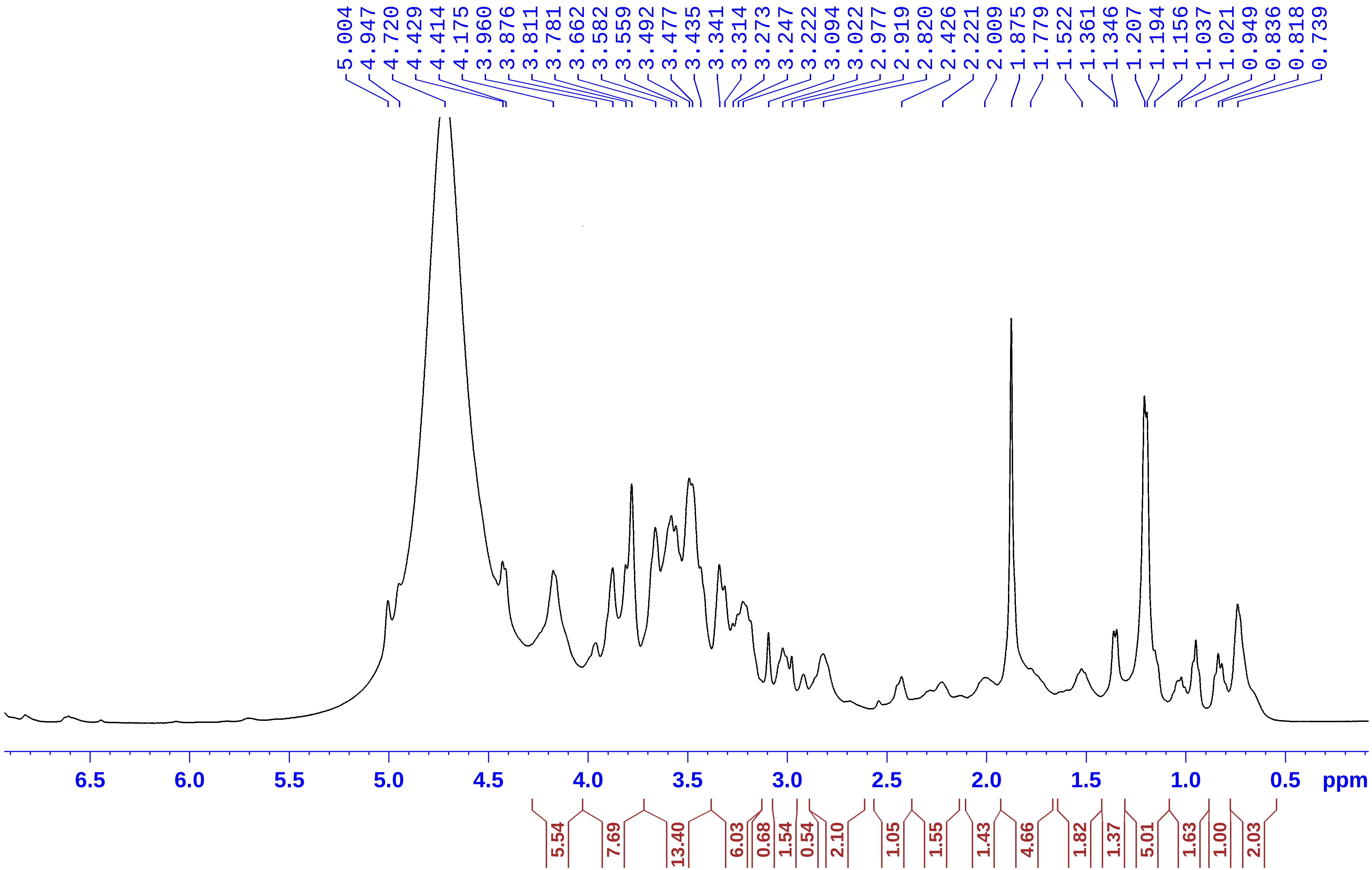

Supplement: Supplementary file 2 [file 12934_2026_3067_MOESM2_ESM.pdf]
