# Supplementary material for: Bio-process development for sustainable, large-scale production of LAB-oligosaccharides as a potential anti-colon-cancer metabolites produced by Lactobacillus acidophilus 20079
Source: Microb Cell Fact. 2026 Aug 12;25:168. doi: 10.1186/s12934-026-03067-x (PMC13474794; doi:10.1186/s12934-026-03067-x)

Lamiaa Abdullah-PR4-NOESY-RR

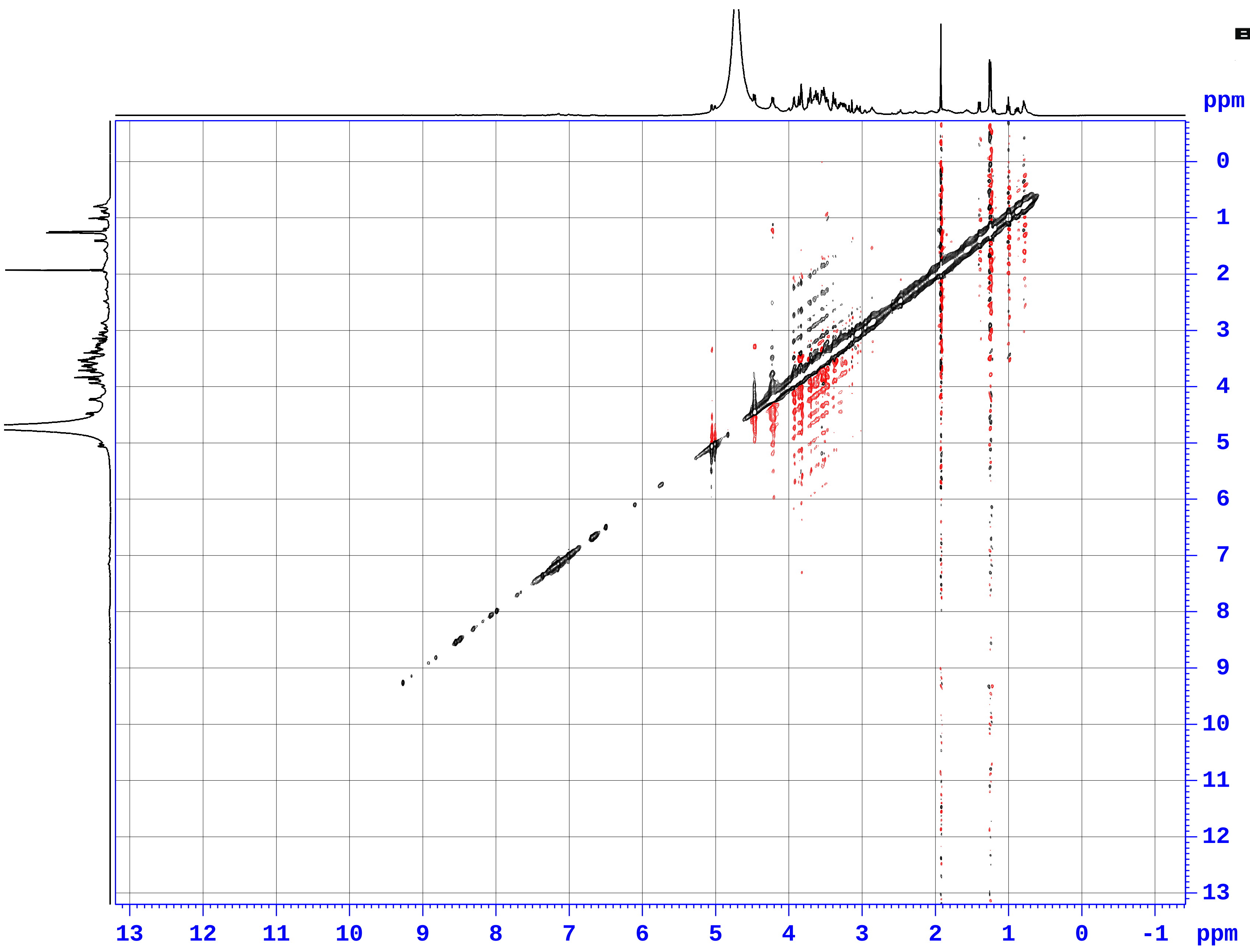

Faculty of Pharmacy

```
Current Data Parameters
NAME       Lamiaa Abdullah-PR4-2D-RR
EXPNO                    14
PROCNO                    1

F2 - Acquisition Parameters
Date_              20240626
Time                  22.38 h
INSTRUM               spect
PROBHD     Z108618_0945 (
PULPROG           noesyphpr
TD                     2048
SOLVENT                 D2O
NS                       12
DS                       16
SWH                6802.620 Hz
FIDRES             6.643183 Hz
AQ                0.1505302 sec
RG                    18.84
DW                   73.501 usec
DE                     6.50 usec
TE                    295.6 K
D0               0.00005631 sec
D1               2.00000000 sec
D8               0.30000001 sec
D11              0.03000000 sec
D12              0.00002000 sec
D13              0.00000400 sec
IN0              0.00014700 sec
TDav                      1
SFO1            400.2018809 MHz
NUC1                     1H
P1                    13.50 usec
PLW1            13.00000000 W
PLW9             0.00009477 W

F1 - Acquisition parameters
TD                      256
SFO1               400.2019 MHz
FIDRES            53.146259 Hz
SW                   16.998 ppm
FnMODE          States-TPPI

F2 - Processing parameters
SI                     2048
SF              400.2000000 MHz
WDW                   QSINE
SSB                       2
LB                        0 Hz
GB                        0
PC                     1.00

F1 - Processing parameters
SI                     1024
MC2             States-TPPI
SF              400.2000000 MHz
WDW                   QSINE
SSB                       2
LB                        0 Hz
GB                        0
```

Lamiaa Abdullah-PR4-NOESY-RR

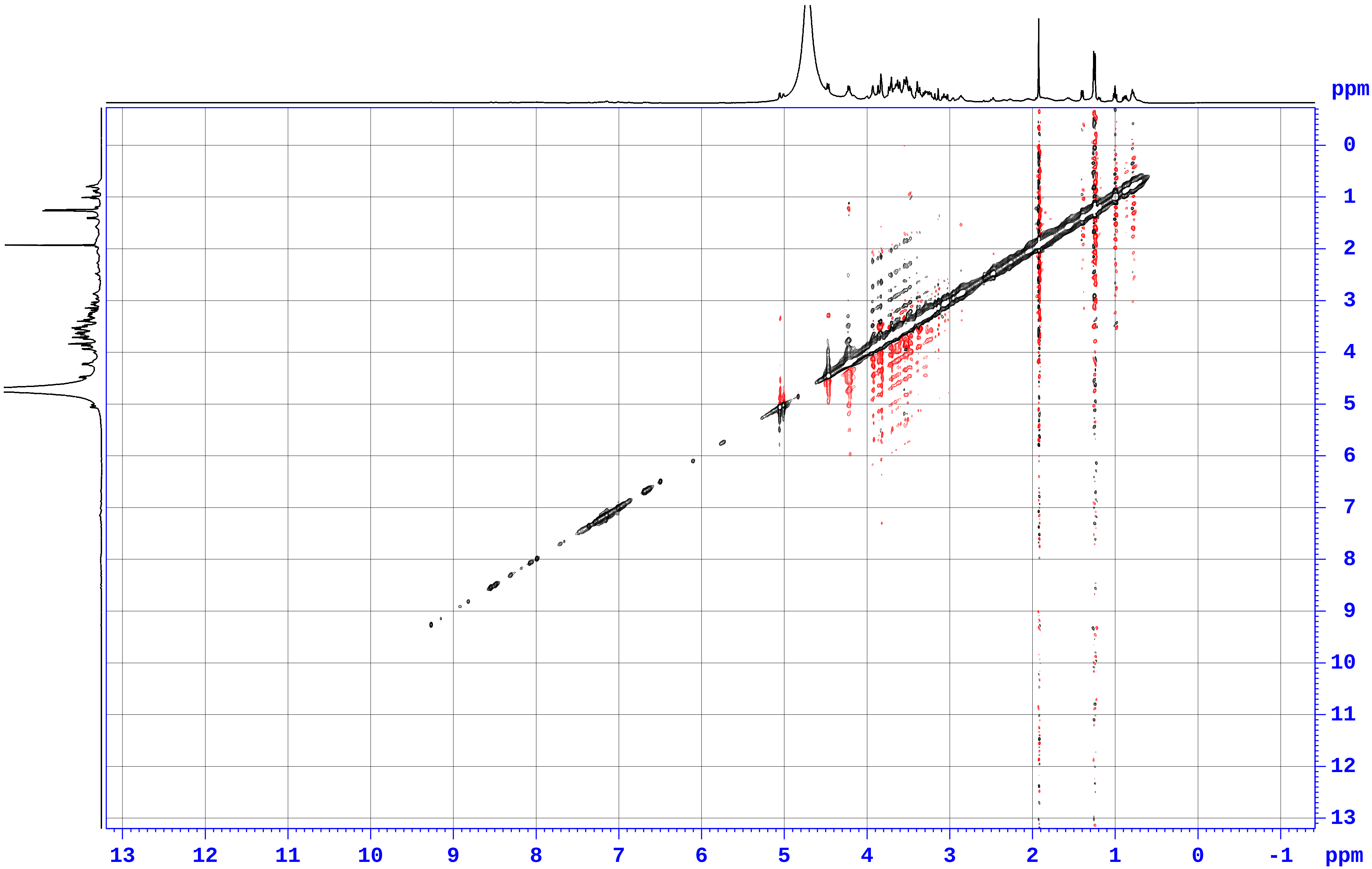

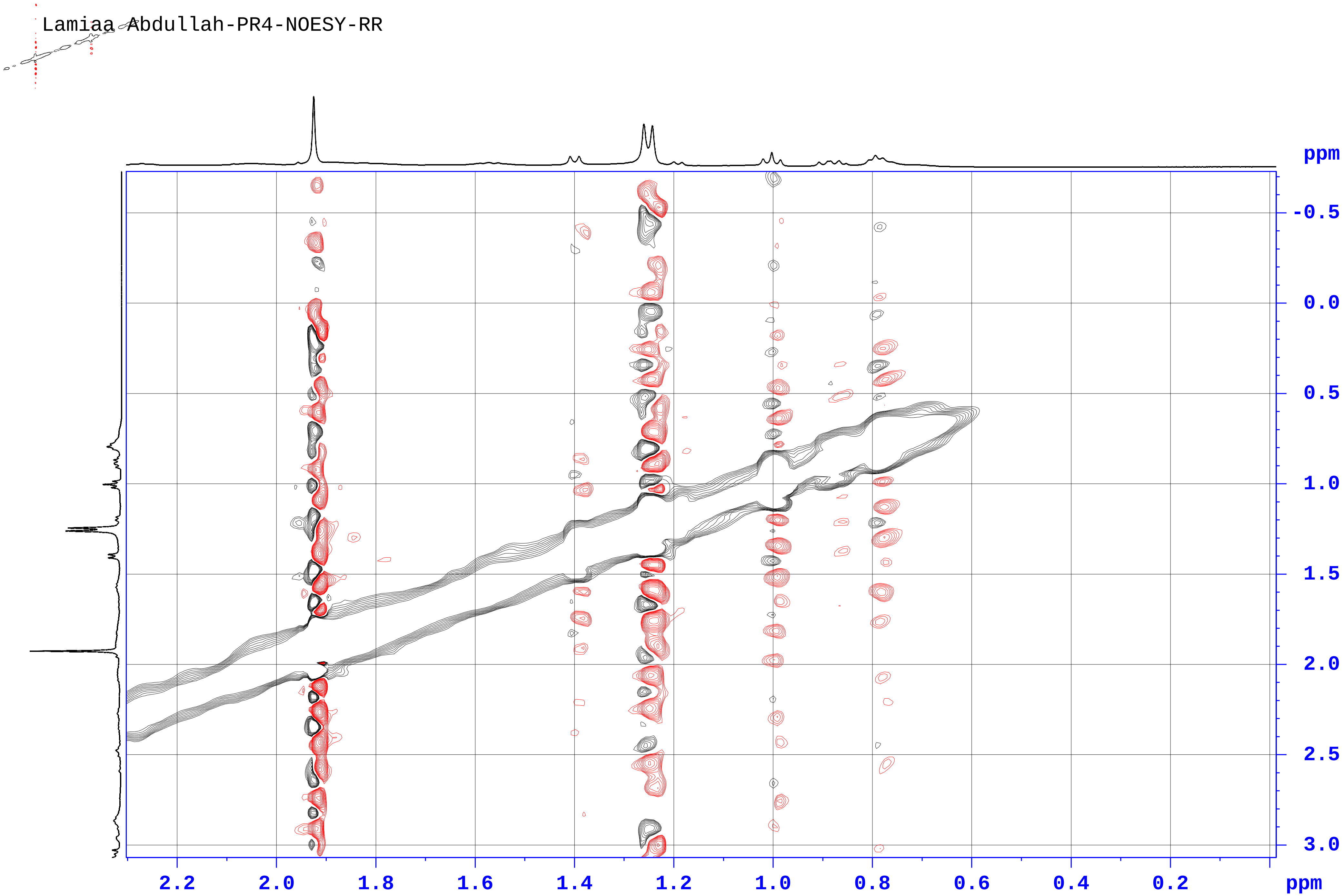
Lamiaa Abdullah-PR4-NOESY-RR
ppm
-0.5
0.0
0.5
1.0
1.5
2.0
2.5
3.0
2.2
2.0
1.8
1.6
1.4
1.2
1.0
0.8
0.6
0.4
0.2
ppm

Lamiaa Abdullah-PR4-NOESY-RR

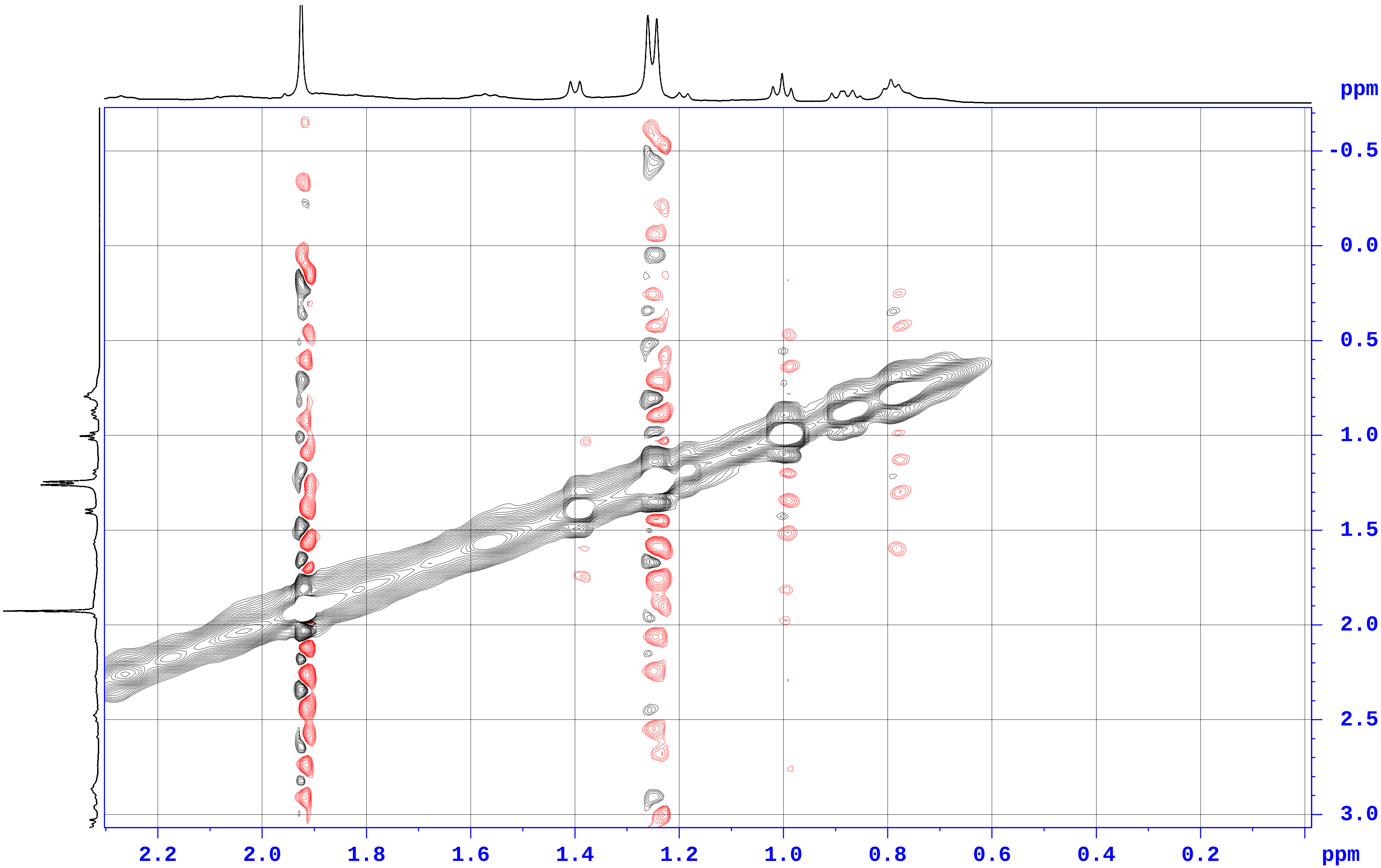

Lamiaa Abdullah-PR4-NOESY-RR

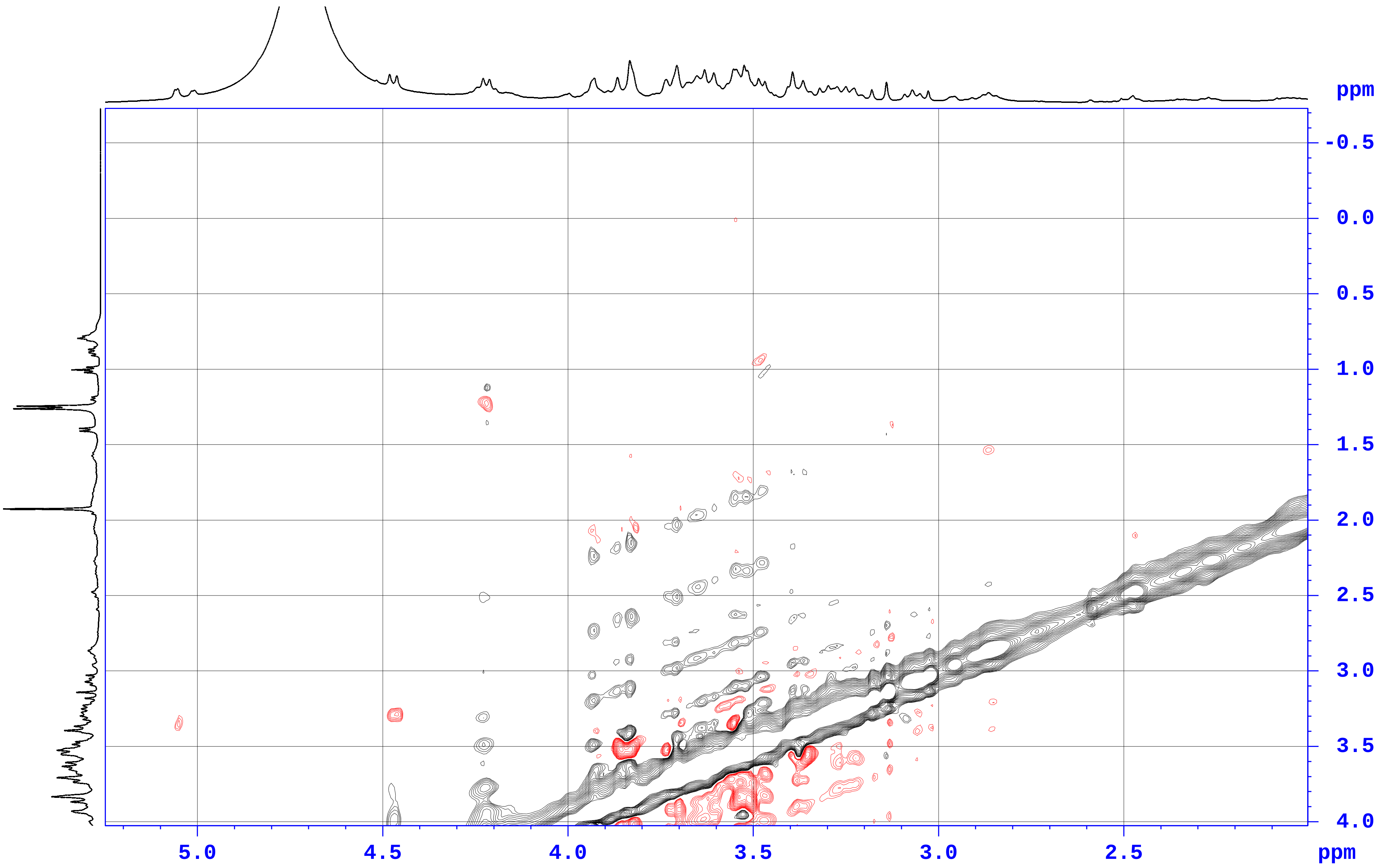

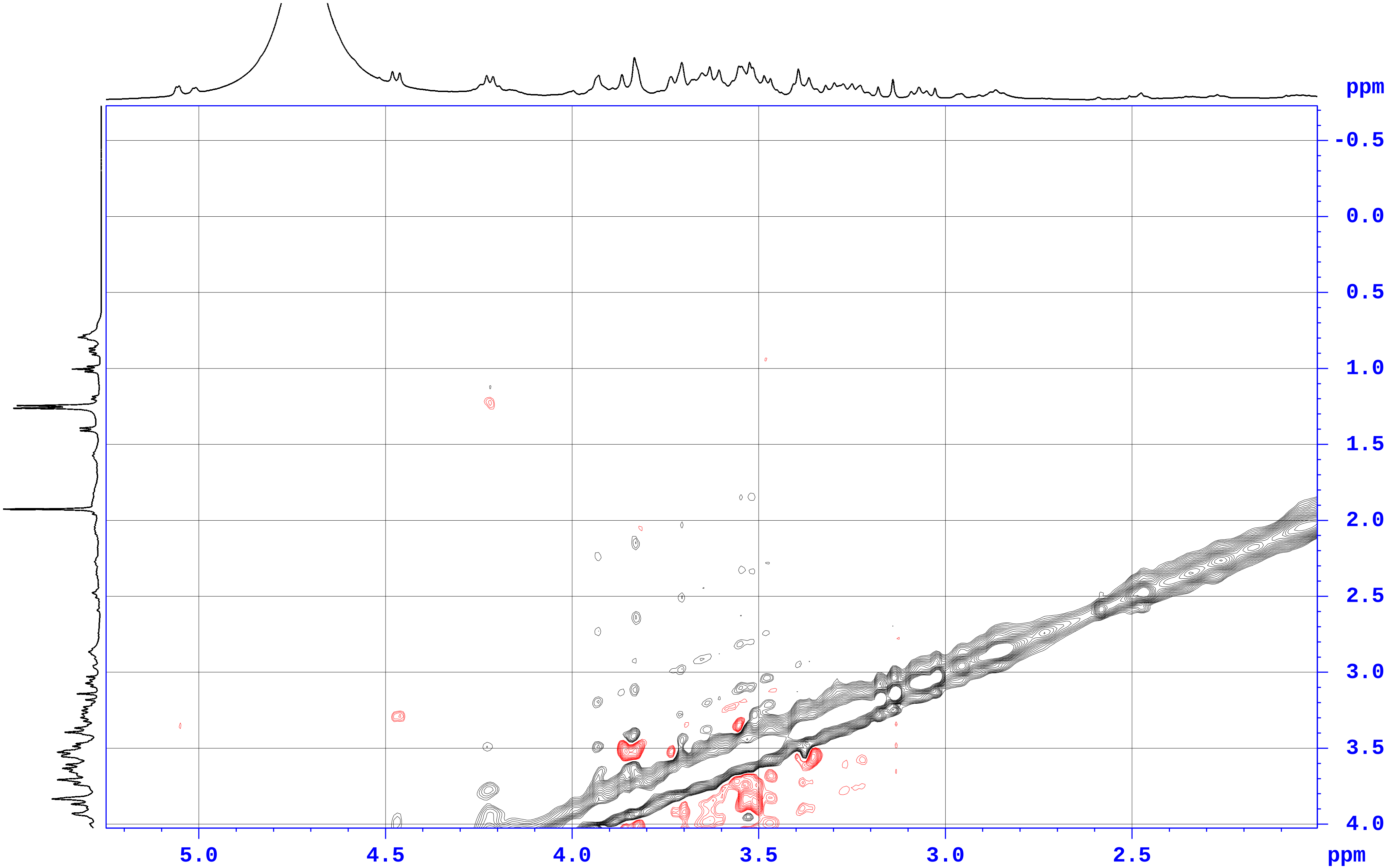
ppm
-0.5
0.0
0.5
1.0
1.5
2.0
2.5
3.0
3.5
4.0
5.0
4.5
4.0
3.5
3.0
2.5
ppm

Lamiaa Abdullah-PR4-NOESY-RR

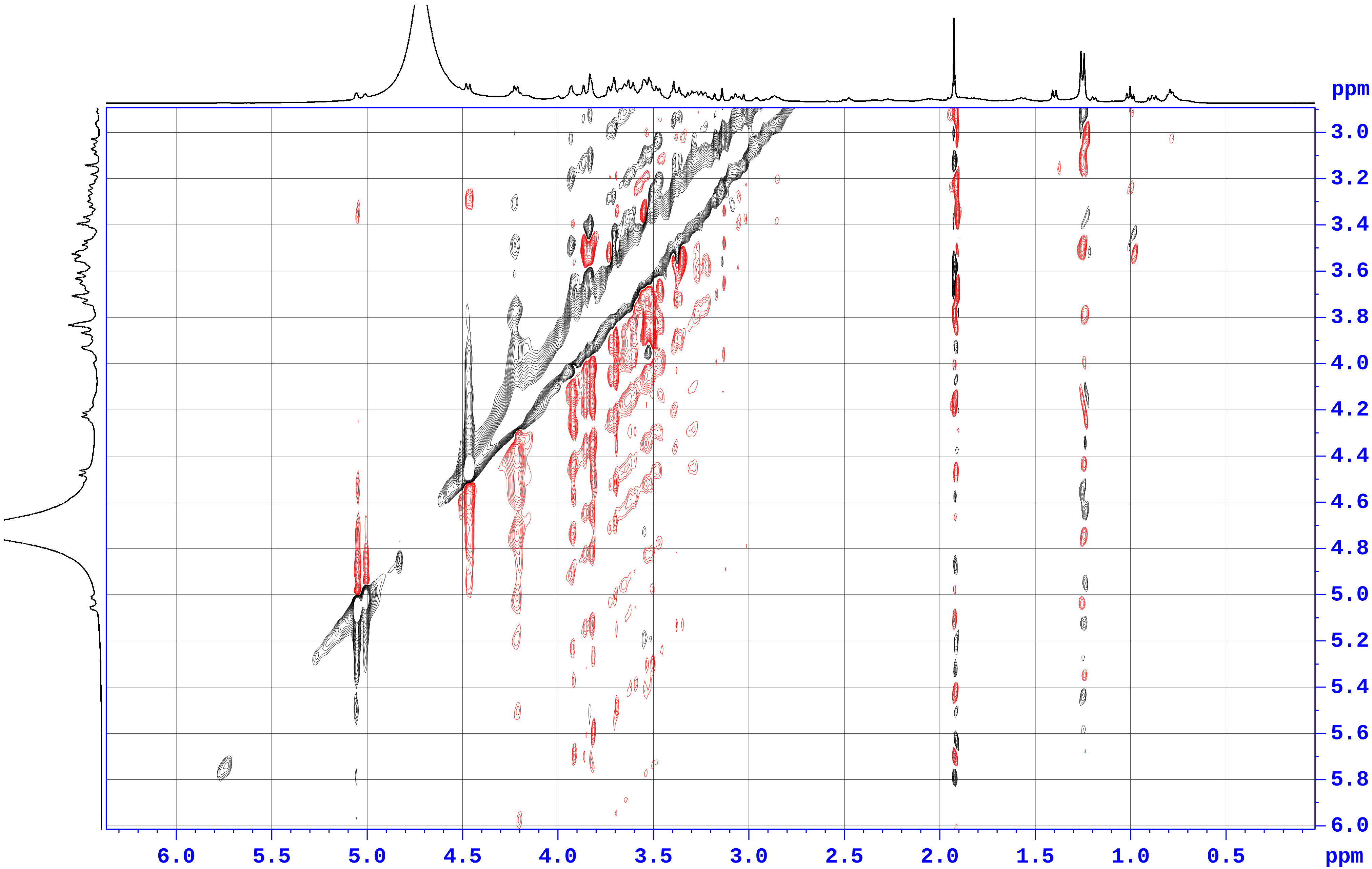

Lamiaa Abdullah-PR4-NOESY-RR

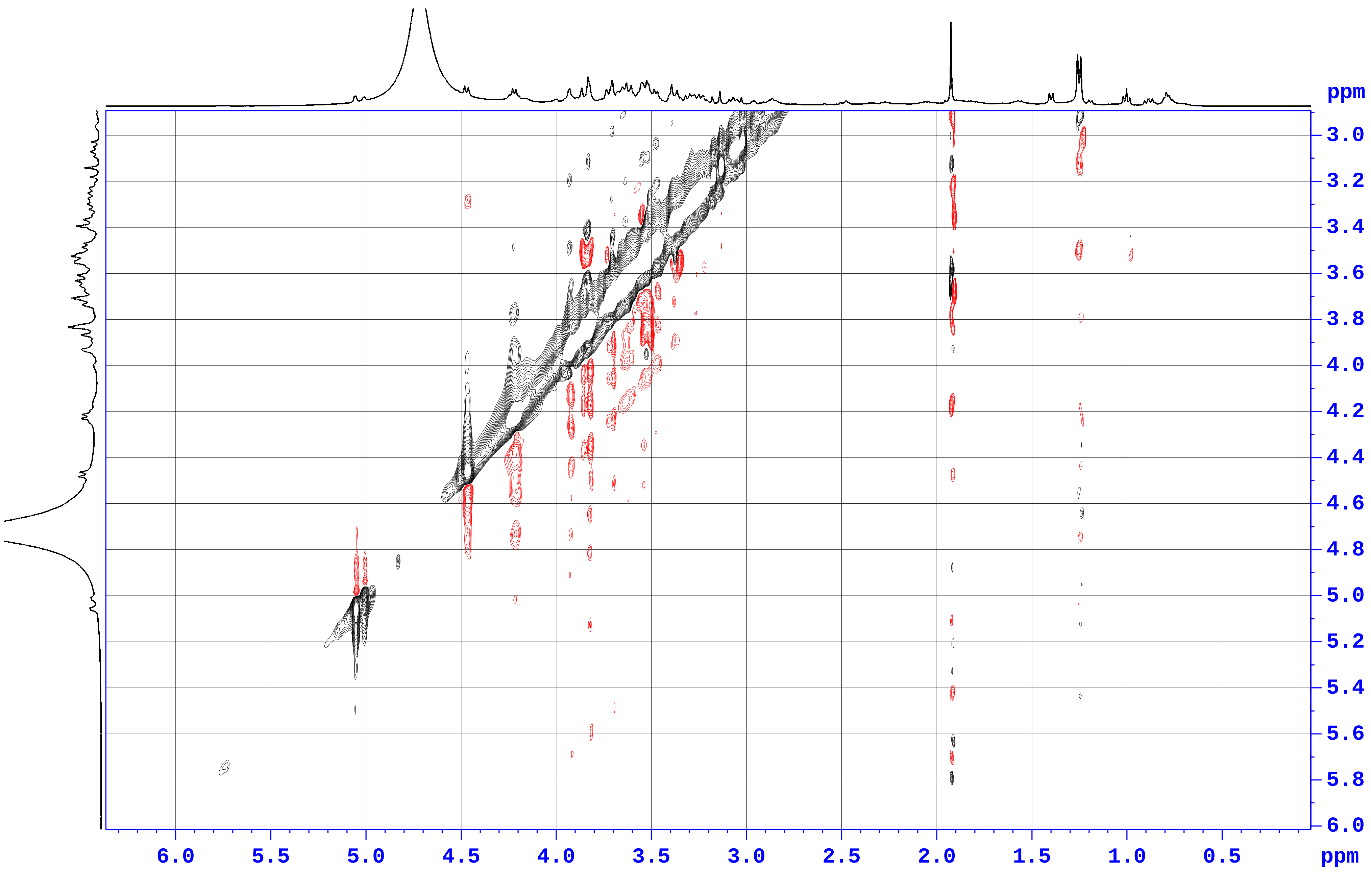

Lamiaa Abdullah-PR4-NOESY-RR

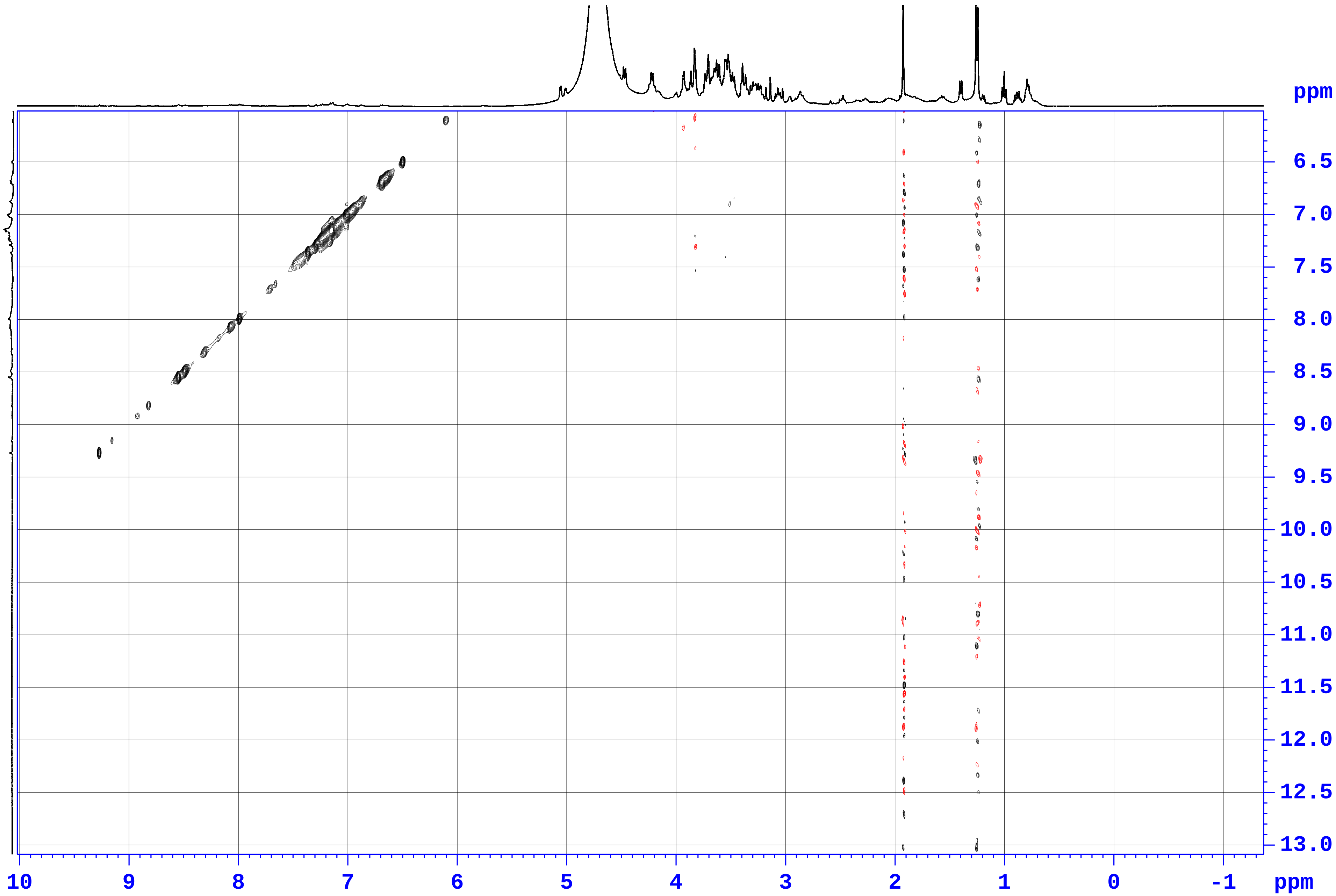

Lamiaa Abdullah-PR4-NOESY-RR

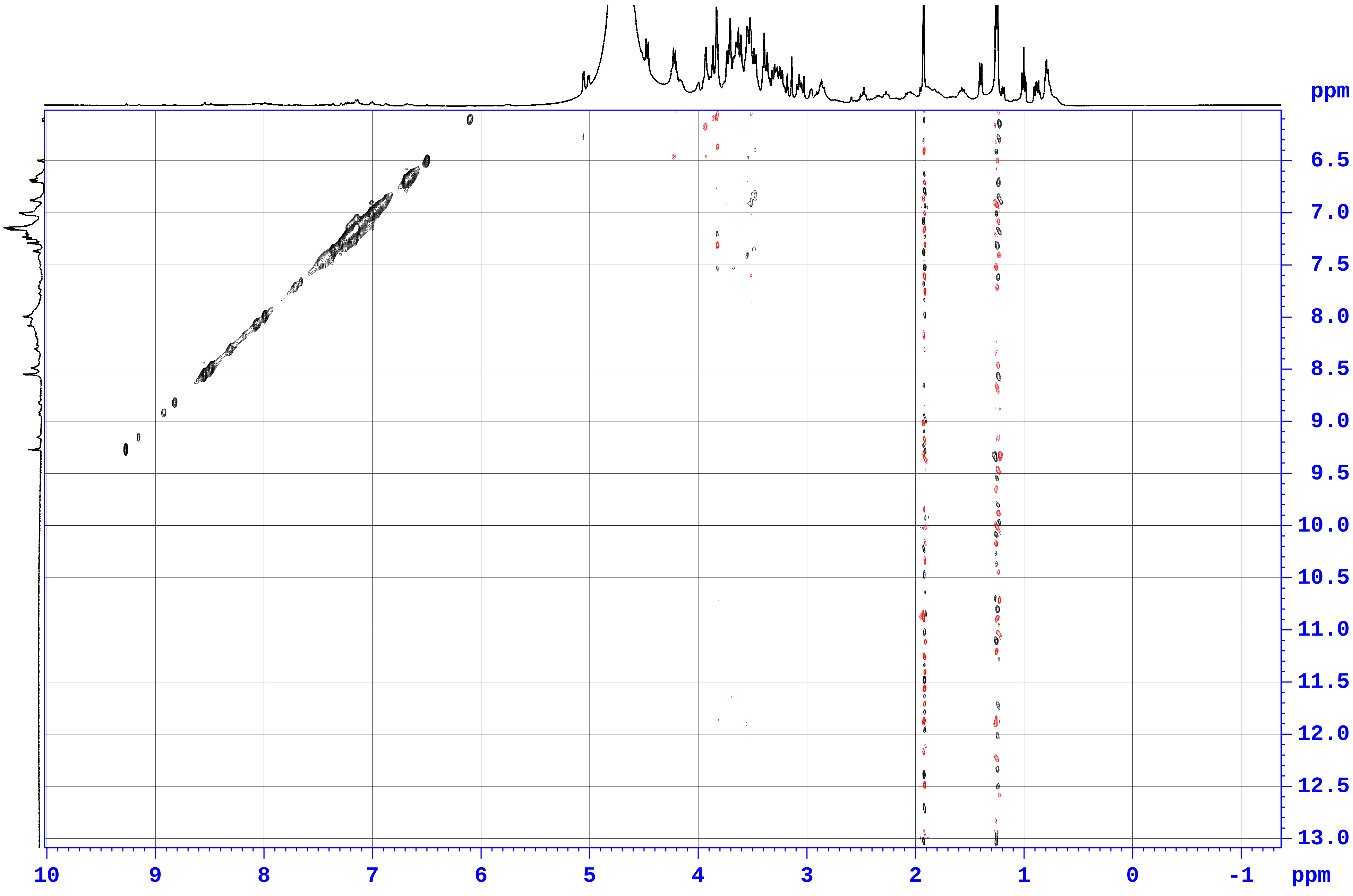

Supplement: Supplementary file 3 [file 12934_2026_3067_MOESM3_ESM.pdf]
