# Supplementary material for: Bio-process development for sustainable, large-scale production of LAB-oligosaccharides as a potential anti-colon-cancer metabolites produced by Lactobacillus acidophilus 20079
Source: Microb Cell Fact. 2026 Aug 12;25:168. doi: 10.1186/s12934-026-03067-x (PMC13474794; doi:10.1186/s12934-026-03067-x)

Lamiaa Abdullah-PR4-HSQC-RR

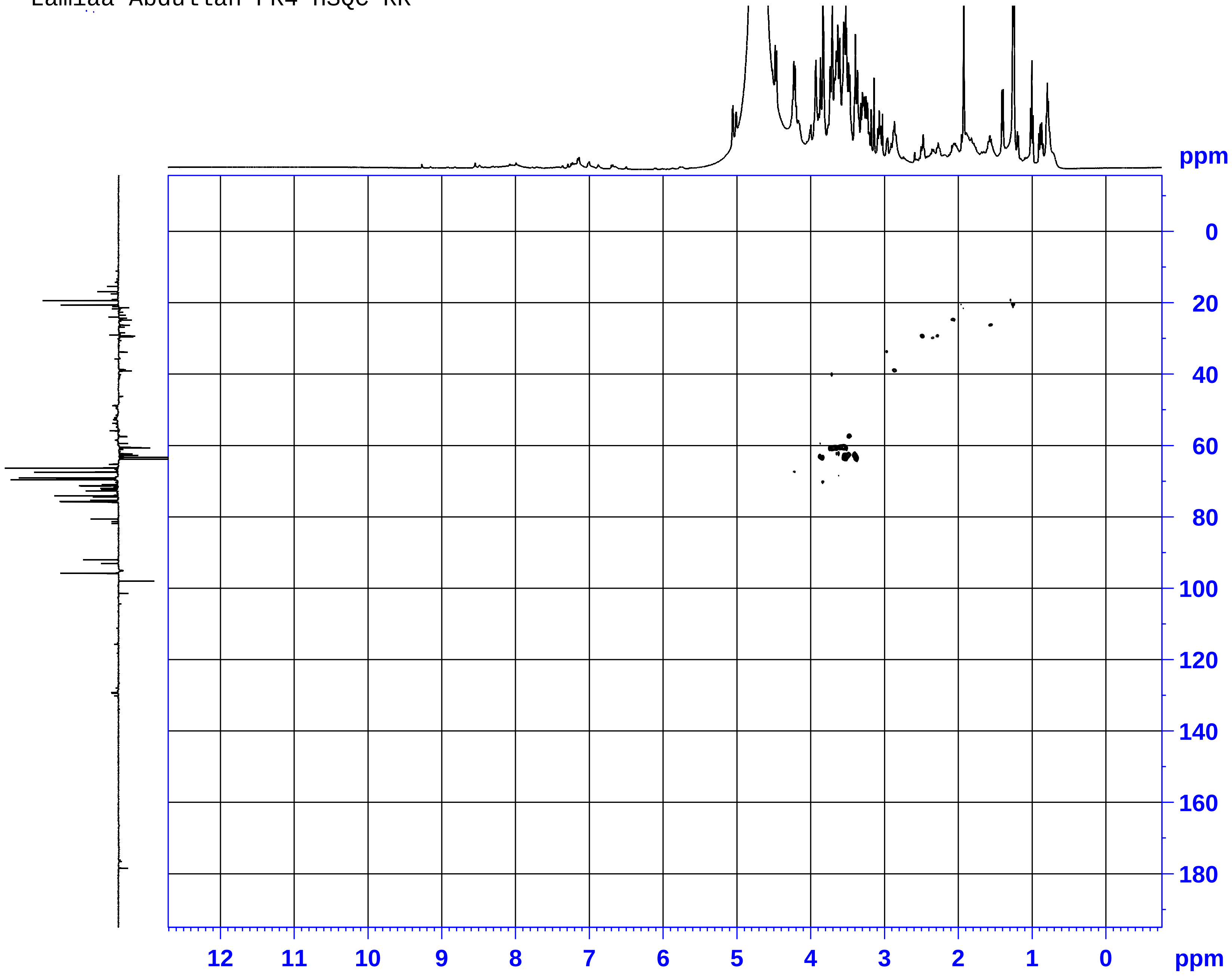

BRUKER

Faculty of Pharmacy

```
Current Data Parameters
NAME      Lamiaa Abdullah-PR4-2D-RR
EXPNO                    13
PROCNO                    1

F2 - Acquisition Parameters
Date_              20240626
Time                  20.26 h
INSTRUM               spect
PROBHD    Z108618_0945 (
PULPROG   hsqcedetgpsisp2.3
TD                     2048
SOLVENT                 D2O
NS                       14
DS                       32
SWH                6410.256 Hz
FIDRES             6.260016 Hz
AQ                0.1597440 sec
RG                   197.77
DW                   78.000 usec
DE                     6.50 usec
TE                    295.7 K
CNST2           145.0000000
CNST17           -0.5000000
D0               0.00000300 sec
D1               2.00000000 sec
D4               0.00172414 sec
D11              0.03000000 sec
D16              0.00020000 sec
D21              0.00360000 sec
D24              0.00089000 sec
IN0              0.00002070 sec
TDav                      1
SFO1            400.2018809 MHz
NUC1                     1H
P1                    13.50 usec
P2                    27.00 usec
P28       0 usec
PLW1            13.00000000 W
SFO2            100.6379173 MHz
NUC2                    13C
CPDPRG[2               garp
P3                    10.00 usec
P14                  500.00 usec
P24                 2000.00 usec
P31                 2119.00 usec
PCPD2                 80.00 usec
PLW0      0 W
PLW2            47.00000000 W
PLW12            0.73438001 W
SPNAM[3] Crp60,0.5,20.1
SPOAL3                0.500
SPOFFS3   0 Hz
SPW3             7.18109989 W
SPNAM[7]     Crp60comp.4
SPOAL7                0.500
SPOFFS7   0 Hz
SPW7             7.18109989 W
SPNAM[18   Crp60_xfilt.2
SPOAL18               0.500
SPOFFS18 0 Hz
SPW18            1.38339996 W
GPNAM[1]      SMSQ10.100
GPZ1                  80.00 %
GPNAM[2]      SMSQ10.100
GPZ2                  20.10 %
GPNAM[3]      SMSQ10.100
GPZ3                  11.00 %
GPNAM[4]      SMSQ10.100
GPZ4                  -5.00 %
P16                 1000.00 usec
P19                  600.00 usec

F1 - Acquisition parameters
TD                      256
SFO1               100.6379 MHz
FIDRES           188.707733 Hz
SW                  240.015 ppm
FnMODE       Echo-Antiecho

F2 - Processing parameters
SI                     1024
SF              400.2000000 MHz
WDW                   QSINE
SSB                       2
LB        0 Hz
GB        0
PC                     1.40

F1 - Processing parameters
SI                     1024
MC2          echo-antiecho
SF              100.6303700 MHz
WDW                   QSINE
SSB                       2
LB        0 Hz
GB        0
```

Lamiaa Abdullah-PR4-HSQC-RR

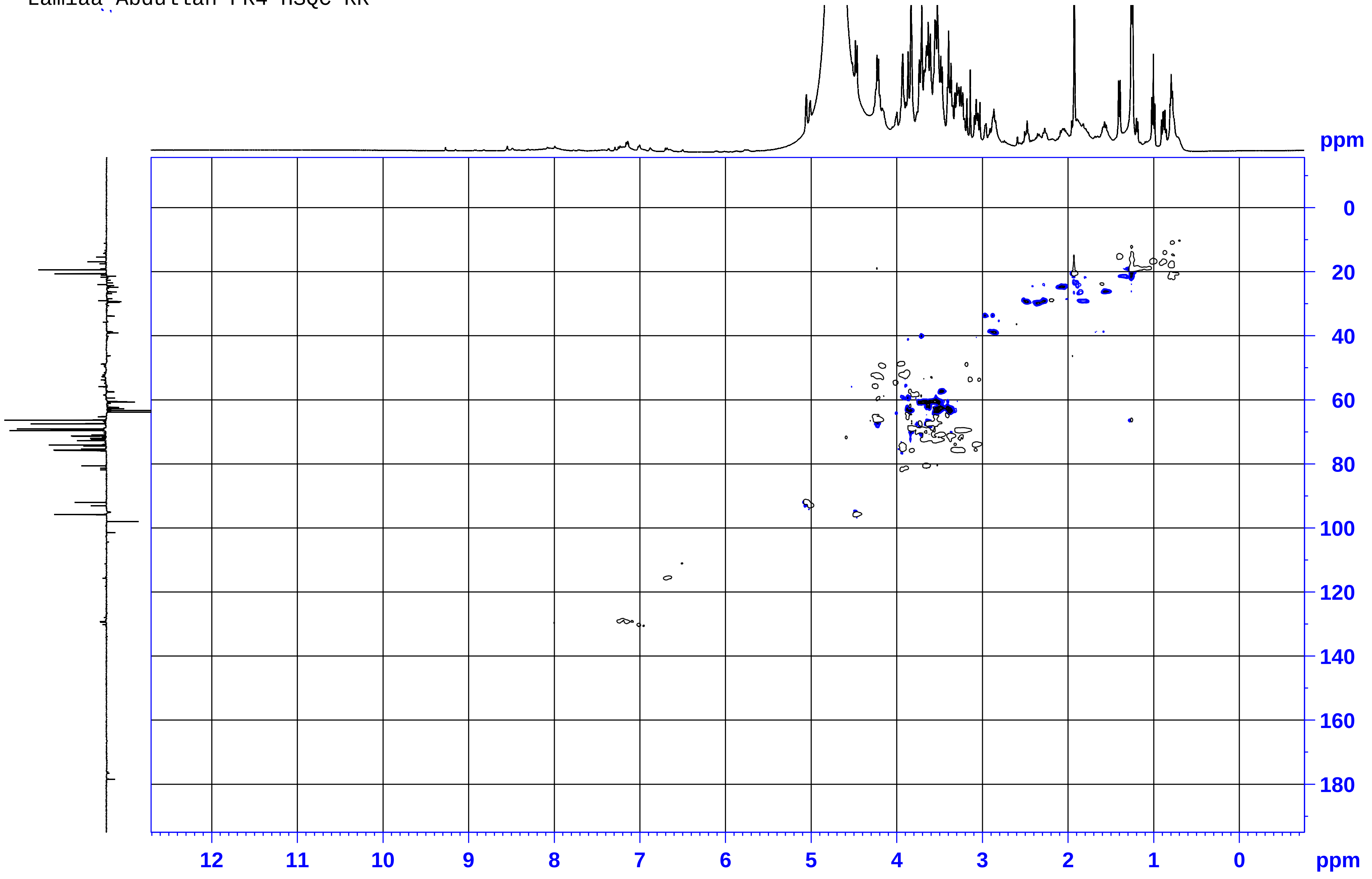

Lamiaa Abdullah-PR4-HSQC-RR

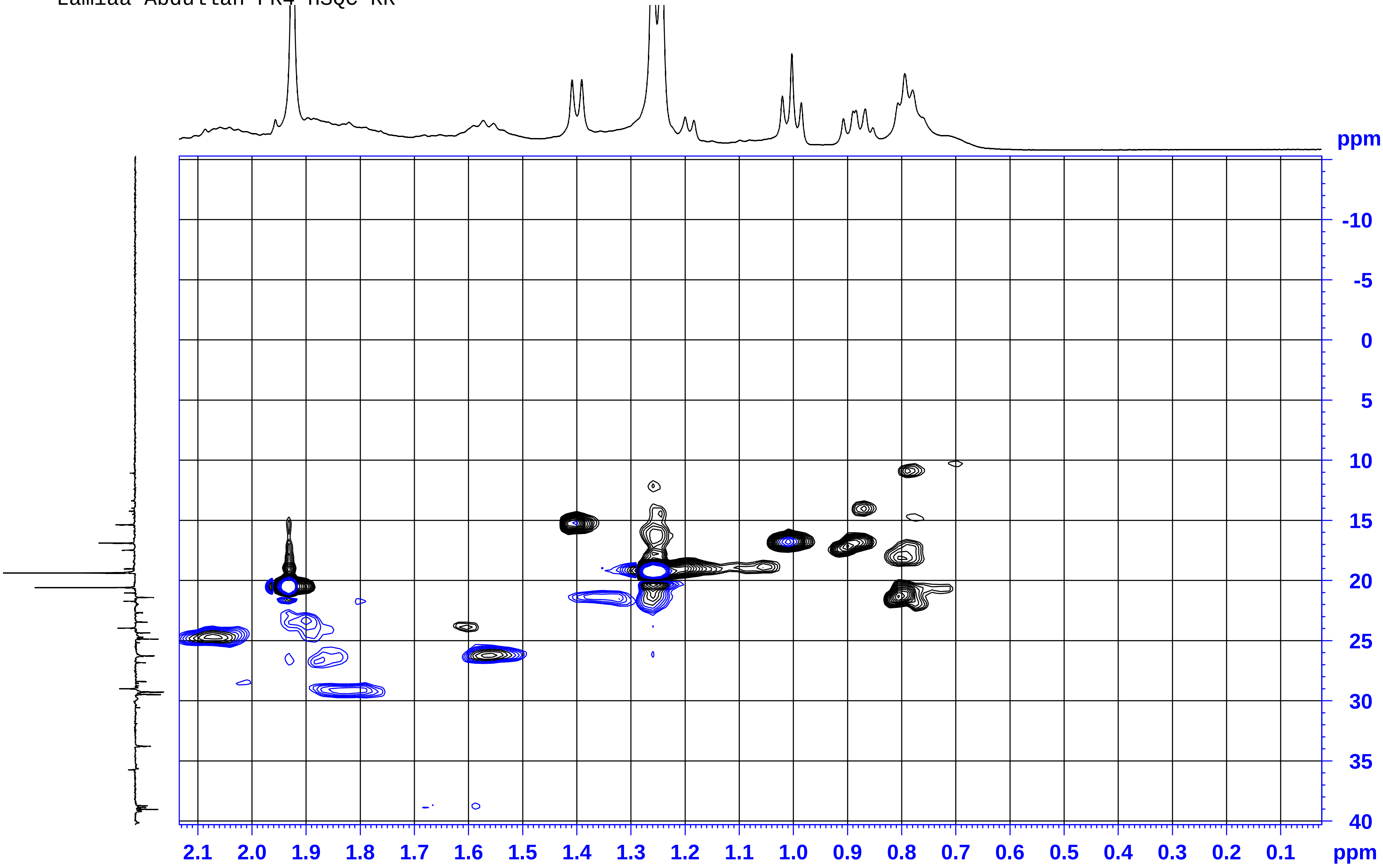

Lamiaa Abdullah-PR4-HSQC-RR

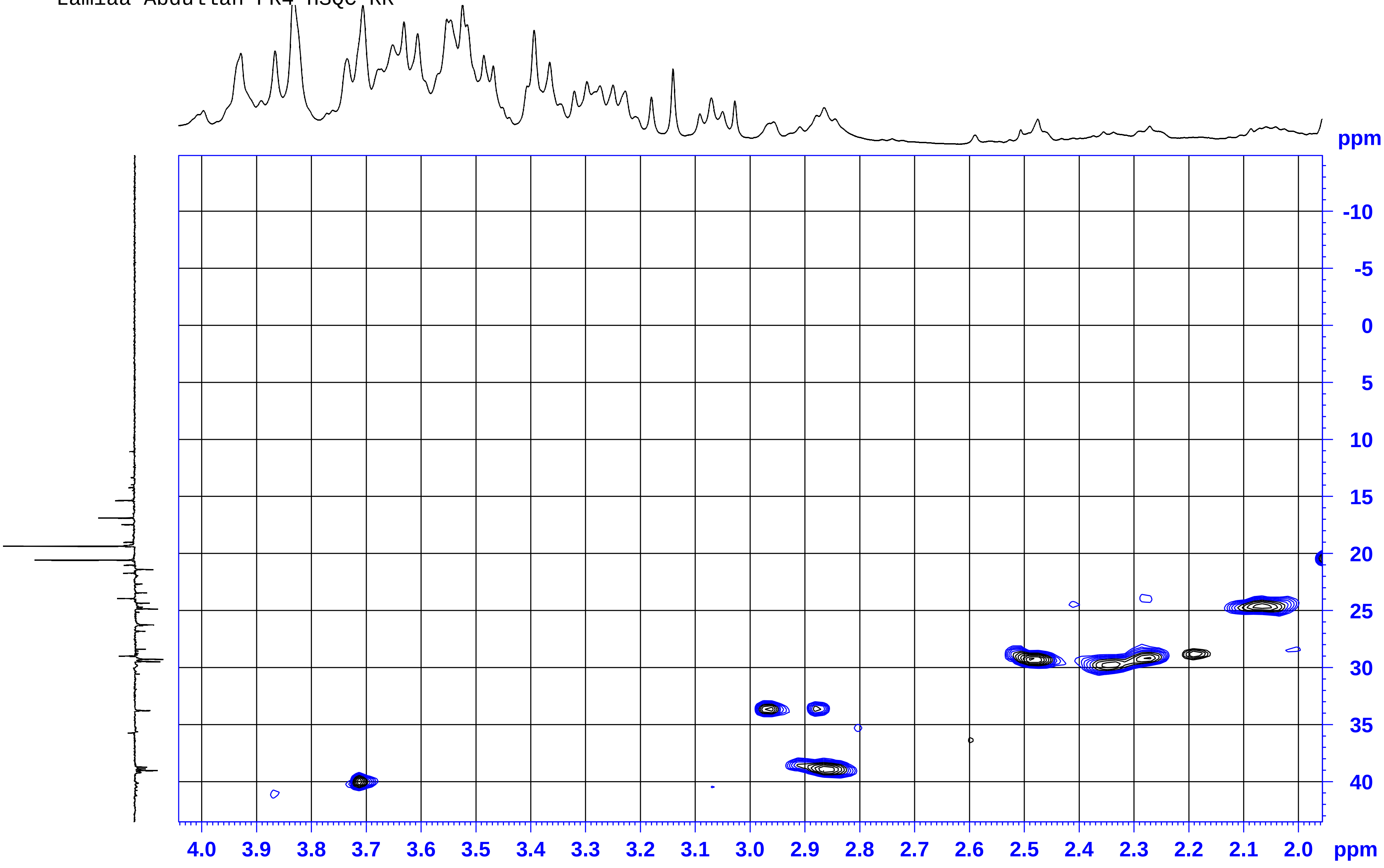

Lamiaa Abdullah-PR4-HSQC-RR

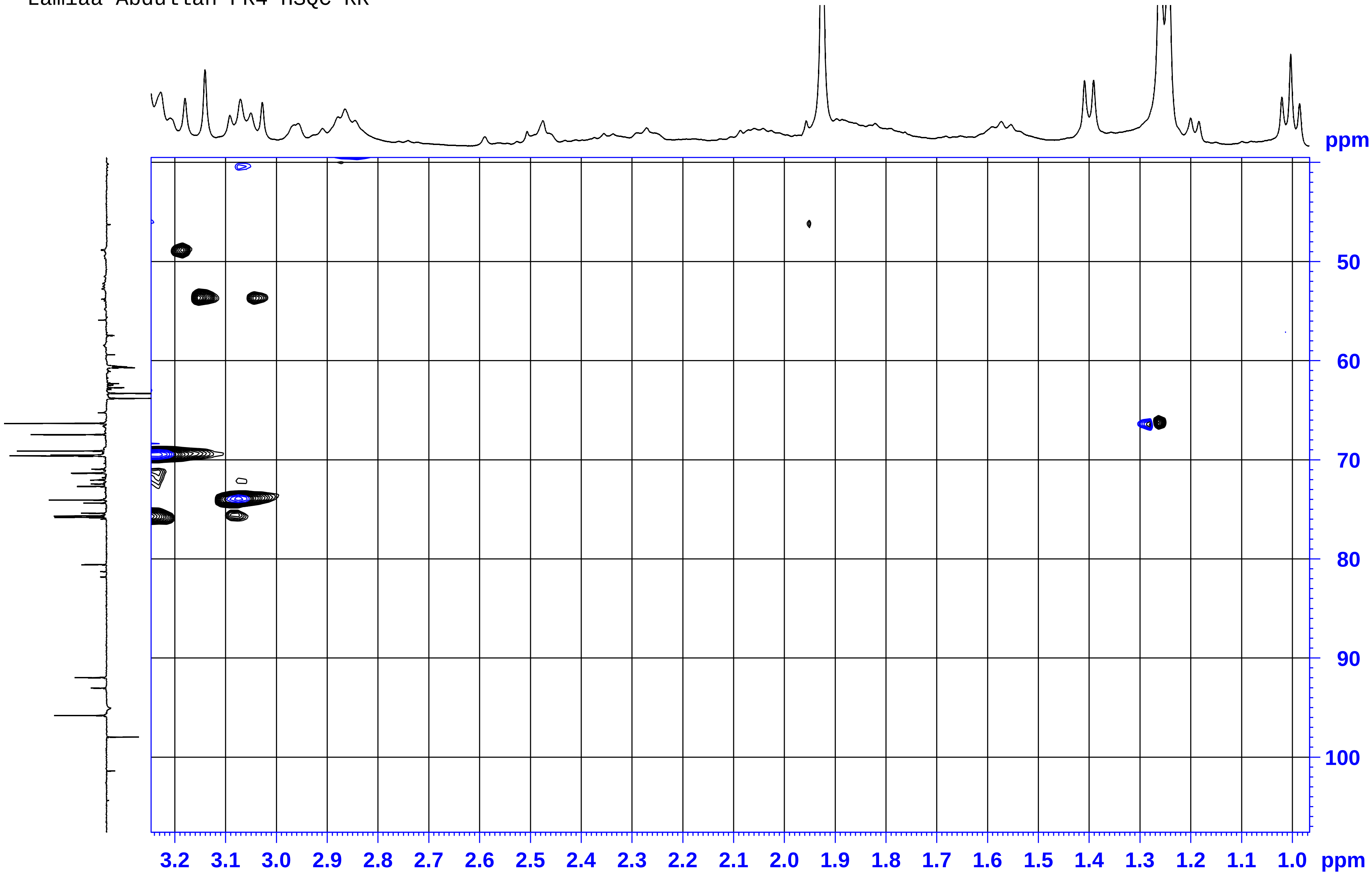

Lamiaa Abdullah-PR4-HSQC-RR

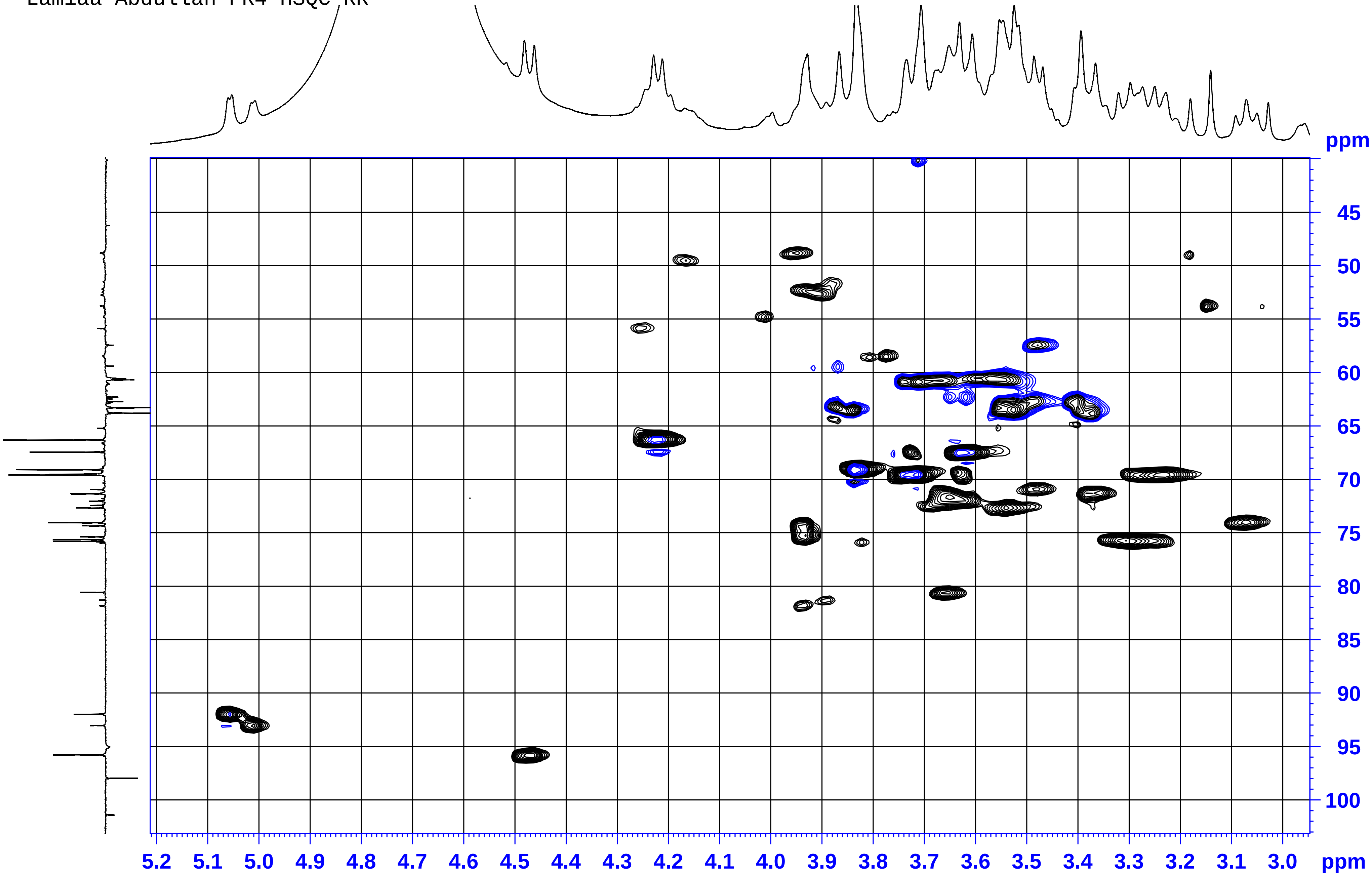

Lamiaa Abdullah-PR4-HSQC-RR

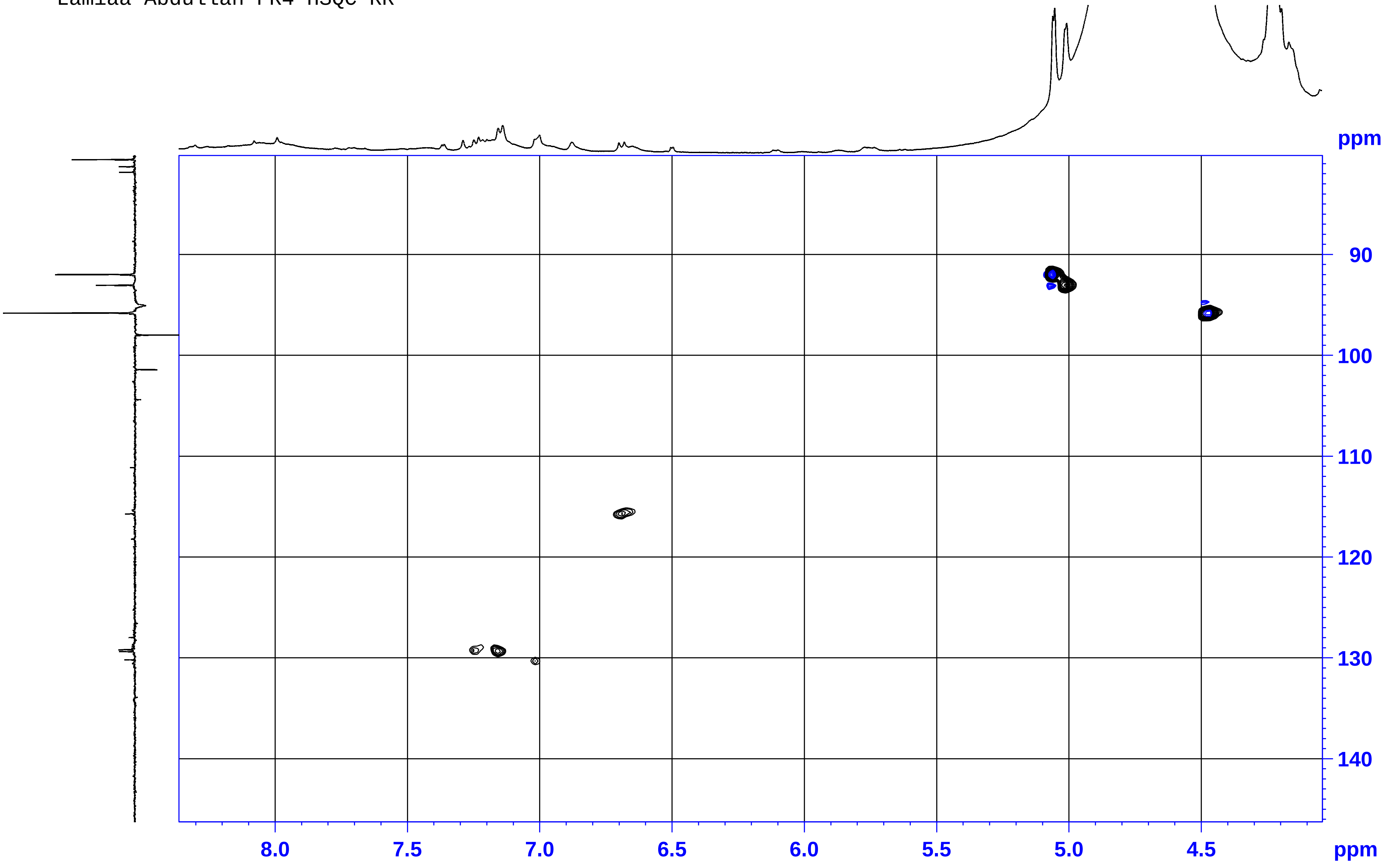

Supplement: Supplementary file 4 [file 12934_2026_3067_MOESM4_ESM.pdf]
