# Supplementary material for: Bio-process development for sustainable, large-scale production of LAB-oligosaccharides as a potential anti-colon-cancer metabolites produced by Lactobacillus acidophilus 20079
Source: Microb Cell Fact. 2026 Aug 12;25:168. doi: 10.1186/s12934-026-03067-x (PMC13474794; doi:10.1186/s12934-026-03067-x)

Lamiaa Abdullah-PR4-HMBC-RR

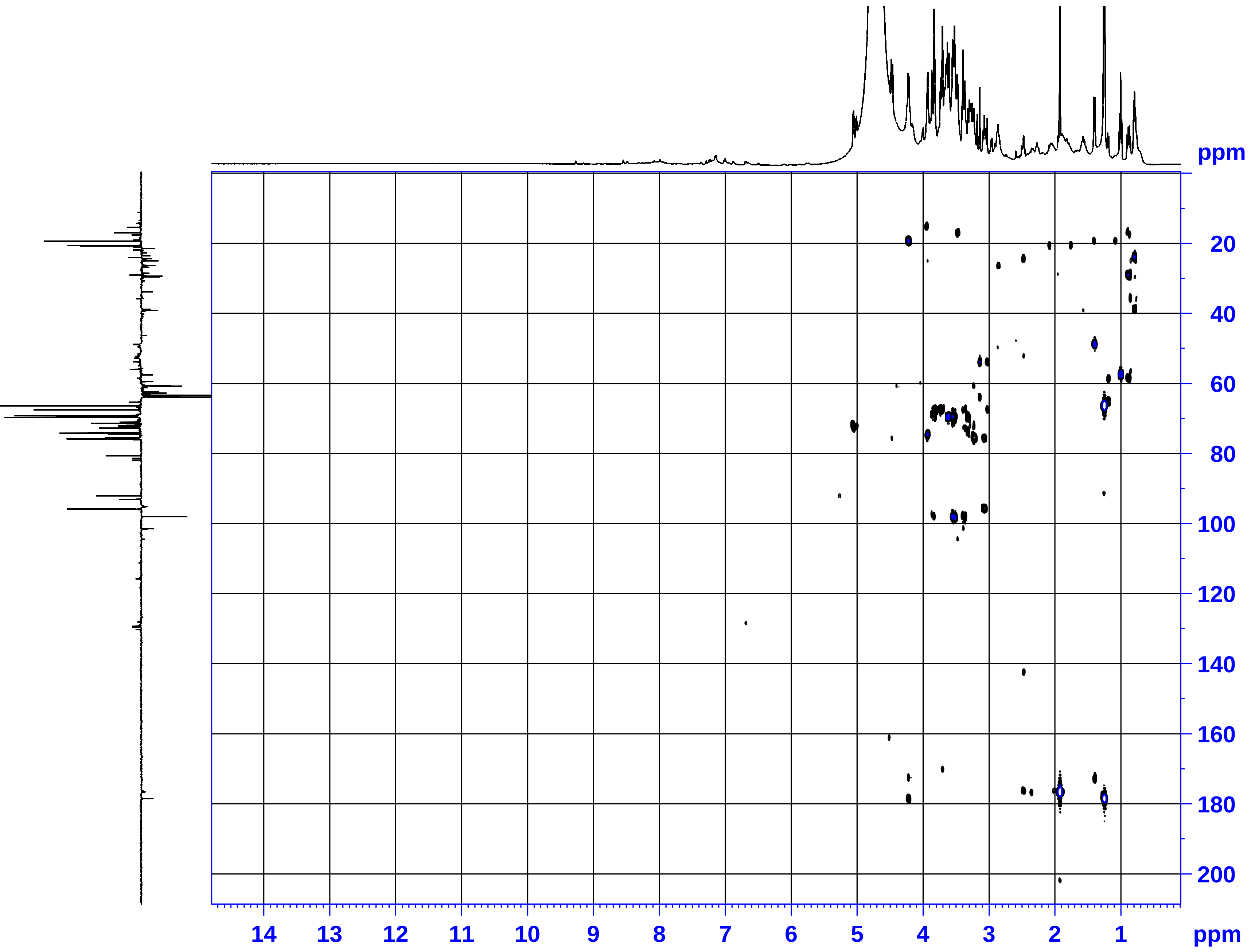

BRUKER

Faculty of Pharmacy

```
Current Data Parameters
NAME       Lamiaa Abdullah-PR4-2D-RR
EXPNO                     12
PROCNO                     1

F2 - Acquisition Parameters
Date_               20240626
Time                   18.20 h
INSTRUM                spect
PROBHD     Z108618_0945 (
PULPROG      hmbcgplpndqf
TD                      2048
SOLVENT                  D2O
NS                        34
DS                        16
SWH                 6798.315 Hz
FIDRES              6.638980 Hz
AQ                 0.1506256 sec
RG                    197.77
DW                    73.548 usec
DE                      6.50 usec
TE                     295.4 K
CNST2            145.0000000
CNST13            10.0000000
D0                0.00000300 sec
D1                1.50000000 sec
D2                0.00344828 sec
D6                0.05000000 sec
D16               0.00020000 sec
IN0               0.00001990 sec
TDav                       1
SFO1             400.2025213 MHz
NUC1                      1H
P1                     13.50 usec
P2                     27.00 usec
PLW1             13.00000000 W
SFO2             100.6404331 MHz
NUC2                     13C
P3                     10.00 usec
PLW2             47.00000000 W
GPNAM[1]          SMSQ10.100
GPZ1                   50.00 %
GPNAM[2]          SMSQ10.100
GPZ2                   30.00 %
GPNAM[3]          SMSQ10.100
GPZ3                   40.10 %
P16                  1000.00 usec

F1 - Acquisition parameters
TD                       128
SFO1                100.6404 MHz
FIDRES            392.587952 Hz
SW                   249.657 ppm
FnMODE                    QF

F2 - Processing parameters
SI                      2048
SF               400.2000000 MHz
WDW                     SINE
SSB          0
LB           0 Hz
GB           0
PC                      1.40

F1 - Processing parameters
SI                      1024
MC2                       QF
SF               100.6303700 MHz
WDW                     SINE
SSB          0
LB           0 Hz
GB           0
```

Lamiaa Abdullah-PR4-HMBC-RR

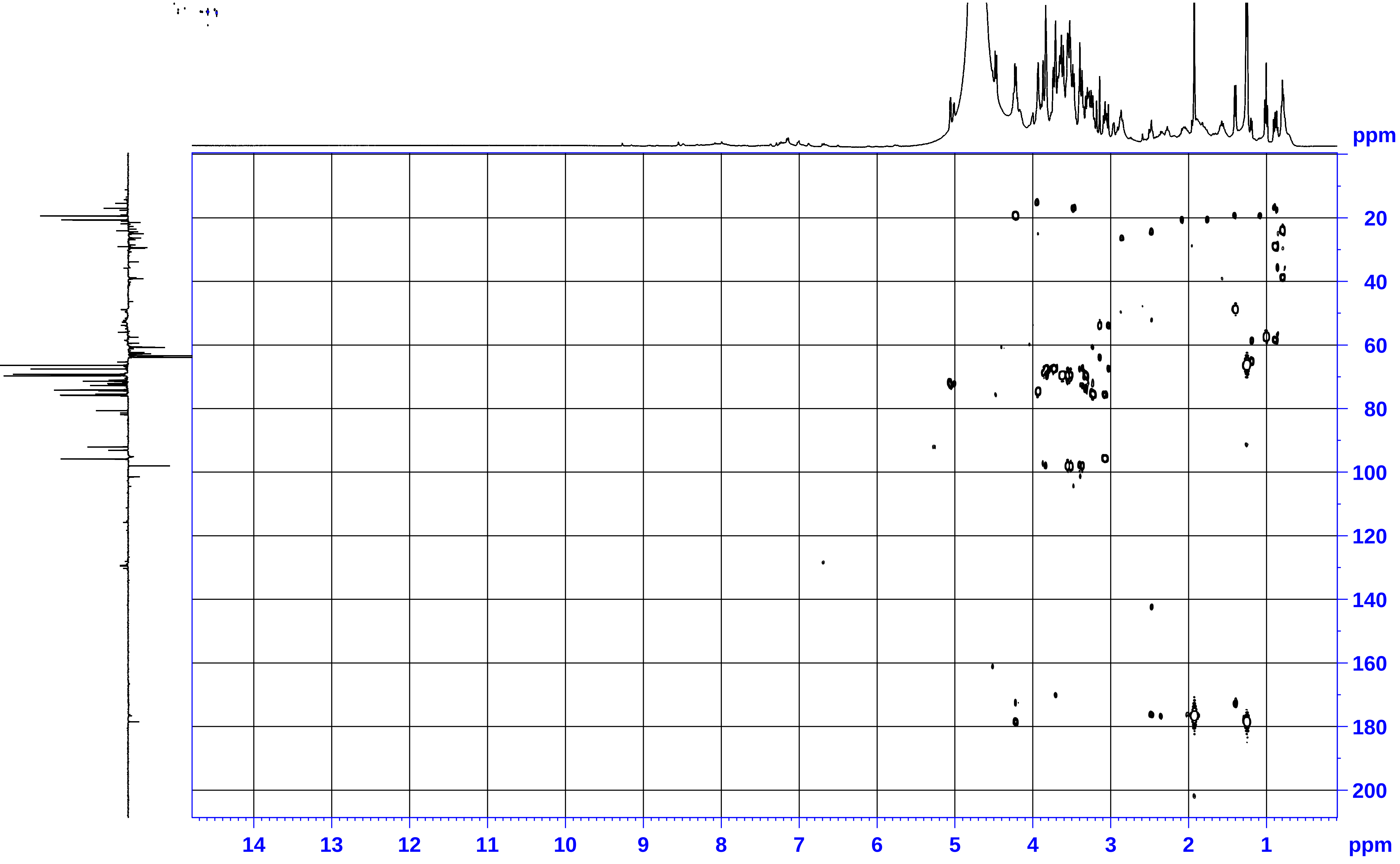

Lamiaa Abdullah-PR4-HMBC-RR

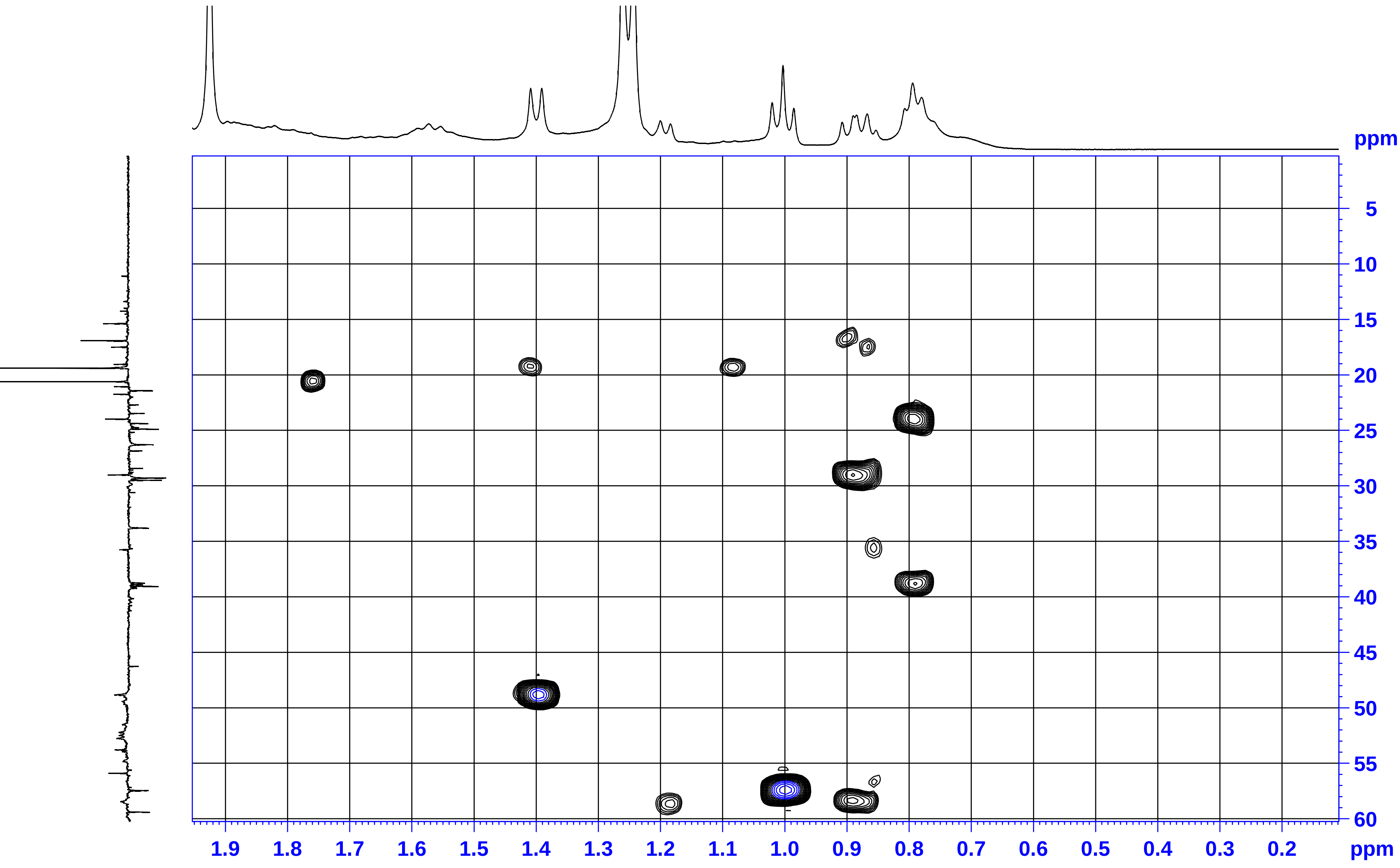

Lamiaa Abdullah-PR4-HMBC-RR

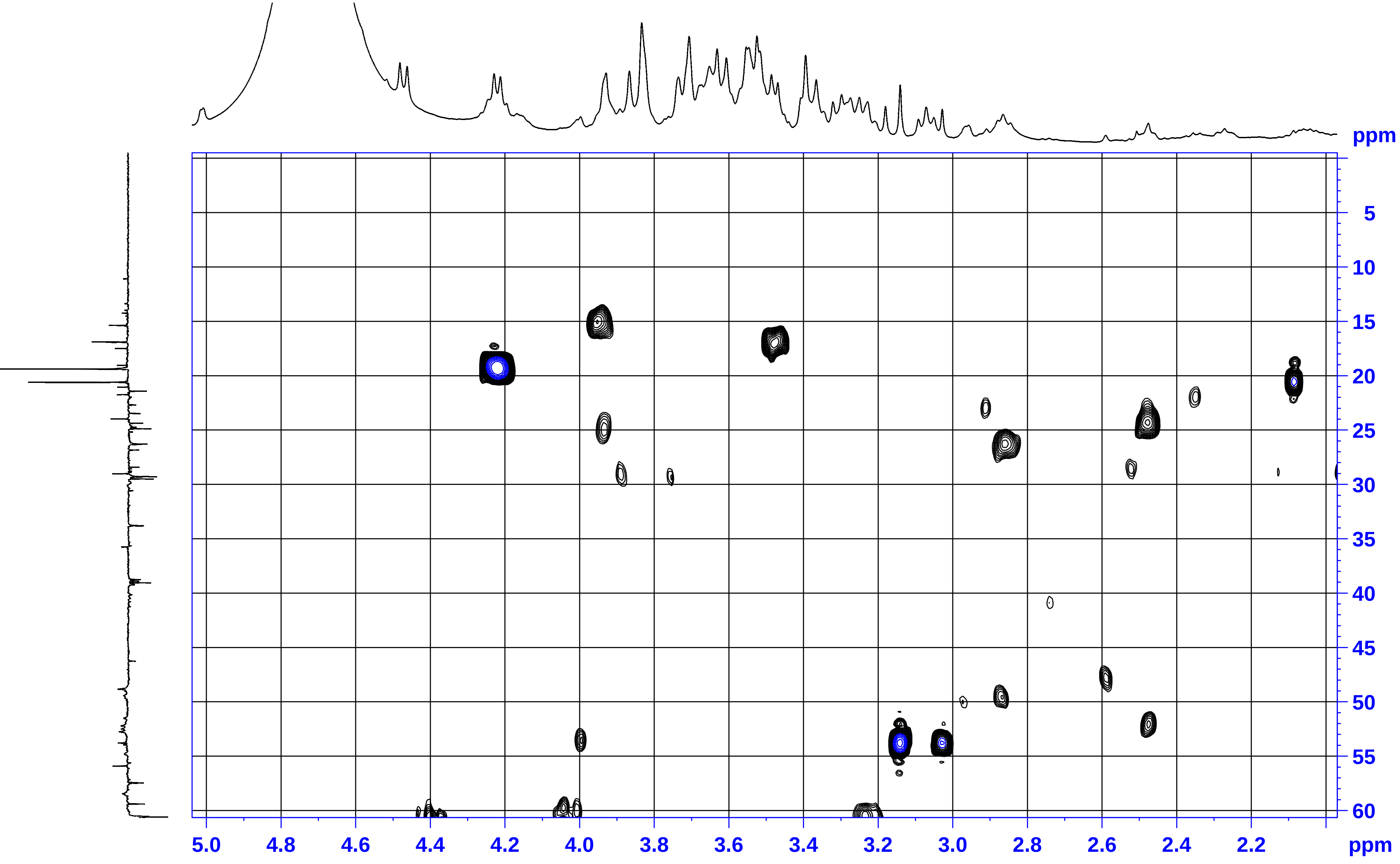

Lamiaa Abdullah-PR4-HMBC-RR

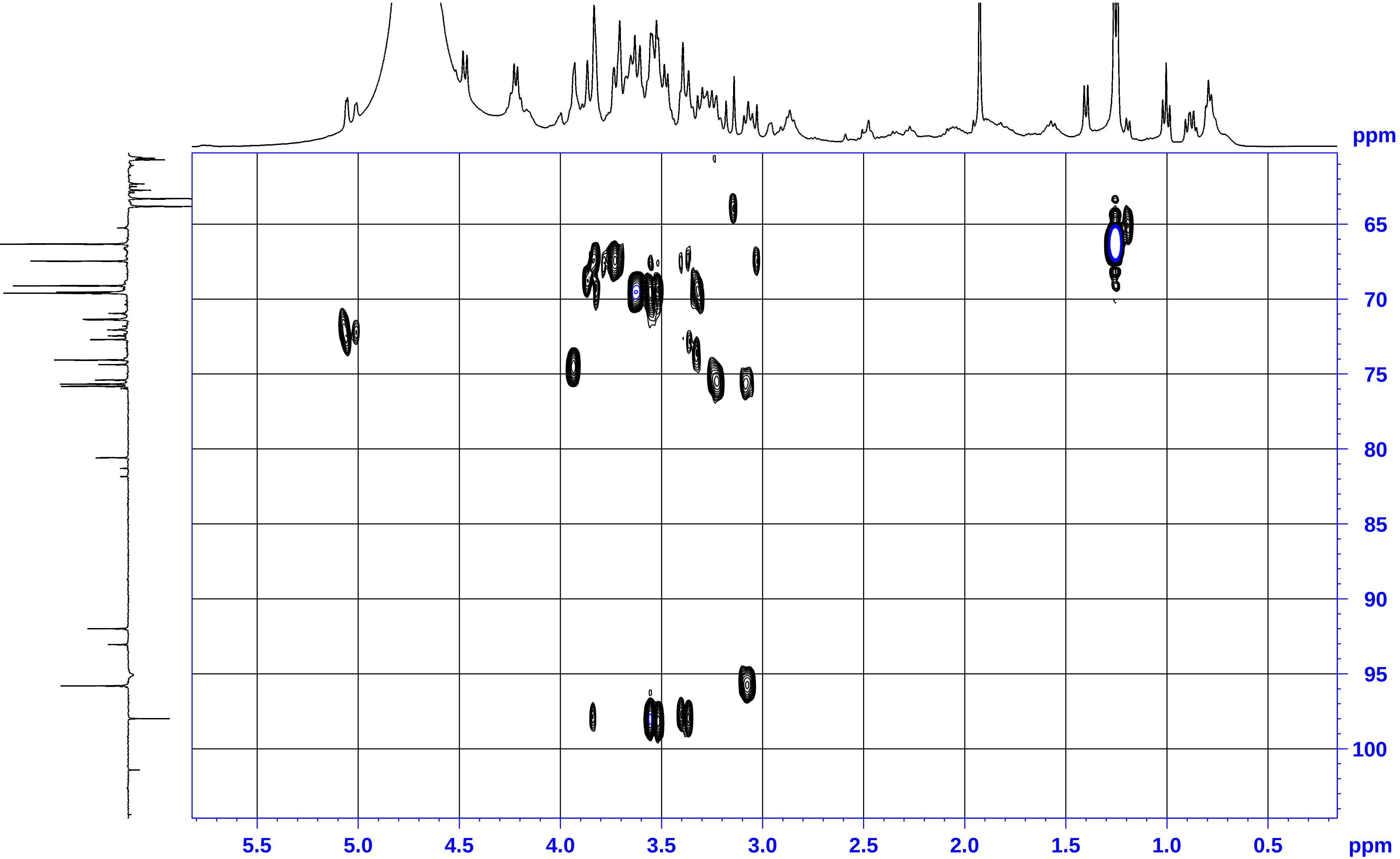

Lamiaa Abdullah-PR4-HMBC-RR

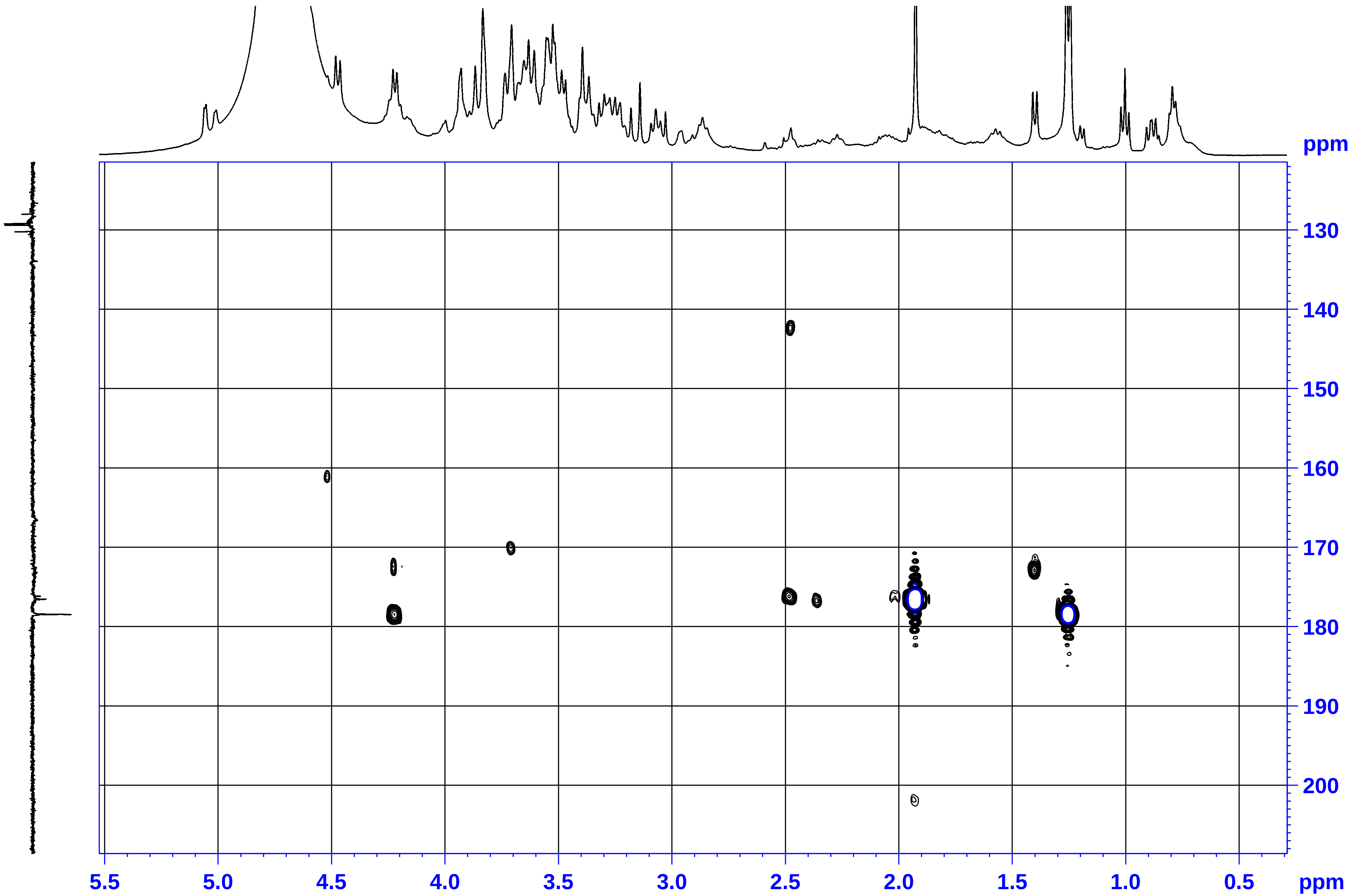

Lamiaa Abdullah-PR4-HMBC-RR

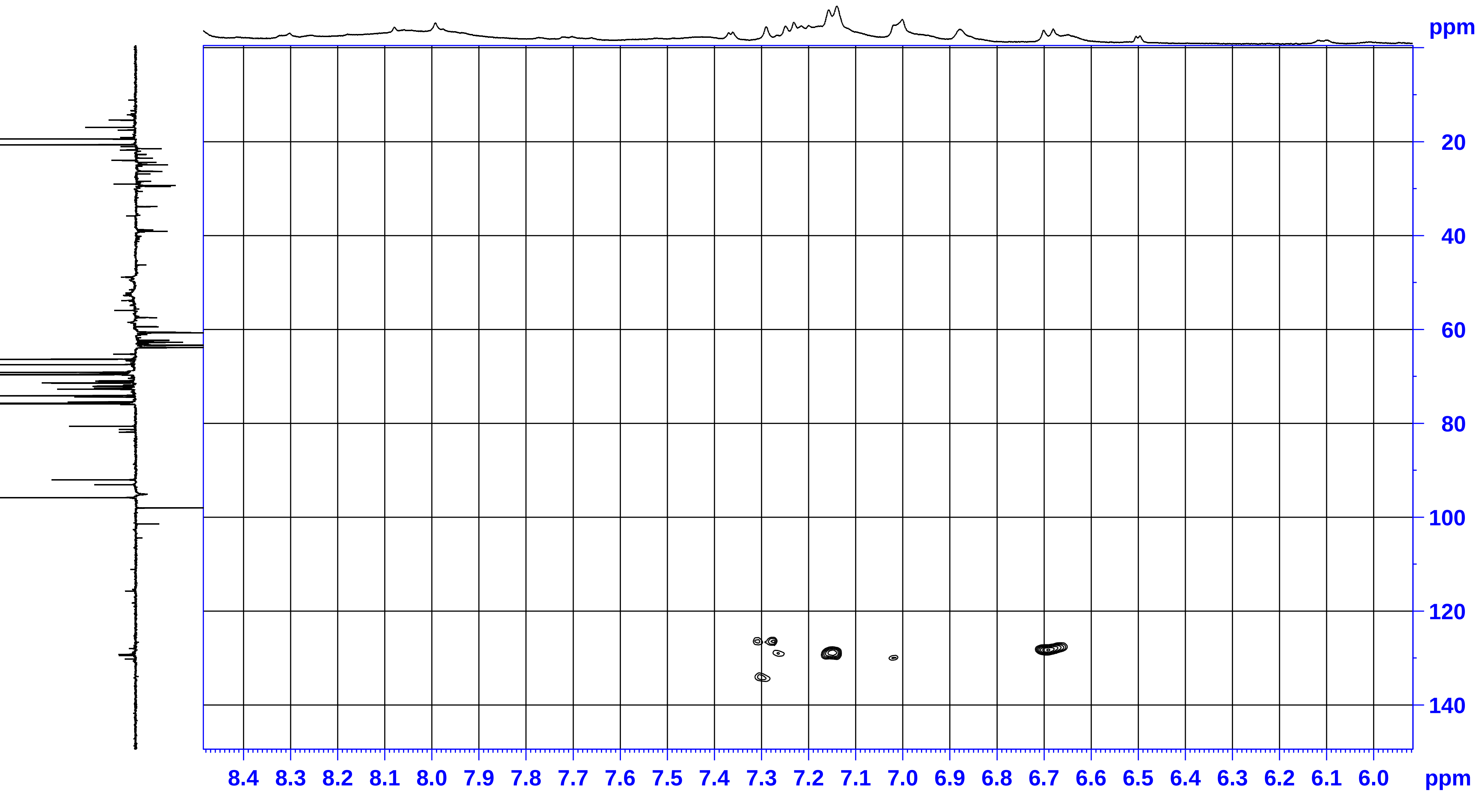

Supplement: Supplementary file 5 [file 12934_2026_3067_MOESM5_ESM.pdf]
