# Supplementary material for: Bio-process development for sustainable, large-scale production of LAB-oligosaccharides as a potential anti-colon-cancer metabolites produced by Lactobacillus acidophilus 20079
Source: Microb Cell Fact. 2026 Aug 12;25:168. doi: 10.1186/s12934-026-03067-x (PMC13474794; doi:10.1186/s12934-026-03067-x)

Lamiaa Abdullah-PR4-COSY-RR

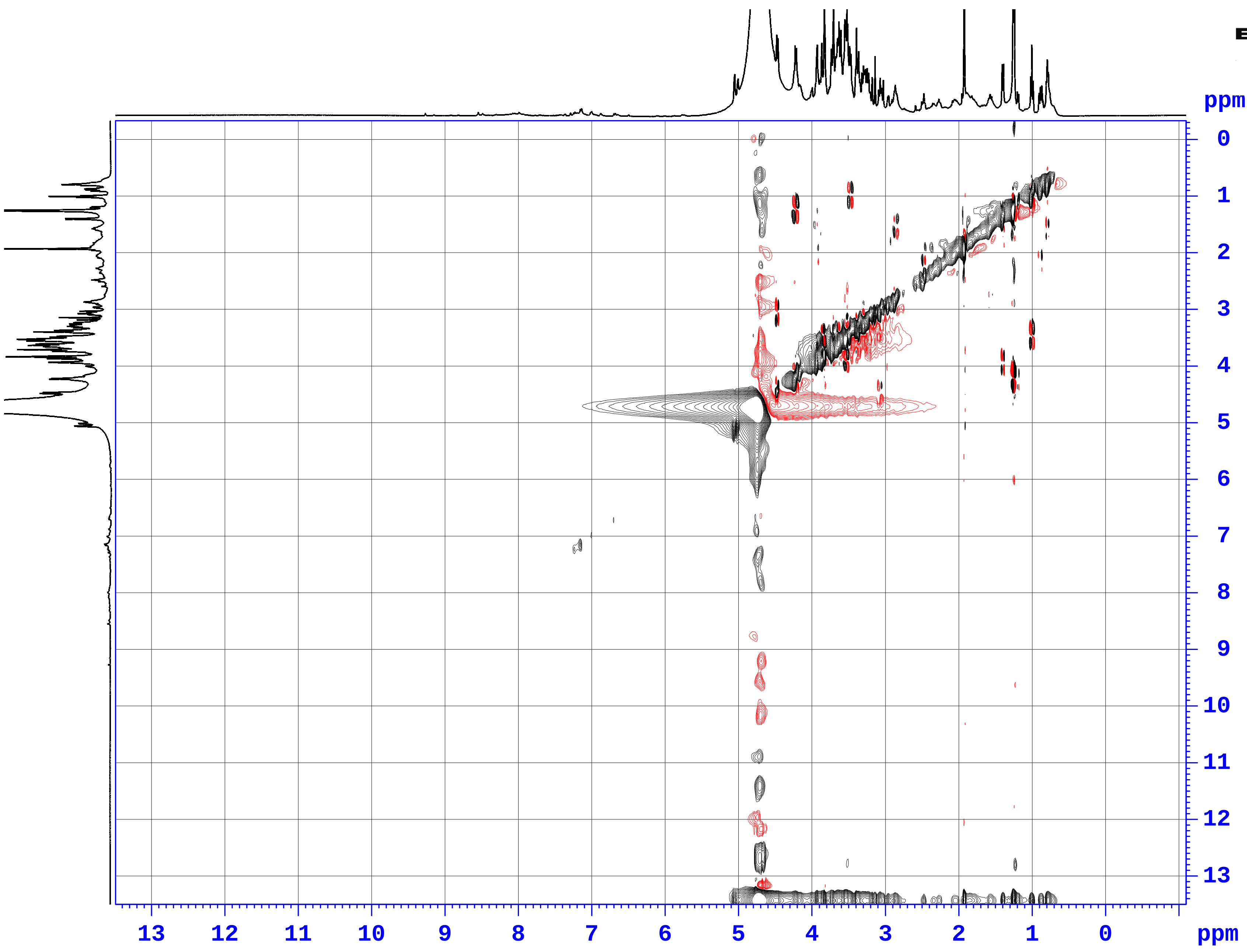


```
Current Data Parameters
NAME      Lamiaa Abdullah-PR4-2D-RR
EXPNO                  11
PROCNO                  1

F2 - Acquisition Parameters
Date_            20240626
Time                16.00 h
INSTRUM             spect
PROBHD   Z108618_0945 (
PULPROG          cosydfph
TD                   2048
SOLVENT               D2O
NS                     30
DS                     16
SWH              6398.112 Hz
FIDRES           6.248156 Hz
AQ              0.1600472 sec
RG                  17.41
DW                 78.148 usec
DE                   6.50 usec
TE                  295.8 K
D0             0.00006091 sec
D1             2.00000000 sec
D13            0.00000400 sec
IN0            0.00015620 sec
TDav                    1
SFO1          400.2022011 MHz
NUC1                   1H
P1                  13.50 usec
PLW1          13.00000000 W

F1 - Acquisition parameters
TD                    128
SFO1             400.2022 MHz
FIDRES         100.032013 Hz
SW                 15.997 ppm
FnMODE        States-TPPI

F2 - Processing parameters
SI                   1024
SF            400.2000000 MHz
WDW                 QSINE
SSB                     2
LB       0 Hz
GB       0
PC                   1.40

F1 - Processing parameters
SI                   1024
MC2           States-TPPI
SF            400.2000000 MHz
WDW                 QSINE
SSB                     2
LB       0 Hz
GB       0
```

Lamiaa Abdullah-PR4-COSY-RR

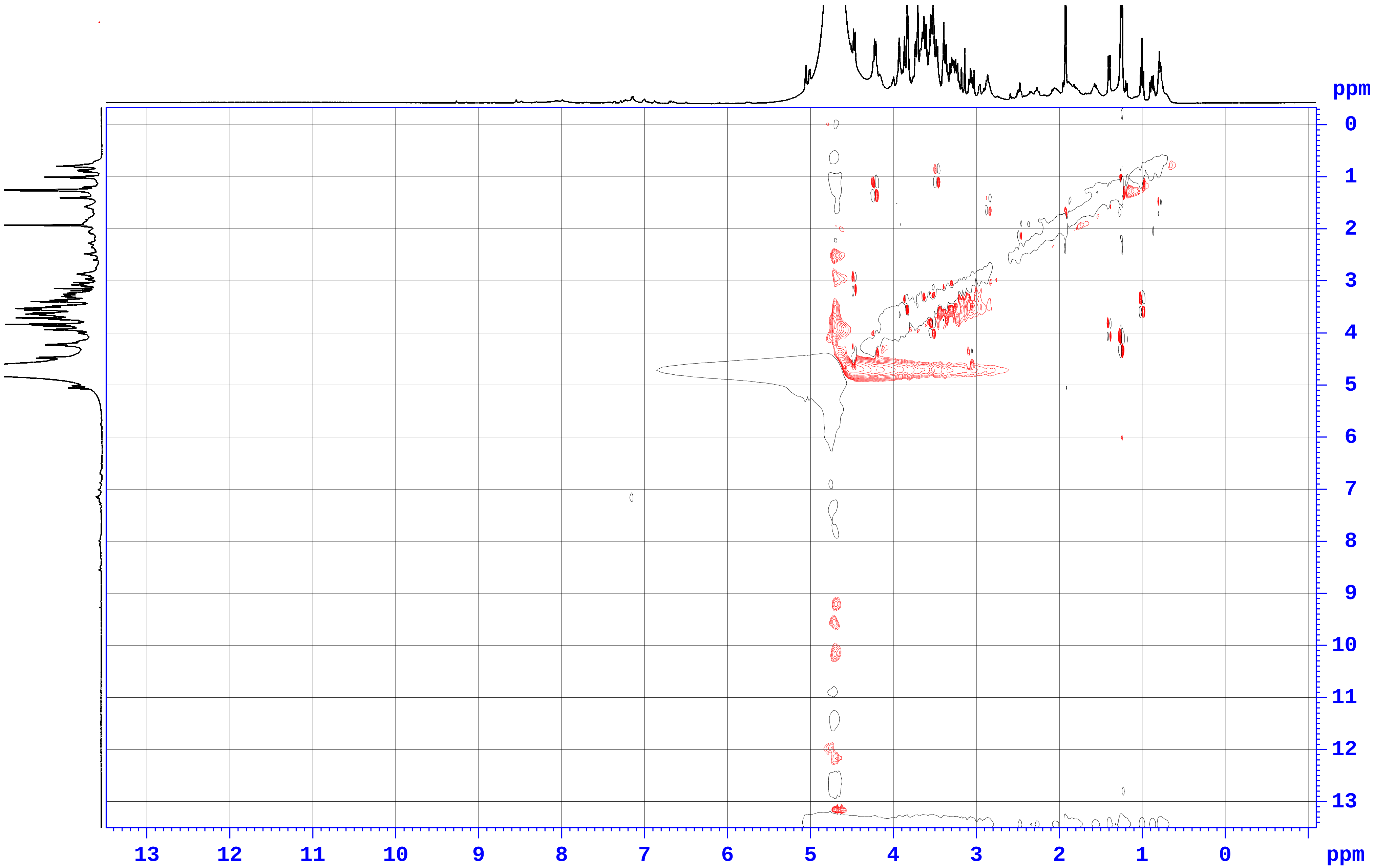

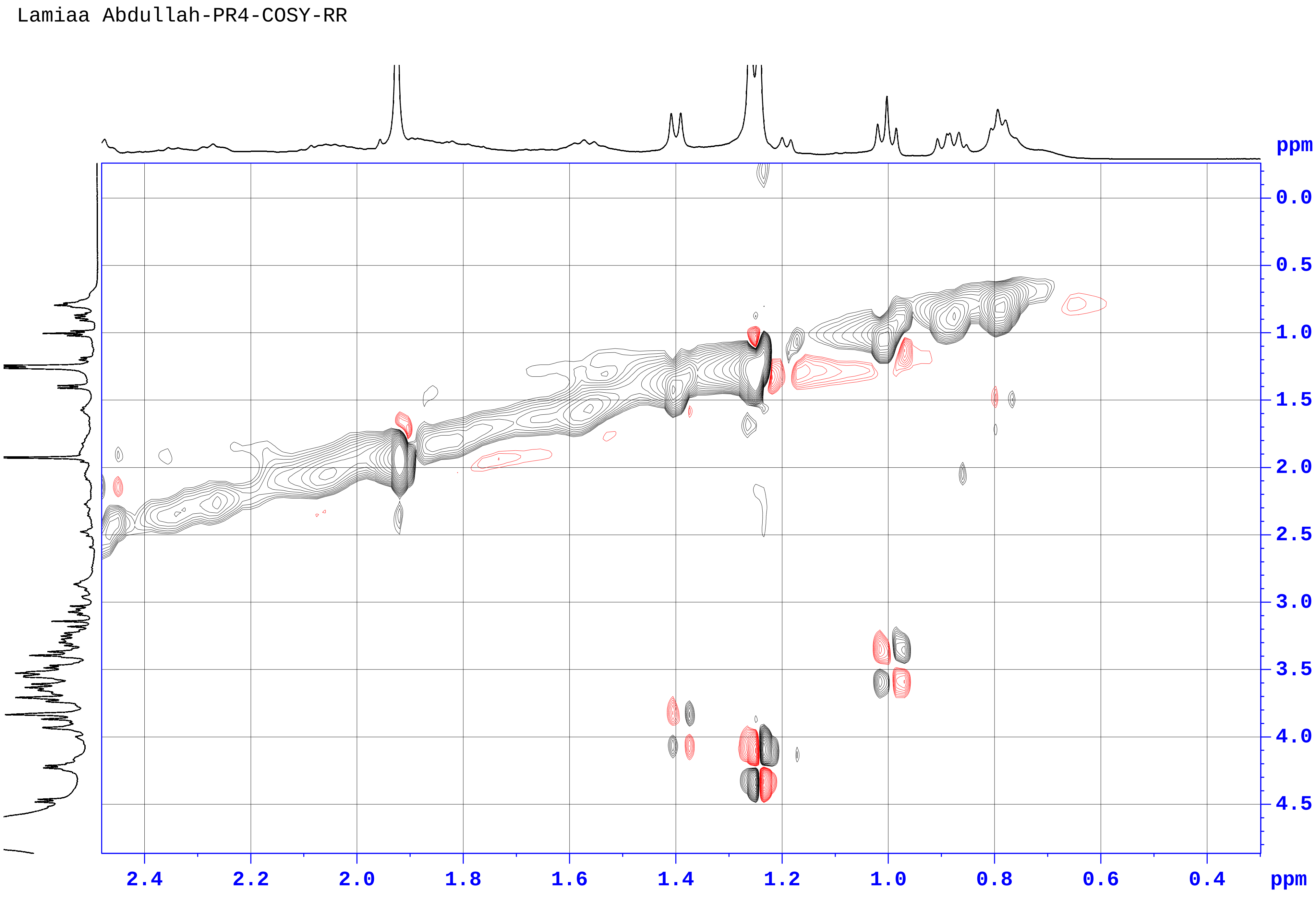
Lamiaa Abdullah-PR4-COSY-RR
ppm
0.0
0.5
1.0
1.5
2.0
2.5
3.0
3.5
4.0
4.5
2.4
2.2
2.0
1.8
1.6
1.4
1.2
1.0
0.8
0.6
0.4
ppm

Lamiaa Abdullah-PR4-COSY-RR

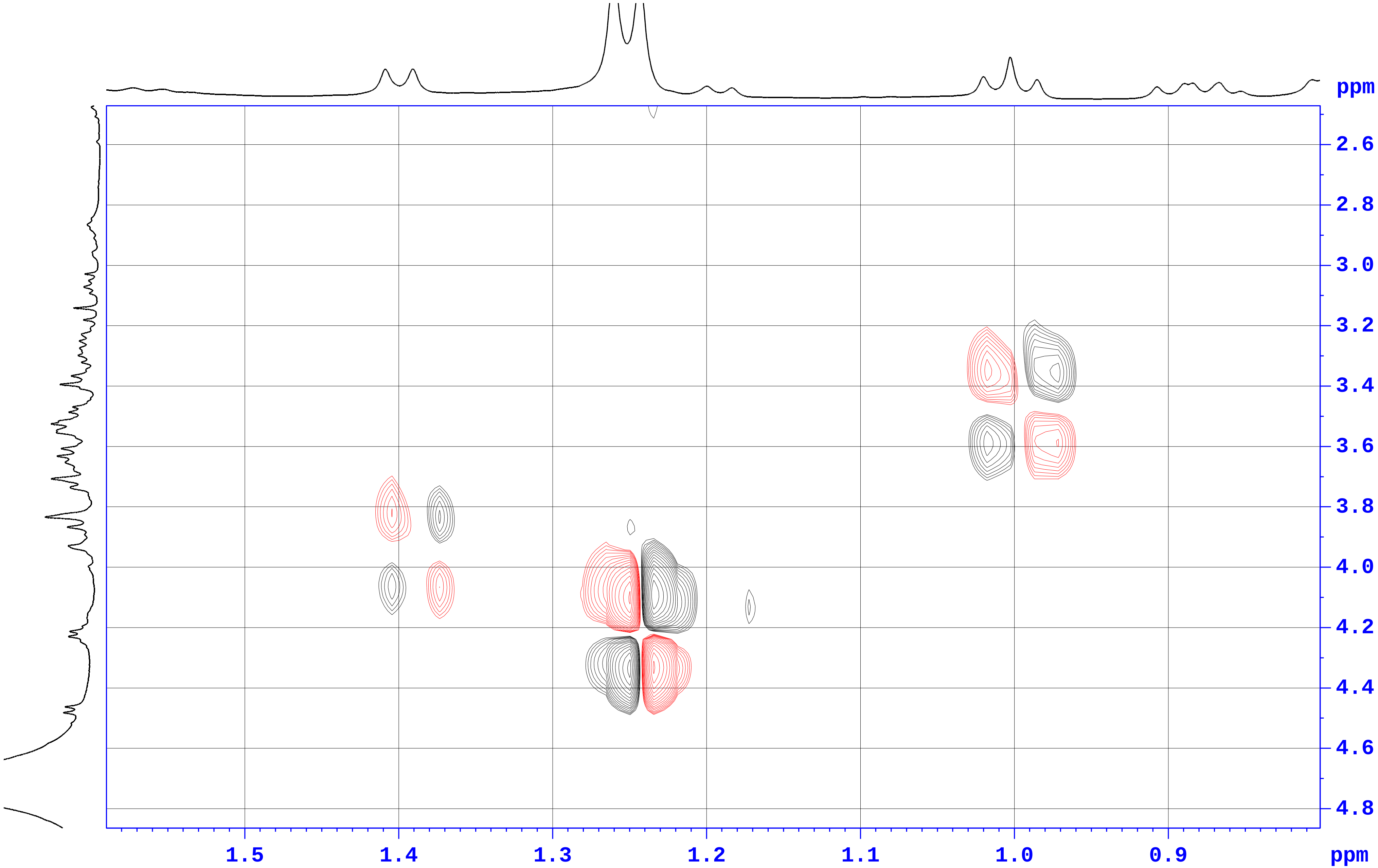

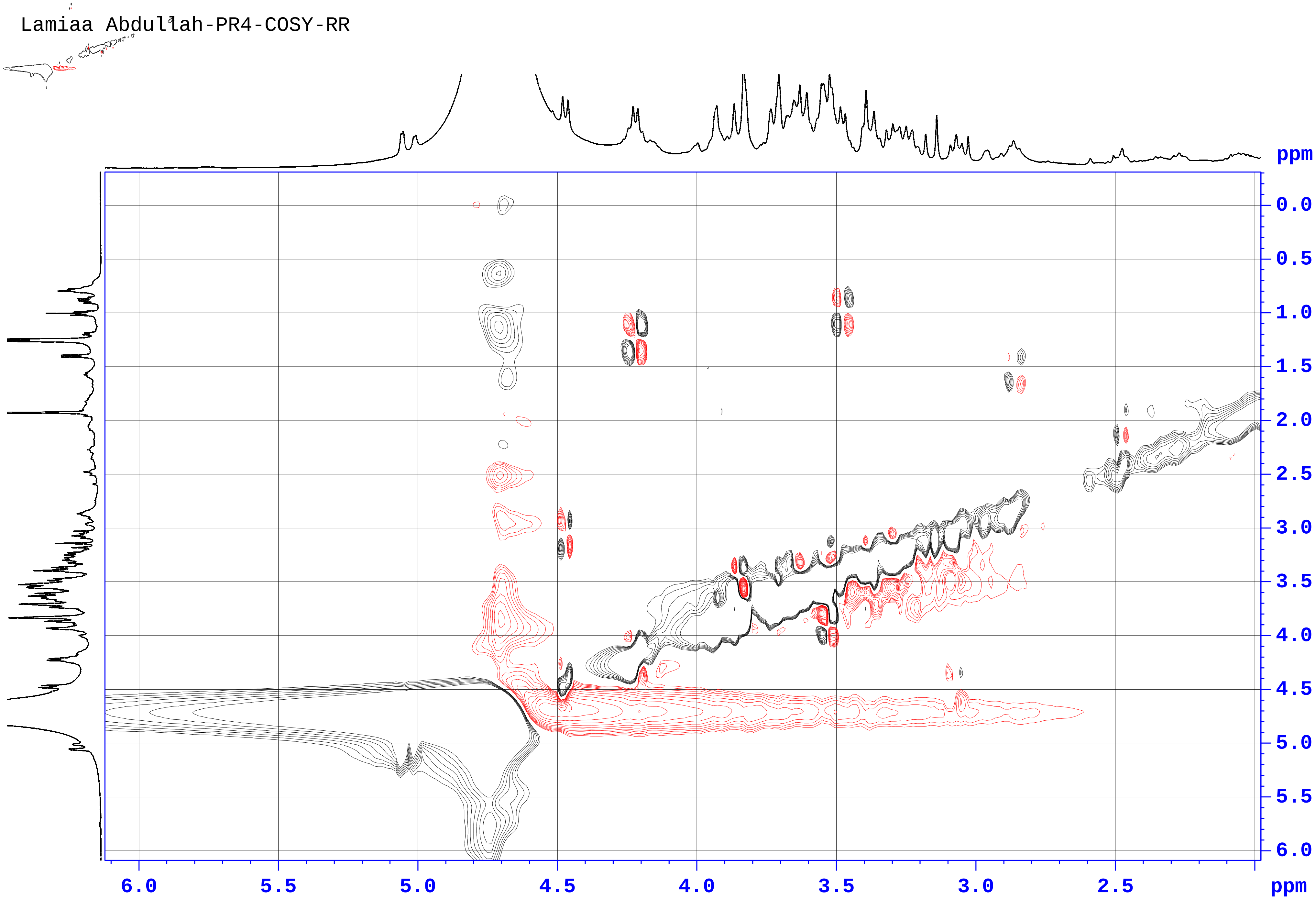
Lamiaa Abdullah-PR4-COSY-RR
ppm
0.0
0.5
1.0
1.5
2.0
2.5
3.0
3.5
4.0
4.5
5.0
5.5
6.0
6.0
5.5
5.0
4.5
4.0
3.5
3.0
2.5
ppm

Lamiaa Abdullah-PR4-COSY-RR

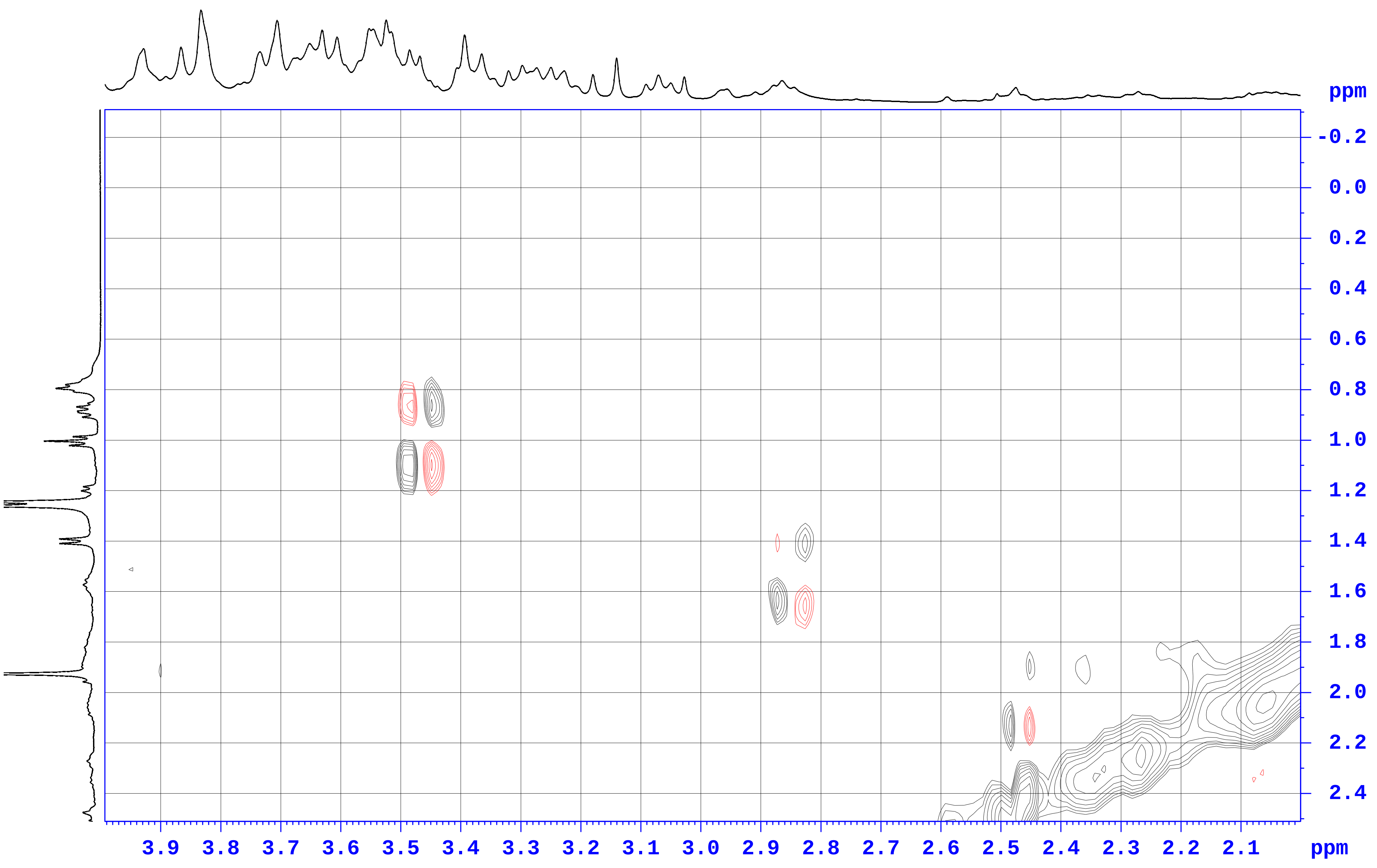

Lamiaa Abdullah-PR4-COSY-RR

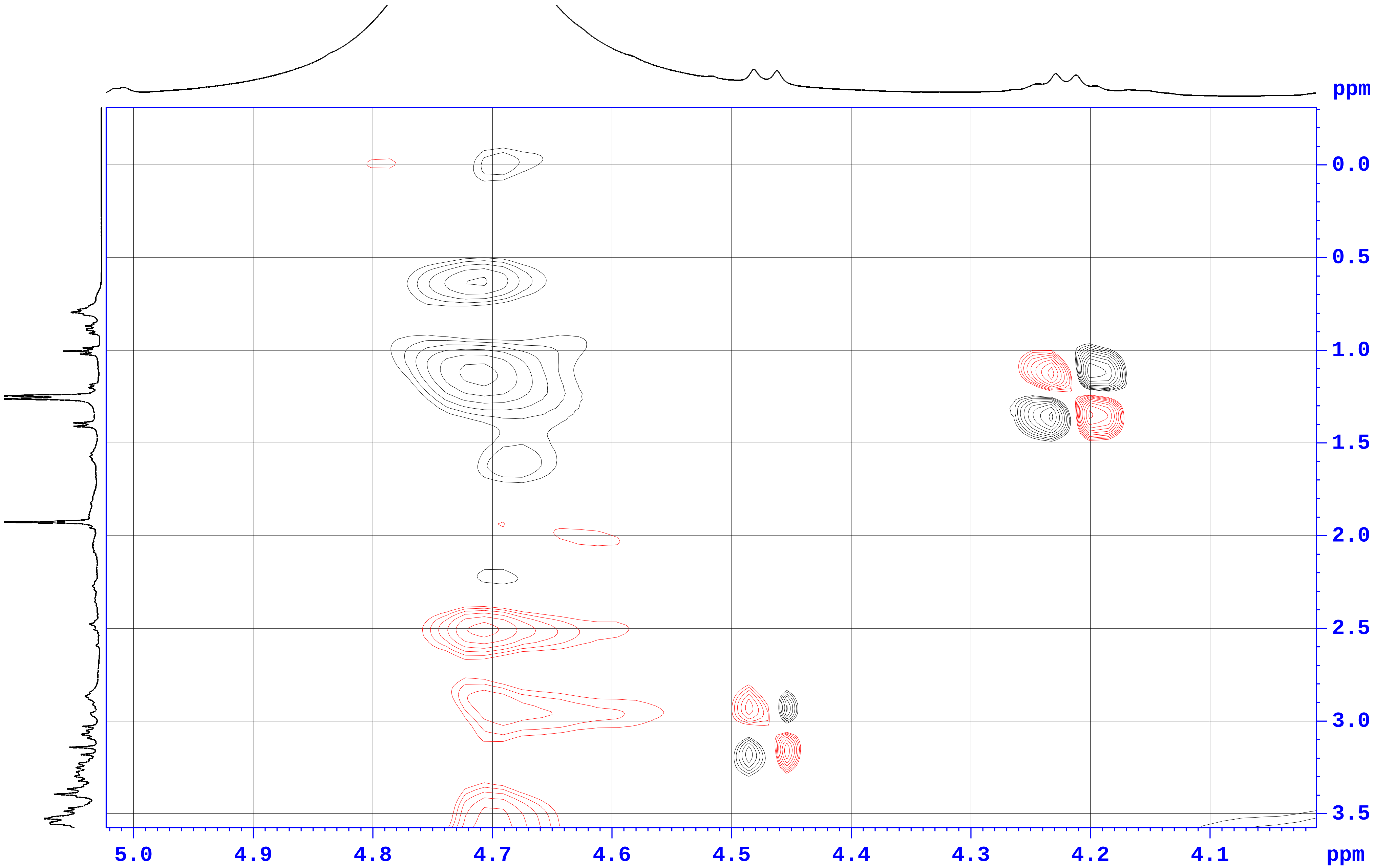

Lamiaa Abdullah-PR4-COSY-RR

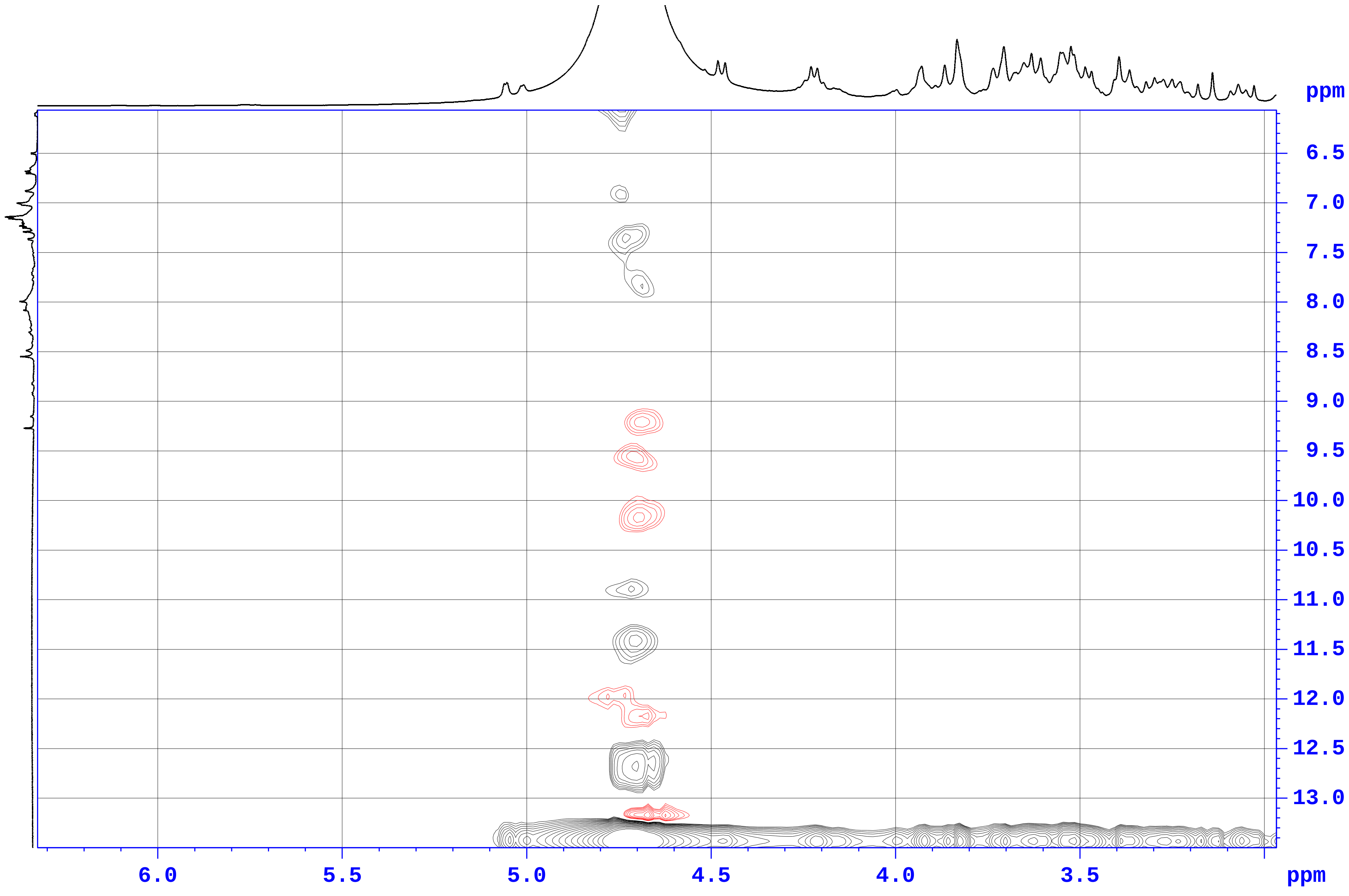

Supplement: Supplementary file 6 [file 12934_2026_3067_MOESM6_ESM.pdf]
