# Supplementary material for: Bio-process development for sustainable, large-scale production of LAB-oligosaccharides as a potential anti-colon-cancer metabolites produced by Lactobacillus acidophilus 20079
Source: Microb Cell Fact. 2026 Aug 12;25:168. doi: 10.1186/s12934-026-03067-x (PMC13474794; doi:10.1186/s12934-026-03067-x)
Supplement: Supplementary file 7 [file 12934_2026_3067_MOESM7_ESM.docx]

**Table S1: ^13^C NMR data for the oligosaccharide mix**

| Type of carbon | Chemical shift (ppm) |
| --- | --- |
| Quaternary | 178.6 |
| Quaternary | 176.5 |
| Quaternary | 101.5 |
| Quaternary | 98.1 |
| CH | 95.8 |
| CH | 93.9 |
| CH | 91.9 |
| CH | 80.6 |
| CH | 76.0 |
| CH | 74.3 |
| CH | 74.0 |
| CH | 72.7 |
| CH | 71.7 |
| CH | 70.1 |
| CH | 69.4 |
| CH | 67.7 |
| CH | 66.4 |
| CH_2_ | 63.8 |
| CH_2_ | 63.4 |
| CH_2_ | 60.6 |
| CH_3_ | 20.7 |
| CH_3_ | 19.4 |
| CH_3_ | 17.0 |

**Table S2:** ^1^H NMR key data and its HSQC correlations

| ^1^H NMR chemical shift (ppm) | HSQC correlations (ppm) |
| --- | --- |
| 5.17 (d) | 91.9 |
| 5.05 (d) | 93.9 |
| 4.48 (d) | 95.8 |
| 4.24 (d) | 67.7 |
| 3.6-3.7 (m) | 80.6 |
| 1.92 (s) | 20.7 |
| 1.4 (d) | 17.0 |
| 1.25 (d) | 19.4 |

d: doublet, s: singlet, m: multiplet.
